# Identification of VVD-214/RO7589831, a Clinical-Stage, Covalent Allosteric Inhibitor of WRN Helicase for the Treatment of MSI-High Cancers

**DOI:** 10.1021/acs.jmedchem.5c01805

**Published:** 2025-09-18

**Authors:** Shota Kikuchi, Jason C. Green, Don C. Rogness, Betty Lam, Zachary A. Owyang, Robert D. Malmstrom, Ali Tabatabaei, Aaron N. Snead, Melissa A. Hoffman, Steffen M. Bernard, Paige Ashby, Kelsey N. Lamb, Benjamin D. Horning, Kristen A. Baltgalvis, Kent T. Symons, Thomas A. Glaza, Chu-Chiao Wu, Xiaodan Song, Martha K. Pastuszka, John J. Sigler, Jonathan Pollock, Laurence Burgess, Gabriel M. Simon, Matthew P. Patricelli, David S. Weinstein

**Affiliations:** 599592 Vividion Therapeutics Inc, San Diego, California 92121, United States

## Abstract

Werner syndrome helicase (WRN) has emerged as a compelling therapeutic target for microsatellite instability-high (MSI-H) cancers, owing to its selective dependency on the helicase activity of WRN. Despite the inherent challenges in targeting helicases, our chemoproteomics approach enabled the identification of compounds that covalently engage C727 within an allosteric pocket of WRN, thereby inhibiting its ability to unwind DNA. Through optimization of each molecular component, particularly focusing on the vinyl sulfone warhead and C2 substitution at the pyrimidine core, an optimal balance of intrinsic reactivity, inhibitory potency, and metabolic stability was achieved, culminating in the identification of VVD-214/RO7589831. This process underscored the tunability of the vinyl sulfone warhead and its effectiveness in covalent drug discovery. VVD-214 induced tumor regression in MSI-H colorectal cancer models and is being evaluated as a promising therapeutic candidate for MSI-H cancers.

## Graphical abstract

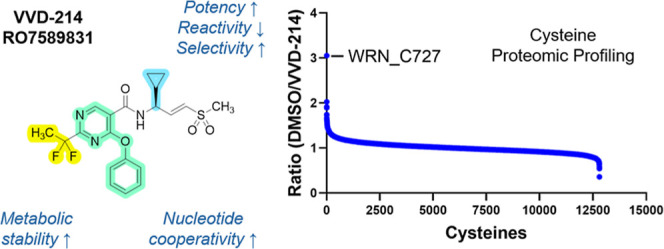

## Introduction

Microsatellite instability-high (MSI-H) is a genetic characteristic observed in various cancer types, with the highest prevalence in colorectal, endometrial, and gastric adenocarcinomas.^1,2^ This feature is typified by expanded TA-dinucleotide repeats within microsatellite regions and the formation of aberrant DNA secondary structures. The MSI-H phenotype results from mutation or epigenetic silencing of genes involved in the DNA mismatch repair (MMR) system.^3^ Notably, cancers exhibiting MSI-H generally have a more favorable prognosis and show enhanced responsiveness to immunotherapy, one of the standards of care for this disease.^4–7^ However, the efficacy of these treatments can be constrained by intrinsic or acquired resistance^8–11^ .

In 2019, four separate studies were published, which support the conclusion that there is a selective dependency of MSI-H cancer cell lines on Werner syndrome helicase (WRN), a member of the RecQ helicase family.^12–15^ WRN has diverse roles in DNA replication, double-strand break repair, transcription, and telomere maintenance.^16–19^ The helicase activity of WRN is vital in resolving aberrant DNA secondary structures in MSI-H cancer cells.^20^ A loss of function in WRN results in double-stranded DNA breaks, chromosomal fragmentation, and cell death, specifically in MSI-H cancer cells, but not in normal cells. This synthetic lethal interaction between WRN and MSI-H cancer suggests that WRN inhibition could be a promising novel therapeutic strategy for treating MSI-H cancers, which would complement the current standard of care for MSI-H cancers.

Our team has reported the chemical proteomic-enabled discovery of the clinical stage molecule VVD-133214 (also known as **VVD-214** and RO7589831), a covalent allosteric inhibitor of WRN.^21^ Characterization of hits showing selective binding to the cysteine residue (C727) of WRN in chemoproteomics screens led to the identification of both nucleotide-competitive and nucleotide-cooperative compounds. Optimization of the nucleotide-cooperative series led to the identification of **VVD-214**, which demonstrated synthetic lethality through covalent inhibition of WRN, triggering double-stranded DNA breaks, nuclear swelling, and cell death in MSI-H, but not in microsatellite stable (MSS) cells. **VVD-214** was well-tolerated in mice and led to significant tumor regression in MSI-H colorectal cancer models. **VVD-214** is currently being evaluated in a Phase I, dose-escalation and dose expansion study to assess its safety, tolerability, and preliminary antitumor activity (ClinicalTrials.gov Identifier: NCT06004245). This study includes both monotherapy and combination therapy with pembrolizumab in patients with MSI and/or MMR-deficient (dMMR) advanced solid tumors.

Another WRN inhibitor with a disclosed structure currently undergoing Phase I clinical trials is **HRO761**, originally reported by Ferretti et al. (Figure 1).^22^ **HRO761** functions as a noncovalent allosteric WRN inhibitor and, similar to **VVD-214**, mirrors the phenotype observed by WRN genetic suppression. It has demonstrated tumor growth inhibition in both MSI cell-derived and patient-derived xenograft models following oral treatments.

**Figure 1:**
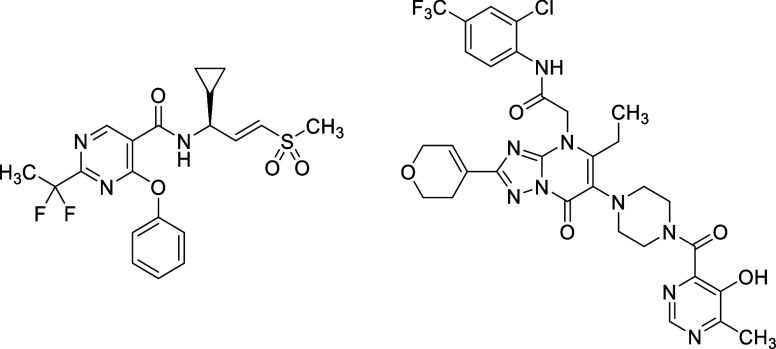
Structure of **VVD-214** (left) and **HRO761** (right).

In this article, we detail the medicinal chemistry endeavors that culminated in the identification of **VVD-214** from nucleotide-cooperative hits.^21^ Historically, helicases have proven difficult to drug, primarily due to the challenges associated with developing potent and selective compounds for this class of proteins, as described elsewhere.^23,24^ Our findings underscore a covalent allosteric approach for inhibiting WRN and support **VVD-214** as a promising drug candidate for patients with MSI-H cancers.

## Results and Discussion

In this study, we initially established the structure–activity relationship (SAR) of nucleotide cooperativity in a biochemical helicase assay, focusing on the core aromatic ring structure and its ortho substitution to the amide (Table 1). The ratio of helicase IC_50_ values when WRN is pretreated with or without ATP prior to initiation of the helicase reaction indicates the effect of binding of ATP (and ADP upon subsequent hydrolysis) to WRN on functional inhibition by allosteric binders. An IC_50_ ratio (−ATP/+ATP) less than unity suggests nucleotide-competitive behavior, while an IC_50_ ratio greater than unity implies nucleotide-cooperative behavior. Table 1 demonstrates that larger substitutions at the C2-position (R) on the benzoate (X=CH) reduce the nucleotide-competitive behavior of these inhibitors. This is evident in the comparison of compounds **1a–b** (IC_50_ ratio ≪1: nucleotide competitive) and compounds **1c–d** (IC_50_ ratio ∼1–3: nucleotide neutral). Some loss of nucleotide-competitiveness was also observed by changing the core structure from a benzene (X=CH) to a pyrimidine (X=N), as seen in the comparison of compounds **1b** and **1e**. Moreover, the combination of these two structural features led to strong nucleotide cooperativity (**1f**: IC_50_ ratio = 13). The significant impact of the phenoxy group on nucleotide cooperativity is consistent with the observation in the X-ray crystal structure of the **VVD-214**-bound WRN^21^ that this portion of the inhibitor indirectly interacts with the Walker A motif (Y575) in the nucleotide-binding site through Y849 (PDB code: 7GQU). These SAR observations provided valuable insights into designing nucleotide-cooperative WRN inhibitors, a crucial attribute for cellular activity due to the high ATP concentration within cells. As previously described, a strong correlation was observed between helicase assay potency when pretreated in the presence of ATP and half-maximal target engagement (TE_50_) potency in live cells.^21^

**Table 1:**
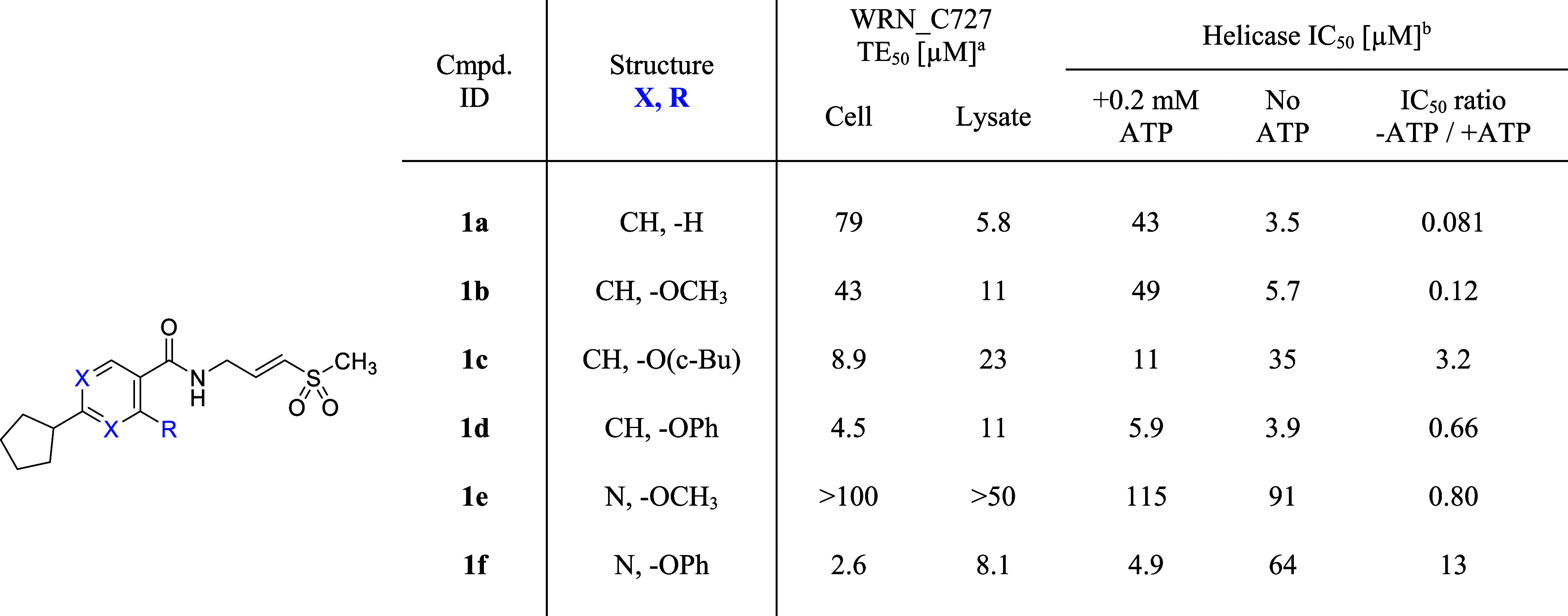
Exploration of Core Aromatic Rings and Their Substitutions toward the Nucleotide-Cooperative WRN Inhibitor

a OCI-AML2 cells. Cell and lysate TE_50_ values are determined at 2 h and 1 h time points, respectively. All TE_50_ data are the means of at least *n* = 2 independent measurements except for TE_50_ > 100 μM (*n* = 1). Each has a SEM ±0.2 log unit.

b Inhibition of DNA unwinding by hWRN^519–1227^. IC_50_ data are determined in a 30 min assay after a 30 min preincubation of the compound and WRN protein. Values shown are the means of at least *n* = 2 independent measurements. Each has a SEM ±0.2 log unit.

We then investigated the behavior of the vinyl sulfone warhead, an underrepresented electrophile in covalent chemistry (Table 2).^25,26^ The potential for chemical instability of α,β-unsaturated sulfones of the type represented by **1b–1f** has been documented, and we were particularly concerned about the potential for double bond migration, as it is known that the β,γ-unsaturated isomer should be thermodynamically more stable, owing both to the olefin destabilization by the strong electron-withdrawing group and little contribution of resonance stabilization between olefin and d-orbitals in the α,β-unsaturated isomer.^27^ Additionally, the intrinsic reactivity of *trans*-vinyl sulfone **1f**, measured by the glutathione consumption rate (GSH *k*_obs_/[*I*] of 0.038 M^–1^ s^–1^), was comparable to the aniline acrylamide-containing EGFR inhibitor osimertinib (GSH *k*_obs_/[*I*] = 0.037 M^–1^ s^–1^, in-house data). While this rate was acceptable, we generally strive for lower GSH rates, as seen in the piperidine acrylamide-containing BTK inhibitor ibrutinib (GSH *k*_obs_/[*I*] = 0.024 M^–1^ s^–1^, in-house data), to minimize potential undesirable covalent reaction in vivo, such as GSH conjugation and nonselective cysteine engagement. These observations prompted us to explore γ-substitution of the vinyl sulfone warhead to mitigate its reactivity. As anticipated, introducing a methyl group at the γ-position (R_1_ or R_2_=CH_3_, E) reduced the GSH consumption rate to approximately 0.02 M^–1^ s^–1^ (**2a–b**). This reduction is attributed to increased steric hindrance around the nucleophile’s trajectory, effectively shielding the electrophile. The γ-methyl group also hinders olefin transposition due to the lower kinetic acidity of the allylic proton, as aligning the σ bond of the allylic proton with the olefin’s π system requires higher energy in the presence of the γ-methyl group due to gauche interactions between the olefin and allylic substituents.^28^ Interestingly, despite the lower intrinsic reactivity, the γ-methyl group stereospecifically enhanced the potency of the compound; the *S* configuration (**2a**, R_1_=CH_3_) improved potency (helicase IC_50_ of 0.66 μM), while the *R* configuration (**2b**, R_2_=CH_3_) decreased potency (helicase IC_50_ of 30 μM). The improved potency of the *S* isomer **2a** over *R* isomer **2b** in the helicase assay was translated to a higher rate of target engagement for the former in live cells (TE_50_ at 2 h: 0.3 μM for **2a** vs 17 μM for **2b**). The observed stereopreference was subsequently rationalized through analysis of the cocrystal structure of **VVD-214** bound to WRN (PDB: 7GQU), which revealed a hydrophobic interaction between the γ-substituent and a hydrophobic patch formed by residues V570 and A706. Since an attempt to dock **2b** into the structure showed it is unable to exploit this interaction without significant structural rearrangement, we propose that the stereopreference is most likely reflective of a transition state, which provides optimal warhead orientation of **2a** for engagement of C727.

**Table 2:**
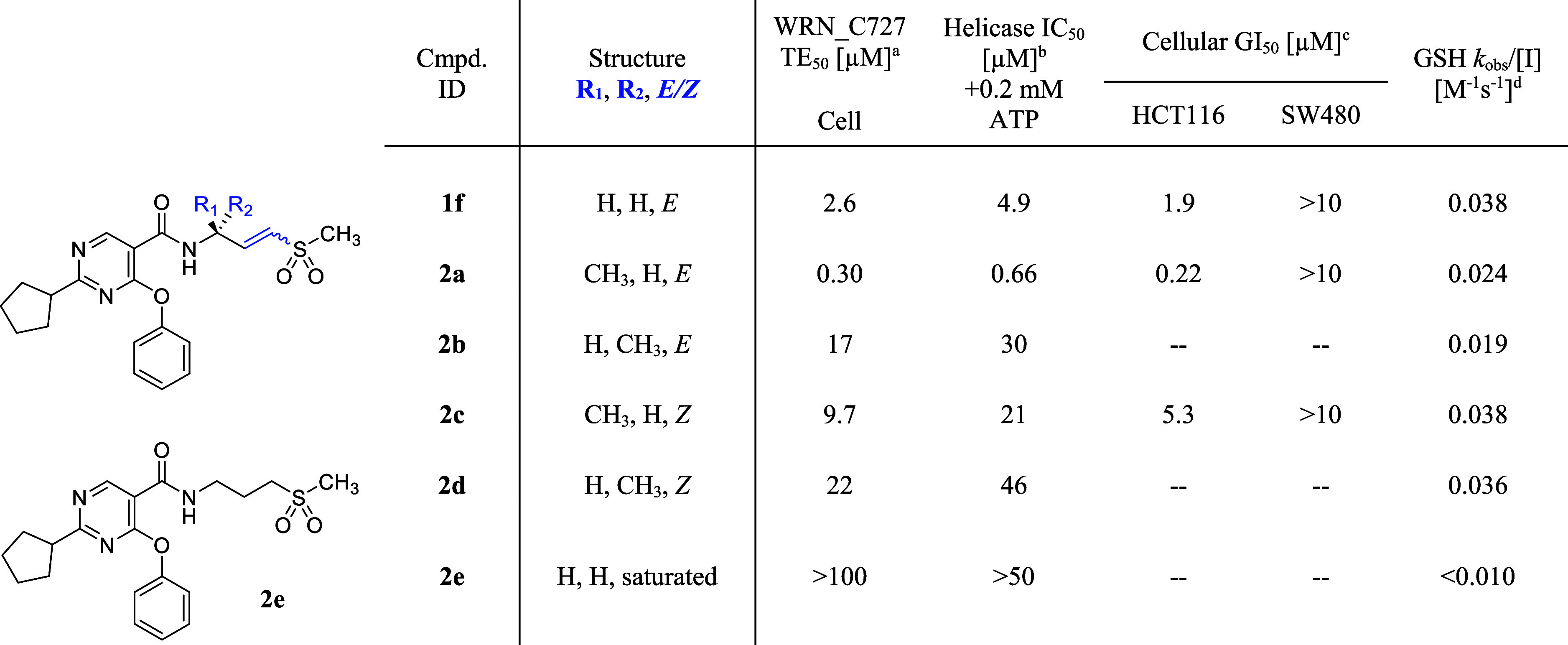
Exploration of γ-Substitutions and Olefin Geometries of the Vinyl Sulfone Warhead

a All cellular TE_50_ data are determined at 2 h, and the values shown are the means of at least *n* = 2 independent measurements except for TE_50_ > 100 μM (*n* = 1). Each has a SEM ±0.2 log unit.

b Inhibition of DNA unwinding by hWRN^519–1227^. IC_50_ data are determined in a 30 min assay after a 30 min preincubation of the compound and WRN protein. Values shown are the means of at least *n* = 2 independent measurements, except for IC_50_ > 50 μM (*n* = 1). Each has a SEM ±0.2 log unit.

c Growth inhibition of HCT116 (MSI-H) and SW480 (MSS) after 5 days of compound treatment. All GI_50_ data are the means of at least *n* = 2 independent measurements. Each has a SEM ±0.2 log unit.

d GSH consumption rate.

Next, we examined the influence of olefin geometry on the vinyl sulfone warhead. *cis*-Vinyl sulfones (**2c–d**) exhibited elevated intrinsic reactivity (GSH ∼0.04 M^–1^ s^–1^) compared to *trans*-vinyl sulfones (**2a–b**), likely due to olefin destabilization by steric strain between substituents of the *cis*-olefin. Despite the increased intrinsic reactivity of their warhead, these compounds proved to be weak inhibitors of WRN helicase. Furthermore, saturation of the warhead (**2e**) abrogated its potency. Collectively, these results suggest that the rate of target engagement (as reflected by single time point potency data) is likely driven by relatively weak reversible interactions that optimally position the covalent warhead for attack by C727, inducing a dramatic acceleration of the reaction, a conclusion eventually supported by biochemical examination of an advanced lead from these optimization efforts (vide infra). These findings also highlight the highly tunable nature of the vinyl sulfone warhead and its utility for covalent chemistry.

The potent helicase inhibitor **2a** demonstrated selective cellular growth inhibition in the MSI-H cell line HCT116 (GI_50_ = 0.22 μM), compared to the MSS cell line SW480 (GI_50_ > 10 μM), consistent with the synthetic lethality of WRN inhibition in MSI-H cancer cells. GI_50_ observed in HCT116 aligned with both cellular TE_50_ and helicase IC_50_.

Subsequent investigation into alternative heteroaromatic rings for the core structure revealed a compelling SAR that underscored the influence of warhead orientation on helicase potency (Table 3). Two pyridine isomers **3a–b** exhibited helicase IC_50_ of 1.0 and 2.1 μM, respectively, comparable to pyrimidine **2a**. In contrast, the other pyridine isomer **3c**, along with pyrazine **3d** and pyridazine **3e** displayed a marked reduction in potency with helicase IC_50_ values of 17, 9.9, and >50 μM, respectively.

**Table 3:**
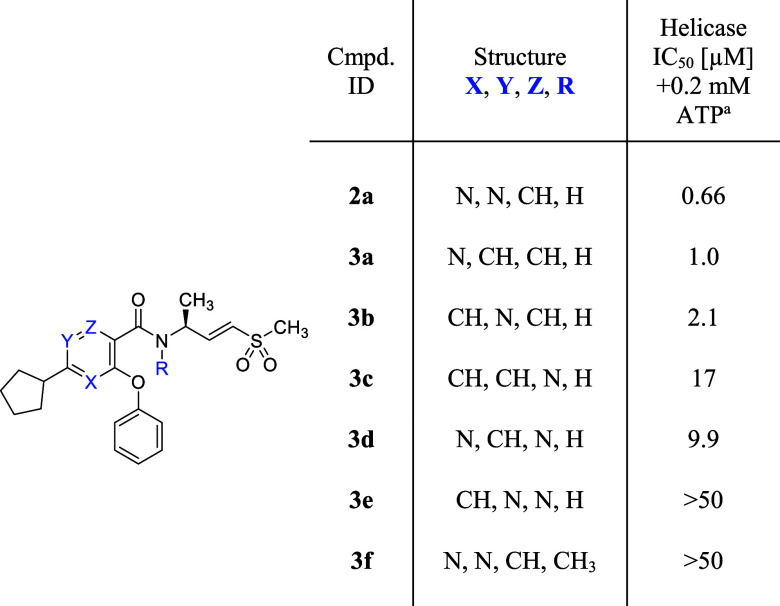
Exploration of the Alternative Heteroaromatic Core

a Inhibition of DNA unwinding by hWRN^519–1227^. IC_50_ data are determined in a 30 min assay after a 30 min preincubation of the compound and WRN protein. Values shown are the means of at least *n* = 2 independent measurements, except for IC_50_ > 50 μM (*n* = 1). Each has a SEM ±0.2 log unit.

To elucidate the SAR for the above observation, the impact of the core structure on compound conformation, specifically the orientation of the warhead relative to the core, was evaluated through a dihedral scan around the carbon–carbon bond to the amide (Figure 2a). Intriguingly, potent compounds **2a** and **3a–b** (Z=CH) maintained coplanarity of their amide group with the core ring (energy minimum at 0 ∼ 30°), with the hydrogen of the amide group oriented toward the ether oxygen (Figure 2b). Meanwhile, weak or inactive inhibitors **3c–e** (Z=N) prefer a conformation with the warhead orientation inverted (energy minimum at around 180°), favoring the hydrogen of the amide to orient toward the aromatic nitrogen (Figure 2c).

**Figure 2:**
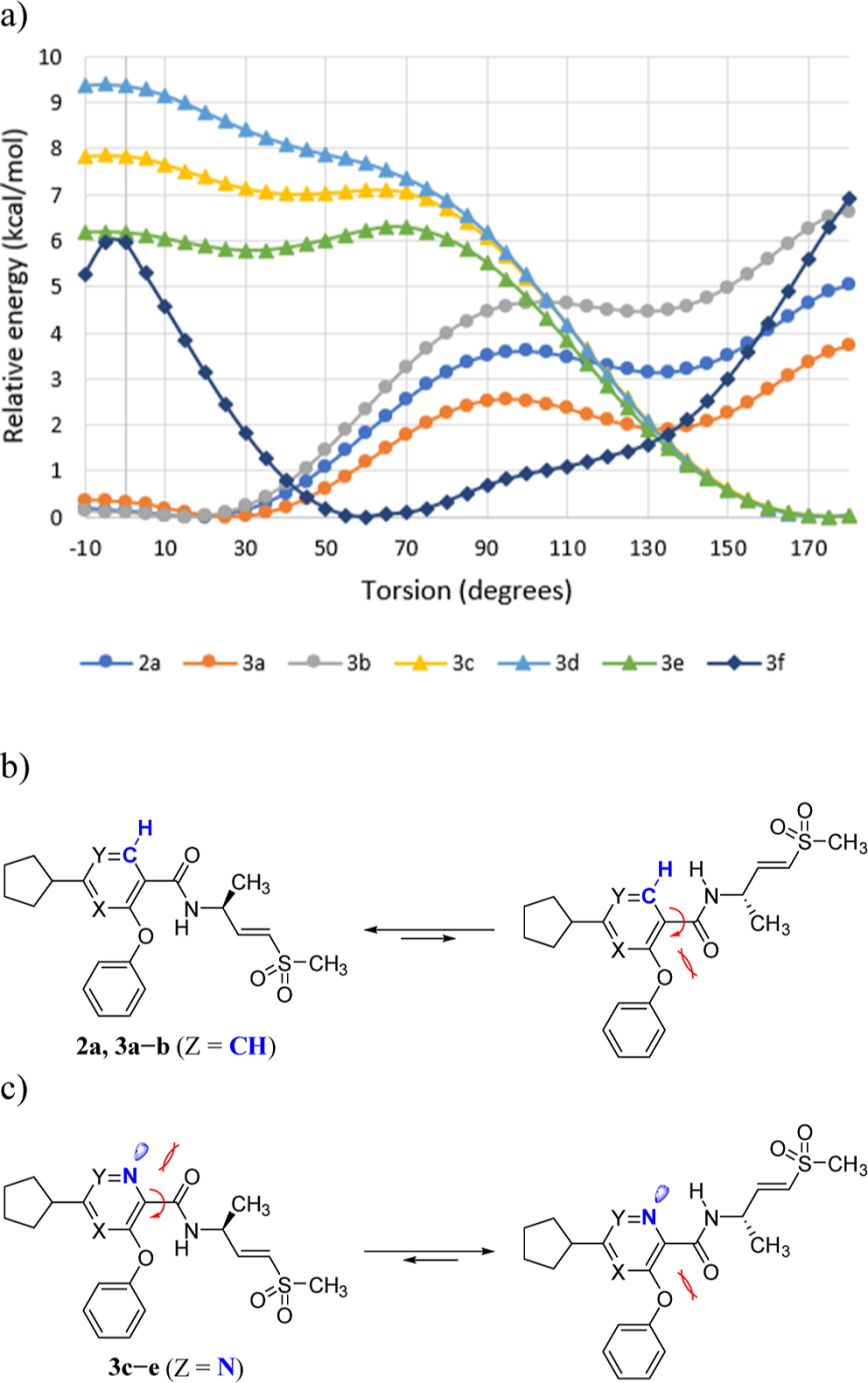
(a) Dihedral scan performed for the C–C bond to the amide group of **2a** and **3a–f** without vinyl sulfone (corresponding methyl amide) using AMBER:EHT force field-based torsion scan as implemented in Molecular Operating Environment (MOE, Chemical Computing Group, Montreal, Canada). Preferred orientation of the warhead for (b) **2a** and **3a–b** (Z=CH) and (c) **3c–e** (Z=N).

Despite their apparent proximity, the hydrogen of the amide did not form strong intramolecular hydrogen bonds (IMHBs) with the ether oxygen or the aromatic nitrogen in these compounds, as indicated by the borderline *A*_NMR_ values of 0.09 ∼ 0.16, higher than the criteria of <0.05 for strong IMHBs (Table S1).^29^ For compounds **2a** and **3a–b** (Z=CH), the alternative coplanar conformation was disfavored, presumably due to lone-pair repulsion between the carbonyl group and the ether oxygen. For compounds **3c–e** (Z=N), the conformational preference could be attributed to the stronger lone-pair repulsion between the amide carbonyl group and the aromatic nitrogen compared to that of the ether oxygen. Finally, methylation of the amide nitrogen (R=CH_3_: **3f**), which induced a twist out of planarity (energy minimum at around 60°) due to steric clash, resulted in a significant decrease in potency. The relationship of potency to the orientation of the covalent reactive group is consistent with a recognition event between the target protein and ligand, which most likely favors the shared low energy conformations of pyrimidine **2a** and pyridines **3a–b**.

An attempt to optimize the ether group portion revealed that the unsubstituted phenyl ether **2a** was the most potent and selective for both helicase inhibition and cellular growth inhibition (Tables 4 and S2). Replacing the phenyl ether of **2a** with cyclohexyl ether (**4a**) retained potency in helicase inhibition and HCT116 (MSI-H) growth inhibition but also inhibited growth in SW480 cells (MSS) with an GI_50_ of 4.9 μM. The incorporation of 4-tetrahydropyranyl ether (**4b**) was not tolerated, showing helicase IC_50_ of 31 μM. Substituting the phenyl ether with chlorine, fluorine, or methyl group resulted in a reduction in potency (**4c–g**), with the best helicase IC_50_ of 1.6 μM for meta-fluoride **4f**. This SAR suggested a small and lipophilic pocket for the phenyl ether, which was later confirmed by the cocrystal structure of **VVD-214** bound to WRN.^21^

**Table 4:**
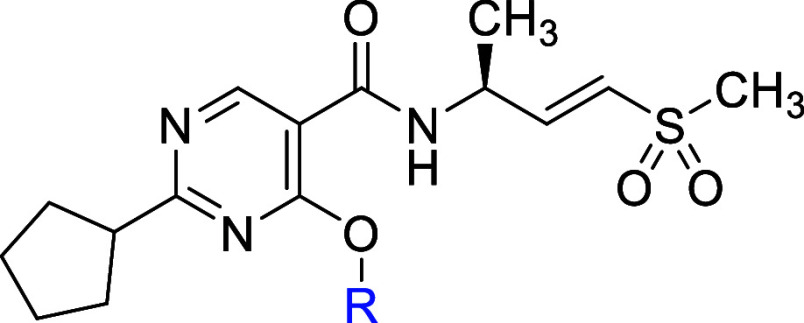

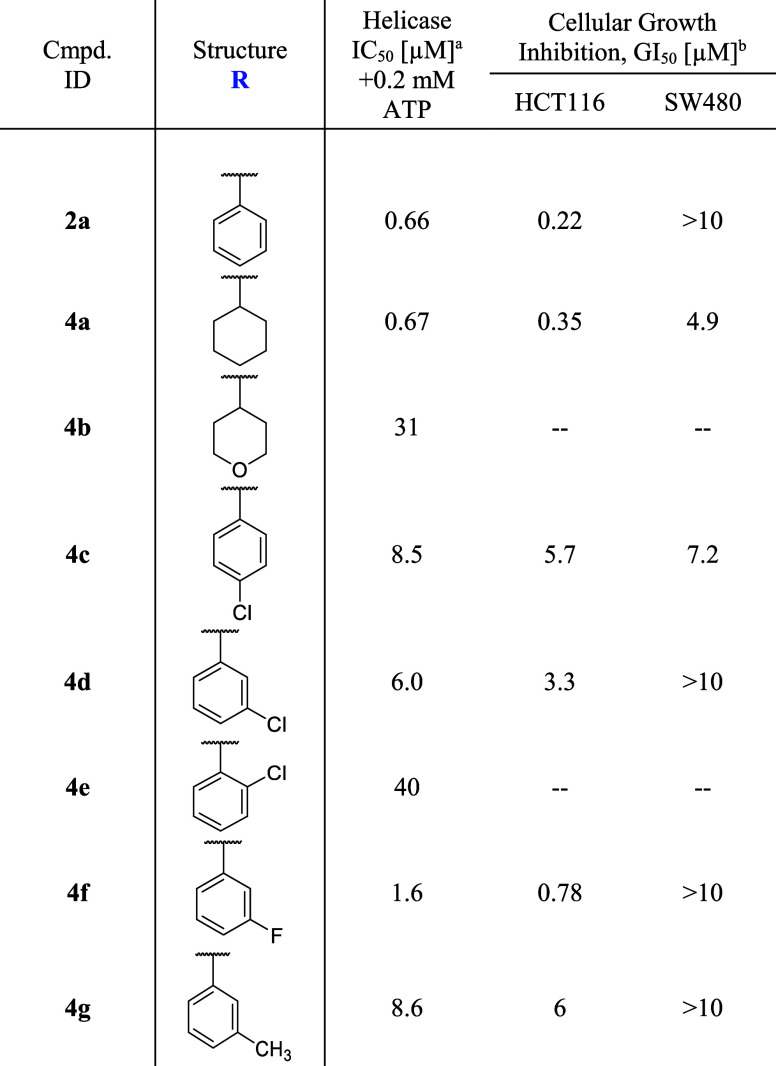
Pyrimidine Ether SAR

a Inhibition of DNA unwinding by hWRN^519–1227^. IC_50_ data are determined in a 30 min assay after a 30 min preincubation of the compound and WRN protein. Values shown are the means of at least *n* = 2 independent measurements. Each has a SEM ±0.2 log unit.

b Growth inhibition of HCT116 (MSI-H) and SW480 (MSS) after 5 days of compound treatment. All GI_50_ data are the means of at least *n* = 2 independent measurements. Each has a SEM ±0.2 log unit.

To enhance the potency of the inhibitors, the alkyl group at the C2-position of the pyrimidine core was examined (Table 5). This SAR exploration revealed a trend of potency increase dependent on the size and lipophilicity of the C2-alkyl group, as observed in compounds **5a–d**. The measured LogD_7.4_ increased from the methyl (**5a**, 0.76), to ethyl (**5b**, 1.38), to isopropyl (**5c**, 2.04), to *tert*-butyl compound (**5d**, 2.80), while helicase potency improved from an IC_50_ of 87 μM, to 6.2 μM, to 0.94 μM, to 0.24 μM, respectively. Predictably, this SAR trend exhibited a lack of enhancement in lipophilic ligand efficiency (LipE) and an increase in hepatocyte unbound clearance (CL_int,u_) due to increased lipophilicity.^30^

**Table 5:**
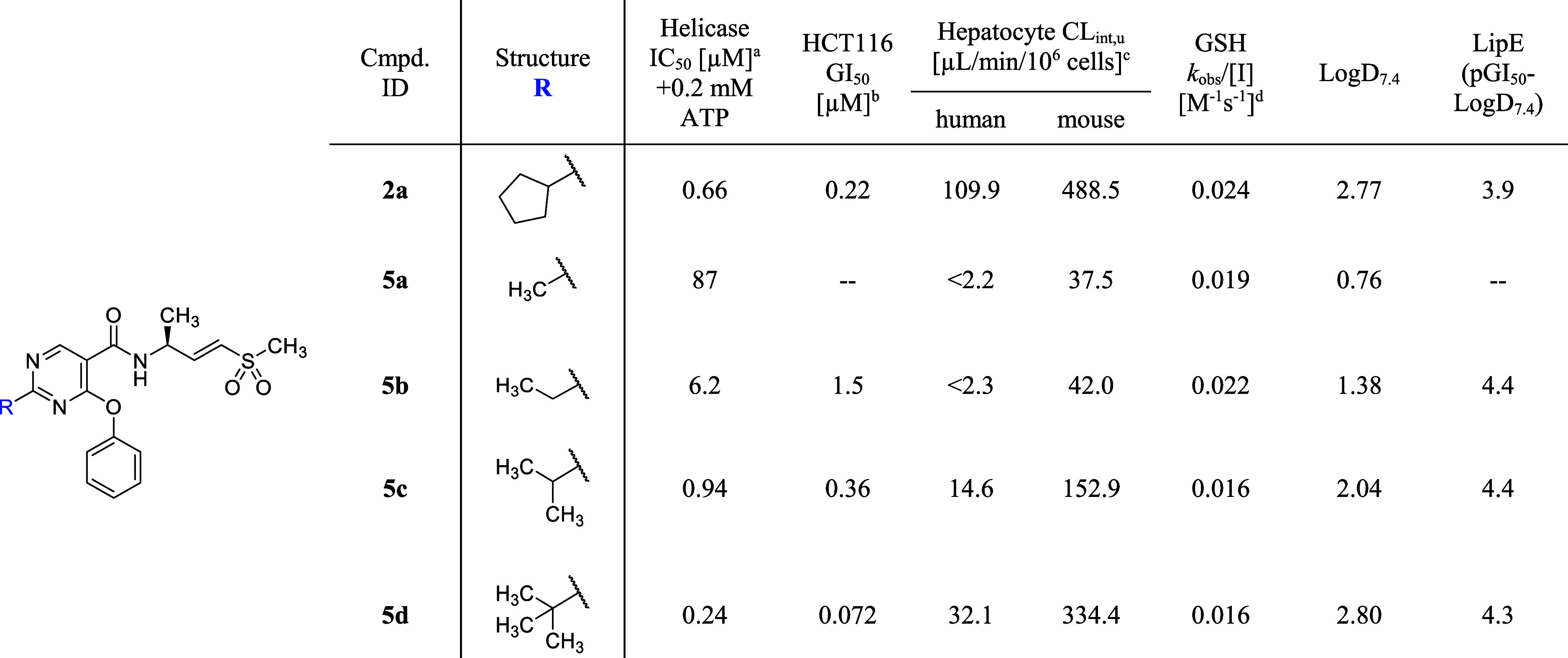
Evaluation of C2-Position of the Pyrimidine Core

a Inhibition of DNA unwinding by hWRN^519–1227^. IC_50_ data are determined in a 30 min assay after a 30 min preincubation of the compound and WRN protein. Values shown are the means of at least *n* = 2 independent measurements. Each has a SEM ±0.2 log unit.

b Unbound intrinsic clearance (CL_int,u_) is calculated using CL_int,u_ = CL_int_/fu_inc_, where fu_inc_ (incubation binding) is estimated using the Austin model.^31^.

c Growth inhibition of HCT116 (MSI-H) after 5 days of compound treatment. All GI_50_ data are the means of at least *n* = 2 independent measurements. Each has a SEM ±0.2 log unit.

d GSH consumption rate.

Despite this unfavorable trend, compound **5d** demonstrated meaningful potency improvements over compound **2a**, providing the first compound with a GI_50_ under 100 nM in HCT116 cells. It also achieved unbound subcutaneous exposure comparable to compound **2a** in mice at 30 mg/kg (*C*_max,u_ and AUC_0‑last,u_, Table 6). Consequently, we selected compound **5d** as an in vivo tool to evaluate the correlation between plasma exposure and target engagement.

**Table 6:** Subcutaneous PK Parameters for Compound **2a** and **5d** in Mice^a^

PK parameters^b^, SC	**2a**	**5d**
mouse PPB [%]	93.2	96.4
*C*_max_ (*C*_max,u_) [ng/mL]	5323 (362)	11220 (404)
AUC_0‑last_ (AUC_0‑last,u_) [hr·ng/mL]	5102 (347)	10098 (364)
*T*_max_ [hr]	0.417	0.417

a Male CD-1 Mouse, *n* = 3, 30 mg/kg SC, vehicle: 5% DMSO/95% (20%(2-hydroxypropyl)-beta-cyclodextrin in water).

b PPB, plasma protein binding; *C*_max_, maximal plasma concentration; AUC_0‑last_, area under the plasma concentration–time curve from 0 to the last measured time point; *T*_max_, time at which *C*_max_ is attained.

Compound **5d** was administered subcutaneously to mice (athymic nude, female) bearing the HCT116 xenograft at doses of 10, 30, and 100 mg/kg (Figure 3). Measurements at 0.5, 2, and 24 h demonstrated a dose-dependent increase in plasma exposure. The extent of tumor TE at WRN C727 also showed a dose-dependent increase at 2 h postdose (TE_2h_ = 47%, 82%, and 90% for 10, 30, and 100 mg/kg, respectively). Interestingly, at 24 h postdose, despite negligible plasma exposure of less than 10 ng/mL, a significant level of TE was maintained across all dose levels (TE_24h_ = 26%, 44%, and 57% for 10, 30, and 100 mg/kg, respectively). This phenomenon of prolonged TE can be explained by the irreversible covalent nature of the compound, where the rate of TE loss is solely controlled by the protein turnover rate in the absence of free compound (the measured in vitro half-life of WRN is 13 h).^21^

**Figure 3:**
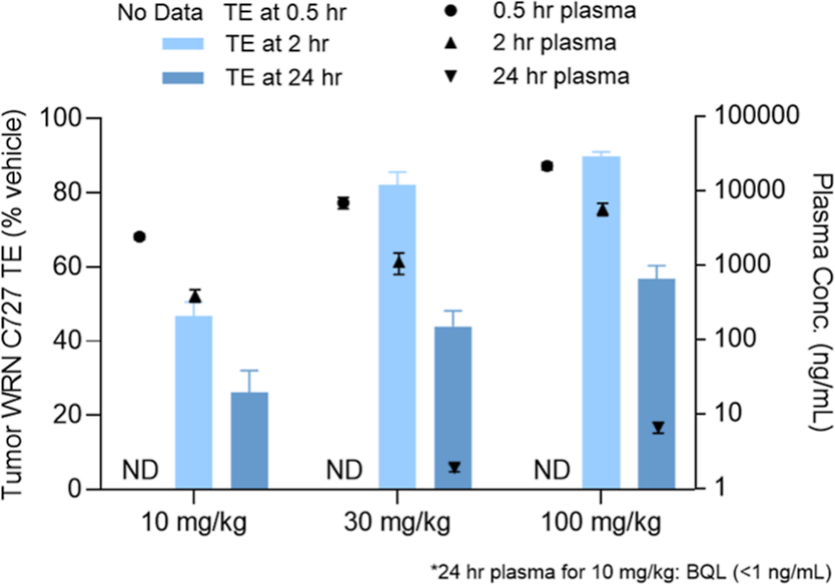
PK/TE of compound **5d** via acute subcutaneous administration in HCT116 xenograft mice (female, *n* = 4). Data shown as means with SEM.

Following the robust single-dose PK/TE results, we assessed the antitumor activity of compound **5d** in the HCT116 xenograft mouse model (Figure 4). The compound was administered subcutaneously at doses of 10, 30, and 100 mg/kg once daily for 3 weeks. Treatment with 100 mg/kg resulted in tumor stasis (tumor growth inhibition (TGI) = 98%), while treatments with 30 mg/kg and 10 mg/kg resulted in moderate (TGI = 50%) and low (TGI = 23%) responses, respectively. The results marked our first in vivo proof-of-concept, demonstrating the antitumor efficacy of the WRN inhibitor in an MSI-H xenograft mouse model.

**Figure 4:**
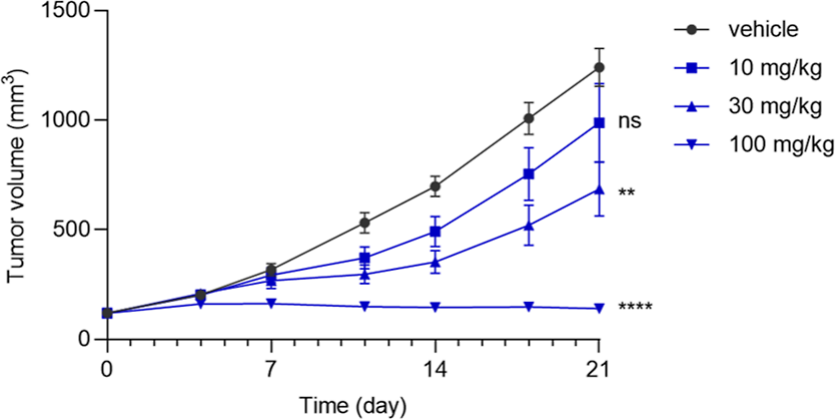
Antitumor efficacy of compound **5d** via subcutaneous administration in HCT116 xenograft mouse model (female, *n* = 5 for 10 and 30 mg/kg, *n* = 10 for 100 mg/kg). Data shown as means with SEM and analyzed by one-way ANOVA, **P < 0.01, ****P < 0.0001.

To achieve more profound efficacy (i.e., tumor regression) in the xenograft mouse model, it was anticipated that higher and more sustained tumor TE over 24 h would be required. Toward that end, a campaign to optimize potency and metabolic stability with the aim of achieving a low efficacious oral dose was initiated. Further exploration of the C2-position of the pyrimidine core determined that fluorination of the benzylic position significantly impacted both hepatocyte stability and potency (Table 7). Due to its unique properties that enable both conformational control and electronic modulation, fluorine has been widely utilized in drug design and development.^32,33^ These properties influence critical compound properties, including absorption, distribution, metabolism, and excretion (ADME). Monofluorination of the cyclopentyl group (**6a**) and the isopropyl group (**6d**) resulted in a reduction of LogD_7.4_ by approximately 0.5 units, leading to improved unbound clearance in both human and mouse hepatocytes relative to their nonfluorinated counterparts, compounds **2a** and **5c**, respectively. This modification had a minor impact on helicase and cellular potency, with the shift in IC_50_ and GI_50_ being around 2-fold or less.

**Table 7:**
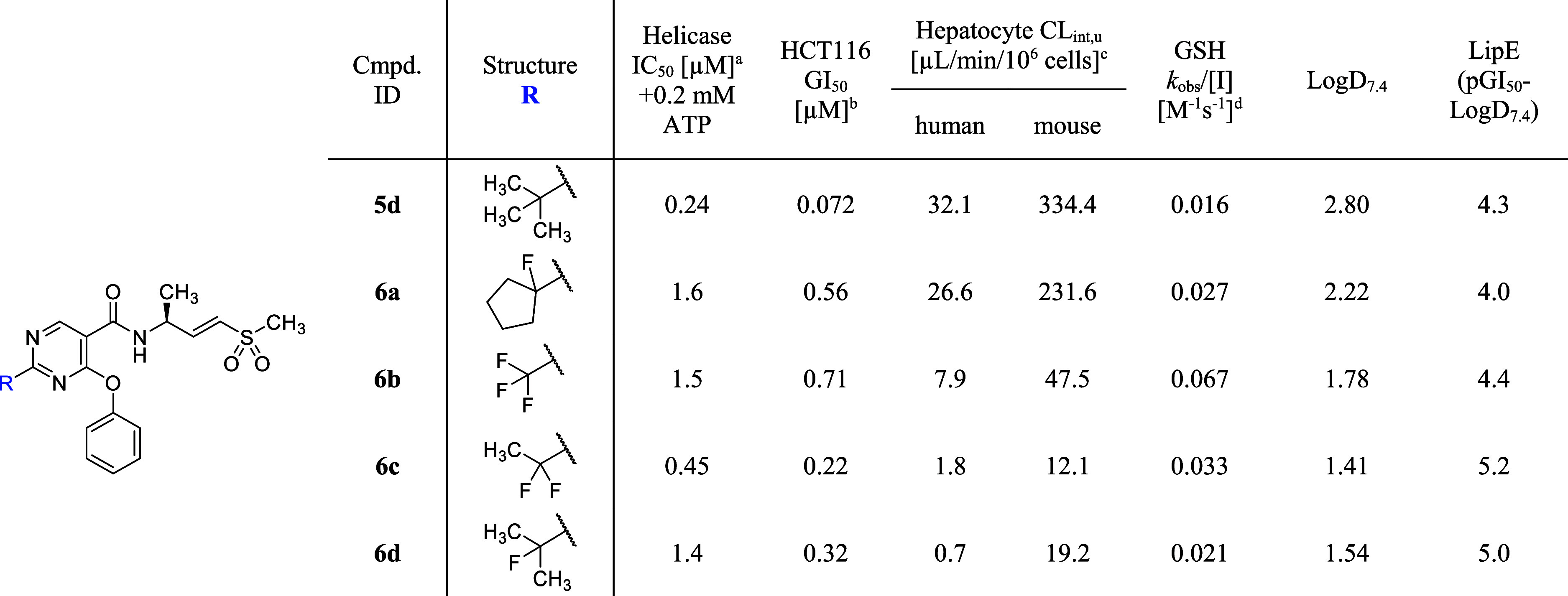
Fluorinated Alkyl Groups for C2-Substitution of the Pyrimidine Core

a Inhibition of DNA unwinding by hWRN^519–1227^. IC_50_ data are determined in a 30 min assay after a 30 min preincubation of the compound and WRN protein. Values shown are the means of at least *n* = 2 independent measurements. Each has a SEM ±0.2 log unit.

b Growth inhibition of HCT116 (MSI-H) after 5 days of compound treatment. All GI_50_ data are the means of at least *n* = 2 independent measurements. Each has a SEM ±0.2 log unit.

c Unbound intrinsic clearance (CL_int,u_) is calculated using CL_int,u_ = CL_int_/fu_inc_, where fu_inc_ (incubation binding) is estimated using the Austin model^31^.

d GSH consumption rate.

Contrastingly, 2-trifluoromethyl pyrimidine **6b** exhibited a significant improvement in helicase potency over 2-methyl pyrimidine **5a**, with a greater than 50-fold shift in IC_50_. Similarly, difluorination of the ethyl group (**6c**) led to a more potent inhibitor compared to 2-ethyl pyrimidine **5b** (over 10-fold IC_50_ improvement), while maintaining satisfactory hepatocyte stability. This observation was consistent with a meaningful improvement in LipE. The observed potency improvement by polyfluorination was partially attributed to the enhanced warhead reactivity, evidenced by the 3.5- and 1.5-fold increase in GSH consumption rate for compounds **6b** and **6c**, respectively. This was presumably caused by the strong inductive effect of polyfluorinated alkyl groups, despite the site of fluorination being considerably distant from the Michael acceptor. The effects of subtle electronic changes on the intrinsic reactivity of the covalent reactive group underscore the need for continual monitoring of warhead reactivity throughout the covalent drug discovery process to identify any unforeseen changes in reactivity that have the potential to compromise selectivity and, ultimately, safety.^34^

Following a significant improvement in hepatocyte stability, the PK of compound **6c** was evaluated in mice alongside key SAR compounds **2a** and **5d** (Table 8). Upon intravenous (IV) administration at a 1 mg/kg dose, compound **6c** exhibited low clearance of 24 mL/min/kg, equivalent to 25% of liver blood flow, and a moderate volume of distribution. After an oral (PO) dose of 10 mg/kg, it demonstrated moderate bioavailability (F) of 45% and high unbound plasma exposure, with a *C*_max,u_ of 595 ng/mL and AUC_0‑last,u_ of 577 h·ng/mL. These findings represent substantial improvements over compounds **2a** and **5d** in terms of IV clearance, oral unbound plasma exposure, and bioavailability. Notably, these compounds exhibited a robust in vitro/in vivo correlation for clearance, with the estimated hepatic clearance (CL_hep,estimate_) being consistent with the measured IV clearance (within 2-fold). This suggests minimal extrahepatic metabolism of the compounds, aligning with their satisfactory whole blood stability in mice.^35^

**Table 8:** Single-Dose IV and PO Mean PK Parameters of Compound **2a**, **5d,** and **6c** ^a^

parameters	**2a**	**5d**	**6c**
mouse whole blood [% remaining at 1h]	85	92	95
mouse PPB [%]	93.2	96.4	81.7
CL_hep,estimate_ [mL/min/kg]^b^	68	45	13
CL [mL/min/kg]	70	35	24
*V*_d,ss_ [L/kg]	0.63	0.38	0.65
PO *C*_max_ (*C*_max,u_) [ng/mL]	45 (3.1)	1158 (42)	3251 (595)
PO AUC_0‑last_ (AUC_0‑last,u_) [hr·ng/mL]	34 (2.3)	808 (29)	3151 (577)
F [%]	1.4	18	45

a Male CD-1 Mouse, *n* = 3, 1 mg/kg IV and 10 m/kg PO, vehicle: 5%DMSO/95%(20%(2-hydroxypropyl)-beta-cyclodextrin in water).

b CL_hep,estimate_ = *Q*_h_ × *f*_u,p_ × CL_int_liver,u_/(*Q*_h_ + *f*_u,p_ × CL_int_liver,u_) where CL_int_liver,u_ = CL_int,u_ × (hepatocyte scaling factor) × (liver weight).

Although we successfully achieved satisfactory oral PK in mice by optimizing the C2-pyrimidine substituent through systematic fluorination of the alkyl group, the potencies in helicase and growth inhibition assays were compromised. Therefore, further enhancement of the inhibitor potency was deemed necessary to attain higher target engagement and resultant efficacy for TGI after oral administration. Additionally, compound **6c** exhibited elevated intrinsic reactivity of the warhead, a characteristic that could potentially undermine global cysteine selectivity.

Gratifyingly, we identified a solution to these challenges by further tuning the vinyl sulfone warhead (Table 9). Replacing the γ-methyl group of the vinyl sulfone with a γ-cyclopropyl group (**VVD-214**) enhanced potency, improving helicase IC_50_ from 0.45 μM to 0.13 μM and HCT116 GI_50_ from 0.22 μM to 0.043 μM, while maintaining stability in hepatocytes and whole blood. Suboptimal human whole blood stability suggests the potential for extrahepatic clearance in humans, which contrasts with the mouse profile. Cyclobutyl analog **7a** exhibited a similar trend, albeit to a lesser extent. Notably, these larger substituents decreased the intrinsic reactivity of the warhead. Modifying γ-substitution with ether groups (**7b–c**) also improved potency, particularly in the growth inhibition assay. However, for compound **7c**, this enhancement was accompanied by a significant increase in warhead reactivity due to the strong inductive effect of the difluoro-methoxy methyl group, resulting in decreased stability in hepatocyte and whole blood. Combining the γ-cyclopropyl group with a metabolically stable C2-fluoroisopropyl pyrimidine core resulted in compound **7d**. This compound demonstrated a substantial improvement in potency over compound **6d**, with a 10-fold increase in helicase IC_50_ and a 4-fold increase in HCT116 GI_50_, while maintaining low intrinsic reactivity of the warhead and preferable metabolic stability. These results further illustrate the tunability of the vinyl sulfone warhead, supporting its potential for broad utility in covalent drug discovery.

**Table 9:**
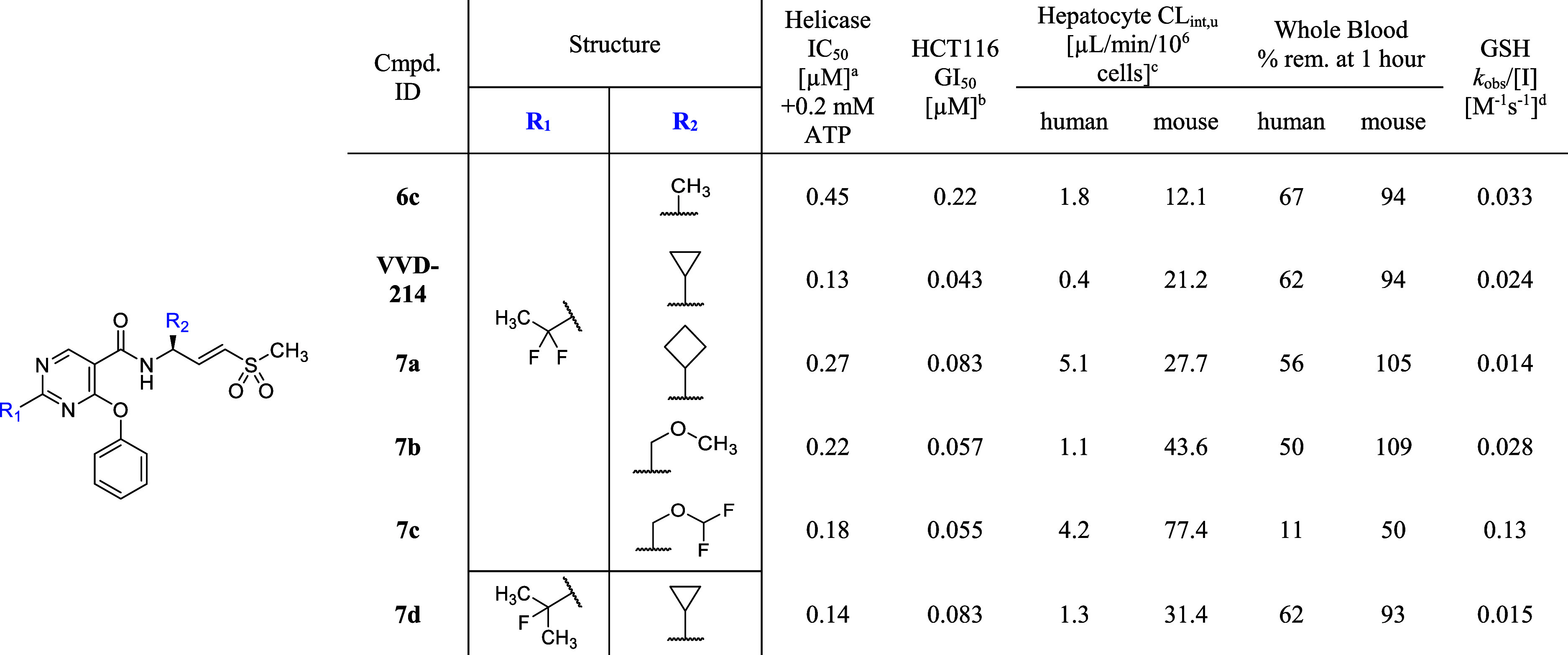
Optimization of γ-Substitution of the Vinyl Sulfone Warhead

a Inhibition of DNA unwinding by hWRN^519–1227^. IC_50_ data are determined in a 30 min assay after a 30 min preincubation of the compound and WRN protein. Values shown are the means of at least *n* = 2 independent measurements. Each has a SEM ±0.2 log unit.

b Growth inhibition of HCT116 (MSI-H) after 5 days of compound treatment. All GI_50_ data are the means of at least *n* = 2 independent measurements. Each has a SEM ±0.2 log unit.

c Unbound intrinsic clearance (CL_int,u_) is calculated using CL_int,u_ = CL_int_/fu_inc_, where fu_inc_ (incubation binding) is estimated by the Austin model^31^.

d GSH consumption rate.

With multiple analogs demonstrating favorable potency and metabolic stability profiles, a few select compounds were administered to HCT116 xenograft tumor-bearing mice to determine tumor target engagement at 24 h after a single dose (tumor TE_24h_) and identify the best candidate for further profiling (Figure 5). Following a single 100 mg/kg oral dose, **VVD-214** exhibited superior performance with 92% tumor TE_24h_, compared to 78% for compound **7b** and 53% for compound **7d**. These results are consistent with **VVD-214**’s superior potency and metabolic stability among the three compounds. Subsequent dose-down experiments confirmed **VVD-214’s** outstanding potential; a 30 mg/kg oral dose achieved 81% TE_24h_ and a 10 mg/kg oral dose resulted in 67% TE_24h_, both surpassing the 57% tumor TE_24h_ achieved by 100 mg/kg subcutaneous dose of compound **5d**, which resulted in tumor stasis upon repeated dosing in TGI study, as described earlier.

**Figure 5:**
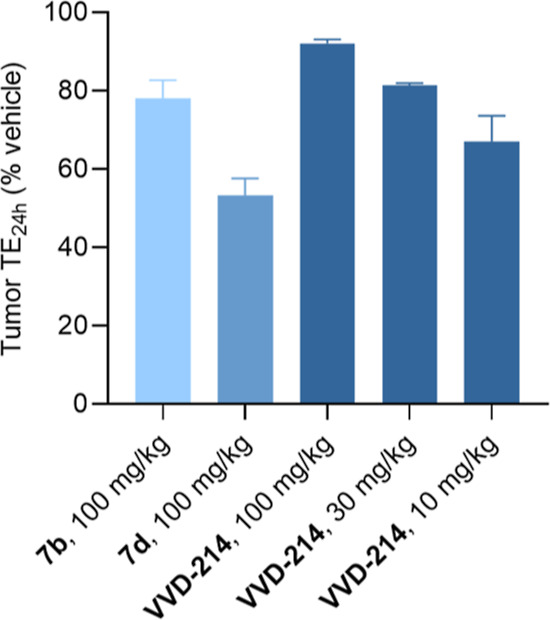
Tumor TE on WRN C727 at 24 h (tumor TE_24h_) post single oral dose of compounds **7b**, **7d**, and **VVD-214** in HCT116 xenograft mice (female, *n* = 3 for **7b**, **7d,** and *n* = 4 for **VVD-214**). Data shown as means with SEM.

Based on the results of the single-dose TE study for **VVD-214**, a 10 mg/kg once-daily treatment was anticipated to achieve profound efficacy in the HCT116 xenograft mouse model. Prior to initiating the TGI study with **VVD-214**, a repeat-dose TE study was conducted to examine the effect on tumor TE_24h_ compared to a single dose, as well as to further refine the dose selection for the TGI study. HCT116 xenograft tumor-bearing mice were administered oral doses of 40, 20, 10, and 5 mg/kg once daily for 3 days, and tumor TE on WRN C727 was measured 24 h after the last dose (Figure 6a). TE_24h_ from this study at a 10 mg/kg dose was 63%, comparable to the TE_24h_ of 67% observed after a single dose at 10 mg/kg, indicating neither accumulation of TE nor loss of plasma exposure upon repeat dosing. Additionally, the 40 mg/kg dose displayed comparable TE_24h_ with the 20 mg/kg dose (82% and 80%, respectively), leading to the selection of 20 mg/kg as the top dose for the TGI study, along with 10, 5, and 2.5 mg/kg to identify the efficacious dose. As detailed in our earlier report, significant antitumor activity was observed in the HCT116 xenograft mouse model, with the treatment groups receiving 20, 10, 5, and 2.5 mg/kg once daily for 3 weeks, demonstrating TGI of 106%, 105%, 93%, and 56%, respectively.^21^ Plotting TGI responses against tumor TE_24h_ for **VVD-214** (24 h after the last dose of once-daily treatment for 3 days) and compound **5d** (24 h after a single dose) showed a correlation between tumor TE_24h_ and TGI responses across compounds and doses (Figure 6b). Overall, a general observation emerged that trough TE (TE_24h_ for once-daily dosing) in the tumor is highly correlated to the extent of TGI responses, an observation that is consistent with results from other covalent drug discovery programs directed toward oncology targets (in-house data, not shown). This is advantageous as the selection of compounds and doses for labor-intensive TGI studies can be guided by short-term TE experiments, measuring trough TE in the tumor, as demonstrated in the identification of **VVD-214**. Given the superior in vivo efficacy data, **VVD-214** was advanced for further profiling.

**Figure 6:**
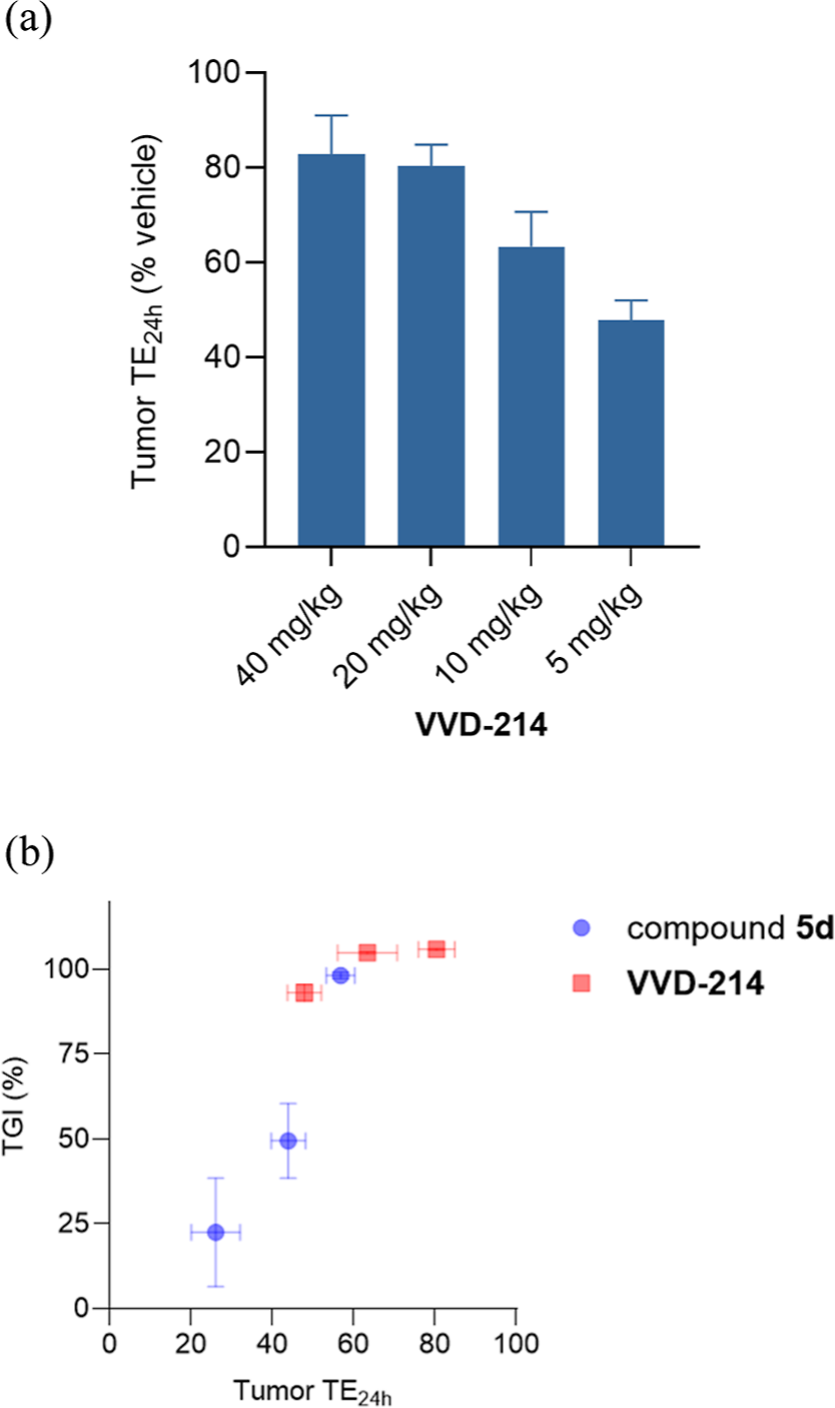
(a) Tumor TE on WRN C727 at 24 h (tumor TE_24h_) after the last dose of **VVD-214** once-daily oral treatment for 3 days in HCT116 xenograft mice (female, *n* = 4). Data shown as means with SEM (b) TGI (%) vs Tumor TE_24h_ for compound **5d** (TE_24h_ measured after single SC dose of 100, 30, and 10 mg/kg) and **VVD-214** (TE_24h_ measured after 3 day once-daily PO treatment of 20, 10, and 5 mg/kg).

The pharmacokinetics of **VVD-214** were evaluated across four species: mouse, rat, dog, and monkey, and the key parameters are summarized in Table 10. These parameters were obtained from PK studies involving IV dosing at 1 mg/kg and PO dosing at 10 mg/kg for all species. The oral bioavailability observed across these species was moderate to high, with *F* values ranging from 49% to 108%, suggesting the potential for similar oral bioavailability in human studies. The IV clearance was low to moderate, accounting for 21% to 55% of hepatic blood flow. The volume of distribution was moderate, and the oral exposure, measured as AUC_0‑last_, was moderate to high. Overall, these findings indicate a favorable pharmacokinetic profile for **VVD-214**.

**Table 10:** Single-Dose IV and PO Mean PK Parameters of VVD-214^a^

PK parameters	mouse	rat	dog	monkey
CL [mL/min/kg]	27	37	6.5	10
*V*_d,ss_ [L/kg]	0.7	0.9	0.7	0.8
PO AUC_0‑last_ [hr·ng/mL]	4408	2228	23542	17532
*F* [%]	72	49	91	108

a Male CD-1 mouse, male SD rat, male beagle dog, female cynomolgus monkey, *n* = 3, 1 mg/kg IV and 10 m/kg PO, vehicle: 10%DMSO/30%PEG400/60%(20%(2-hydroxypropyl)-beta-cyclodextrin in water).

Throughout the optimization of C2-substitution of the pyrimidine core and γ-substitution on the vinyl sulfone warhead, remarkable enhancement in reaction kinetics was steadily achieved without increasing the intrinsic reactivity of the warhead. This is particularly evident when comparing reaction rates determined by intact protein mass spectrometry for compound **1f** and **VVD-214** (Table 11), where **VVD-214** exhibited a significant improvement in the second-order rate constant for covalent inactivation of WRN (*k*_inact_/*K*_I_), of approximately 50-fold, despite a reduction in the intrinsic warhead reactivity. Intriguingly, even with this dramatic potency improvement, the noncovalent affinity remained very weak, as evidenced by the absence of helicase potency in the corresponding saturated analogs (compounds **2e** and **8a**). This observation is consistent with the findings from the X-ray crystal structure (PDB code: 7GQU), which shows that covalently engaged **VVD-214** makes only weak hydrophobic interactions with surrounding residues. Additionally, the individual *k*_inact_ and *K*_I_ values eluded determination because the observed reaction rates remained linear up to the highest concentrations tested (*k*_obs_ = *k*_inact_ [*I*]/(*K*_I_ + [*I*]) ∼ (*k*_inact_/*K*_I_)­[*I*] when *K*_I_ ≫ [*I*]) (Figure S14). These analyses led us to hypothesize that the improvement of binding kinetics for **VVD-214** was achieved through an overall optimization of the reaction (i.e., *k*_inact_/*K*_I_), presumably facilitated by optimal warhead alignment for covalent engagement of C727 by weak reversible interactions.^36,37^

**Table 11:**
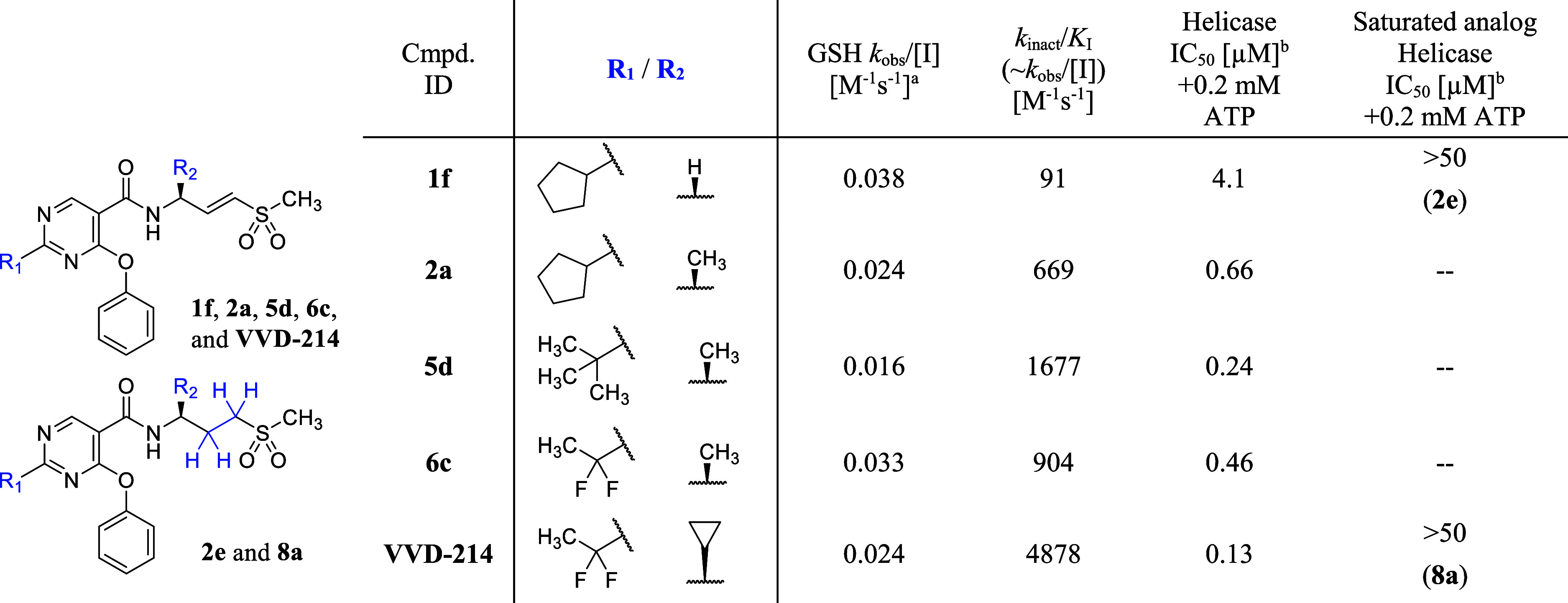
Binding Kinetics of Compounds **1f**, **2a**, **5d**, **6c**, and VVD-214

a GSH consumption rate.

b Inhibition of DNA unwinding by hWRN^519–1227^. IC_50_ data are determined in a 30 min assay after a 30 min preincubation of the compound and WRN protein. Values shown are the means of at least *n* = 2 independent measurements except for IC_50_ > 50 μM (*n* = 1). Each has a SEM ±0.2 log unit.

The optimization of on-target potency, achieved without augmenting the reactivity of the warhead, culminated in a marked enhancement of compound covalent selectivity (Figure 7). The selectivity of **VVD-214** and unoptimized compound **1f** was determined using global proteomics looking at ∼12,800 cysteine sites in OCI-AML2 cells. Both compounds were tested at 20∼30-fold compound concentration over their respective cellular TE_50_’s (**1f** TE_50_ = 2.6 μM; **VVD-214** TE_50_ = 0.065 μM). This comprehensive analysis elucidated a superior selectivity profile for **VVD-214** in comparison to unoptimized compound **1f**.

**Figure 7:**
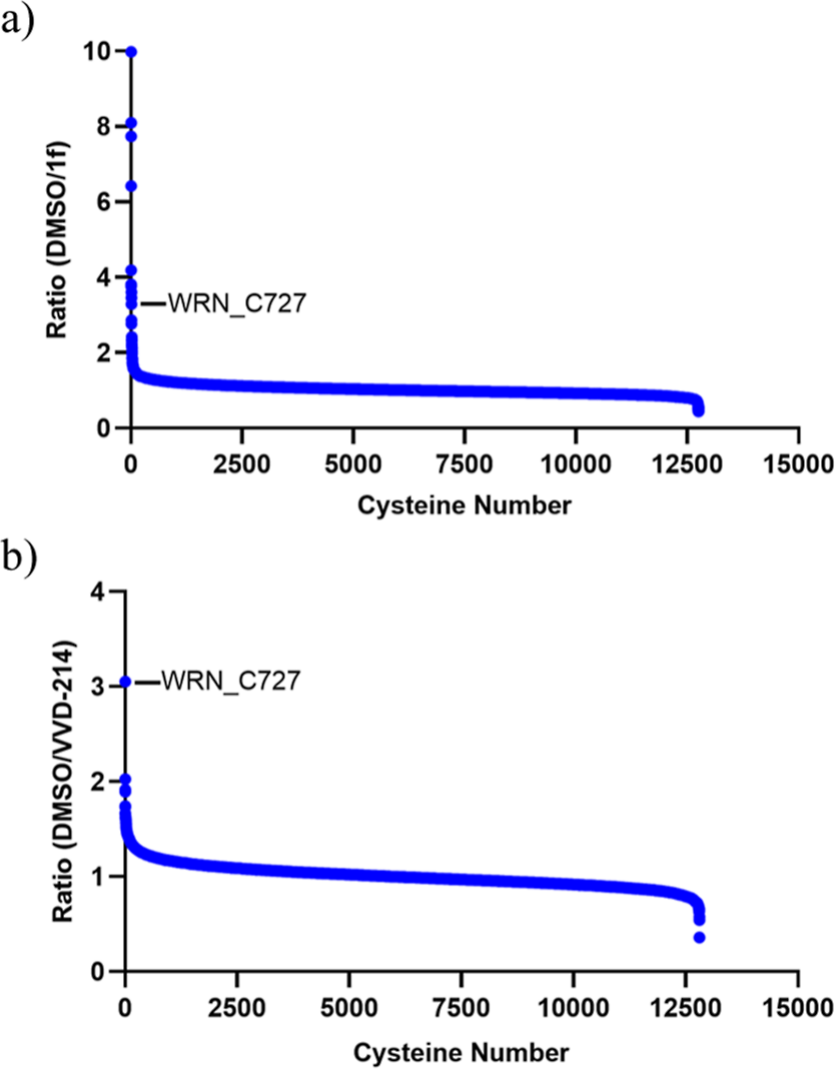
MS-based proteomic cysteine profiling for (a) compound **1f** at 50 μM and (b) **VVD-214** at 2 μM.

Calculated and measured in vitro properties of **VVD-214** are summarized in Table 12. In the helicase assay, **VVD-214** demonstrated a strong nucleotide-cooperative inhibition potency with an IC_50_ of 0.13 μM when pretreated in the presence of 0.2 mM ATP, 18-fold more potent than in the absence of ATP. As observed with other nucleotide-cooperative compounds, **VVD-214** exhibited an enhanced TE_50_ in cells compared to lysate. Also, **VVD-214** potently inhibited the growth of HCT116 cells (MSI-H, GI_50_ = 0.043 μM), while it had no impact on the growth of SW480 cells (MSS) up to 20 μM, the maximum concentration tested. **VVD-214** demonstrated decent kinetic solubility of 169 μM at pH 7.4. An assessment of permeability and efflux by the Caco-2 bilayer system showed good passive permeability of 3.5 × 10^–6^ cm/s (A to B) and a moderate efflux ratio of 12.3.

**Table 12:**
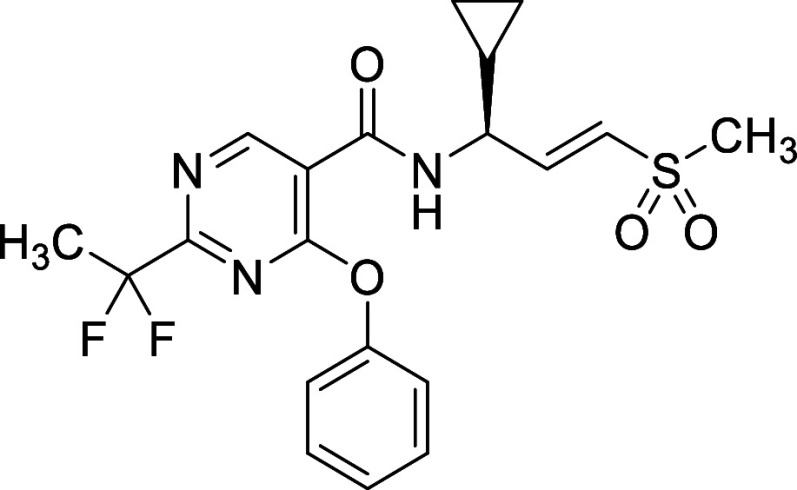
Properties of VVD-214

assay	activity
helicase + 0.2mM ATP IC_50_ [μM]	0.13
helicase without ATP IC_50_ [μM]	2.4
WRN_C727 Cell TE_50_ [μM]	0.065
WRN_C727 Lysate TE_50_ [μM]	0.58
cell growth inhibition, HCT116 GI_50_ [μM]	0.043
cell growth inhibition, SW480 GI_50_ [μM]	>20
MW/LogD_7.4_/TPSA [Å^2^]	437/2.03/98
LipE (pGI_50_–LogD_7.4_)	5.3
kinetic solubility, pH 7.4 [μM]	169
Caco-2 P_app_ A to B [10^–6^ cm/s]/efflux ratio	3.5/12.3
plasma protein binding [%] human	91.9

### Chemistry

The synthesis of **VVD-214** (Scheme 1) involves two main parts: the warhead synthesis and the core synthesis. The vinyl sulfone warhead synthesis commenced with the reduction of methyl ester **9a** to the corresponding alcohol using LAH, followed by Dess–Martin periodinane-mediated oxidation to the corresponding aldehyde **9b**. A Horner–Wadsworth–Emmons olefination of the aldehyde **9b** with diethyl ((methylsulfonyl)­methyl)­phosphonate, followed by Boc group cleavage by TsOH, yielded *trans*-vinyl sulfone **9c** as a tosylate salt. The preparation of the pyrimidine core follows the Pinner pyrimidine synthesis. It started with the formation of the primary amide from carboxylic acid **9d**, followed by conversion to the corresponding imidate through treatment with Meerwein’s salt, and subsequent conversion to the amidine using ammonia in one pot. The resultant amidine **9e** was then condensed with diethyl ethoxymethylenemalonate in the presence of potassium carbonate to form pyrimidine **9f**. The C3 position of the pyrimidine **9f** was chlorinated using phosphoryl chloride, followed by a S_N_Ar reaction with a phenoxide, yielding the corresponding phenyl ether **9g**. Finally, the ethyl ester group was hydrolyzed, and an amide coupling with viny sulfone **9c** was performed using HATU, resulting in the synthesis of **VVD-214**. The synthesis of all other compounds described in the Results and Discussion section was accomplished using similar procedures, detailed in the Experimental Section (see Scheme 2–6).

**Scheme 1:**
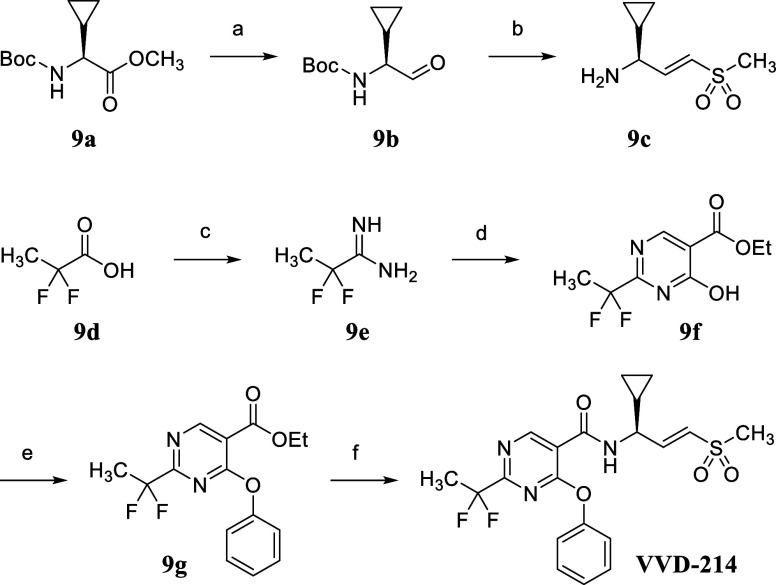
Synthesis of VVD-214^a^

a Reagents and conditions: (a) (i) LAH, THF, 0–25 °C, 8 h; (ii) Dess–Martin periodinane, DCM, 25 °C, 1.5 h; (b) (i) diethyl ((methylsulfonyl)­methyl)­phosphonate, NaH, THF, 25 °C, 1.5 h; (ii) TsOH, MeCN, 60 °C, 4 h; (c) (i) (COCl)_2_/DMF, 2-MeTHF, 0 °C, 1 h; NH_3_, MeOH, 0 °C, 1 h; (ii) Me_3_O­(BF_4_), DCM, 25 °C, 16 h; NH_3_, MeOH, −40–25 °C, 16 h; (d) diethyl ethoxymethylenemalonate, K_2_CO_3_, MeCN, 70 °C, 4 h; (e) (i) POCl_3_, DIPEA, toluene, 90 °C, 5 h; (ii) phenol, K_2_CO_3_, MeCN, 25 °C, 16 h; (e) (i) LiOH, THF/H_2_O, 25 °C, 2 h; (ii) **9c**, HATU, DIPEA, DMF, 25 °C, 2 h.

## Conclusions

The discovery of **VVD-214/RO7589831** represents a significant milestone in the development of WRN inhibitors for the potential treatment of MSI-H cancers. This achievement was facilitated by a chemo-proteomics method, followed by optimization of each component of the hit molecules. Throughout the process, we investigated the SAR of nucleotide cooperativity in helicase assays, the tunable nature of the vinyl sulfone warhead, the impact of warhead orientation, and the SAR of the C2 alkyl group on the pyrimidine core in terms of potency and ADME profile. This report underscores the effective application of chemo-proteomics, covalent small molecules, and medicinal chemistry tailored to covalent chemistry for drug discovery targeting challenging proteins such as helicases.

## Experimental Section

### General Chemistry Methods

Reactions were conducted in standard laboratory glassware or disposable borosilicate vials, with stirring by magnetic stir-bars and stir-plates. For air- and/or moisture-sensitive reactions performed under inert gas, glassware and stir-bars were oven-dried prior to setting-up the reactions. All solvents (technical grade) and chemicals (reagent grade) were procured from commercial vendors and used without further purification. Reaction monitoring and purity assessment were performed using analytical liquid chromatography mass spectrometry (LC–MS) on Shimadzu LC-20AD or Agilent 1200 systems, coupled to ELSD (Agilent G7102A) and MSD (Agilent G6120). LC–MS conditions were as follows: Kinetex EVO C18 30 × 2.1 mm, 5 μm, 5–95% ACN (0.02% TFA) in water (0.04% TFA), with a 1.5 min run at a flow rate of 1.5 mL/min for reaction monitoring, and a 3 min run at a flow rate of 0.8 mL/min for purity assessment, with UV detection at λ = 220 and/or 254 nm. Enantiomeric excess (e.e.) measurement was performed using supercritical fluid chromatography (SFC) on Waters ACQUITY UPC^2^ equipped with PDA (λ = 220 nm), using the following conditions: Chiralpak AD-3, 50 × 4.6 mm, 3 μm, 5–50% IPA (0.1% isopropylamine, v/v) in CO_2_, with a 3 min run at a flow rate of 3.4 mL/min. Flash column chromatography was performed using Biotage Isolera Prime or Teledyne ISCO CombiFlash Rf-150, under the conditions as described. High performance liquid chromatography (HPLC) purification was carried out on a Shimadzu LC-20AD equipped with a liquid handler (Gilson GX-281), under the conditions as described. High-resolution mass measurement was conducted on a Thermo Scientific Orbitrap Exploris 480 Mass Spectrometer (45,000 resolution). Proton (^1^H) and carbon (^13^C­{^1^H}) nuclear magnetic resonance (NMR) spectra were recorded on Bruker Avance 400, Bruker Avance NEO 400, or Bruker Ascend 400 (400 MHz for ^1^H; 101 MHz for ^13^C), and fluorine (^19^F­{^1^H}) NMR spectra were recorded on a 500 MHz Bruker Avance II (470 MHz for ^19^F), using deuterated solvents as described. Chemical shifts (δ) are expressed in parts per million (ppm) and are calibrated to the residual solvent peak. Coupling constants (*J*) are reported in hertz (Hz). Multiplicities are reported using the following abbreviations: s = singlet, d = doublet, dd = doublet of doublets, t = triplet, q = quartet, quint = quintet, m = multiplet, br = broad signal, and dt = doublet of triplets. All final compounds were purified as described to the HPLC purity of >95%.

### Preparation of VVD-214.^38^

#### *tert*-Butyl (*S*)-(1-Cyclopropyl-2-oxoethyl)­carbamate (**9b**)

LAH (6.95 g, 183 mmol, 2.1 equiv) was added to a solution of methyl (*S*)-2-((*tert*-butoxycarbonyl)­amino)-2-cyclopropylacetate (**9a**, 20 g, 87.2 mmol, 1 equiv) in THF (300 mL, 0.29 M) at 0 °C. The mixture was allowed to warm to room temperature and stirred for 8 h. The mixture was quenched by NaHSO_4_·10H_2_O and filtered. The combined organic layers were partitioned between water (600 mL) and EtOAc (100 mL × 3). The combined organic layers were washed with brine (50 mL × 2), dried over anhydrous Na_2_SO_4_, filtered, and concentrated under reduced pressure to give a residue. The residue was purified by flash silica gel chromatography (SiO_2_ 230–400 mesh, eluent of 0–50% EtOAc/petroleum ether gradient) to afford *tert*-butyl (*S*)-(1-cyclopropyl-2-hydroxyethyl)­carbamate (17.6 g, 100% yield) as a white solid. ^1^H NMR (400 MHz, chloroform-*d*): δ 4.83 (br s, 1H), 3.81 (dd, *J* = 11.07, 3.31 Hz, 1H), 3.67 (dd, *J* = 11.07, 6.32 Hz, 1H), 2.95 (br s, 1H), 1.46 (s, 9H), 0.80–0.90 (m, 1H), 0.48–0.60 (m, 2H), 0.28–0.43 (m, 2H). Dess–Martin periodinane (54.8 g, 129 mmol, 2 equiv) was added to a solution of *tert*-butyl (*S*)-(1-cyclopropyl-2-hydroxyethyl)­carbamate (13.0 g, 64.6 mmol, 1 equiv) in DCM (250 mL, 0.26 M) at 0 °C. The mixture was allowed to warm to room temperature and stirred for 1.5 h. The mixture was quenched by saturated aqueous Na_2_S_2_O_3_ (100 mL) and extracted with DCM (50 mL × 3). The combined organic layers were washed with saturated aqueous NaHCO_3_ (50 mL × 3), brine (50 mL), dried over anhydrous Na_2_SO_4_, filtered, and concentrated under reduced pressure to afford the title compound (12.0 g, 93% yield) as a yellow oil. ^1^H NMR (400 MHz, chloroform-*d*): δ 9.65 (br s, 1H), 5.08–5.27 (m, 1H), 3.64–3.79 (m, 1H), 1.45 (s, 9H), 0.86–0.95 (m, 1H), 0.62–0.72 (m, 2H), 0.44–0.51 (m, 2H).

#### (*S*,*E*)-1-Cyclopropyl-3-(methylsulfonyl)­prop-2-en-1-amine (**9c**)

Sodium hydride (2.4 g, 60.7 mmol, 60 wt %, 1.1 equiv) was added to a solution of diethyl ((methylsulfonyl)­methyl)­phosphonate (15.3 g, 66.2 mmol, 1.2 equiv) in THF (150 mL, 0.37 M) at 0 °C. After 1 h, the mixture was added *tert*-butyl (*S*)-(1-cyclopropyl-2-oxoethyl)­carbamate (**9b**, 11.0 g, 55.2 mmol, 1 equiv) and allowed to warm to room temperature. After 1.5 h, the mixture was added to saturated aqueous NH_4_Cl (400 mL) and extracted with EtOAc (200 mL × 3). The combined organic layers were washed with brine (50 mL × 2), dried over anhydrous Na_2_SO_4_, filtered, and concentrated under reduced pressure to give a residue. The residue was purified by flash silica gel chromatography (SiO_2_ 230–400 mesh, eluent of 0–50% EtOAc/petroleum ether gradient) to afford *tert*-*butyl* (*S,E*)-(1-cyclopropyl-3-(methylsulfonyl)­allyl)­carbamate (7.3 g, 48% yield) as a white solid. E.e. = 100%. ^1^H NMR (400 MHz, chloroform-*d*): δ 6.93 (dd, *J* = 15.13, 4.63 Hz, 1H), 6.53 (dd, *J* = 15.13, 1.50 Hz, 1H), 4.60–4.88 (m, 1H), 3.68 (br s, 1H), 2.96 (s, 3H), 1.46 (s, 10H), 0.81–0.96 (m, 1H), 0.58–0.74 (m, 2H), 0.33–0.50 (m, 2H). A mixture of *p*-toluenesulfonic acid monohydrate (4.14 g, 21.8 mmol, 1.2 equiv) and *tert*-butyl (*S,E*)-(1-cyclopropyl-3-(methylsulfonyl)­allyl)­carbamate (5 g, 18.2 mmol, 1 equiv) in MeCN (5 mL, 3.6 M) was heated at 60 °C for 4 h. After cooling to room temperature, the precipitate was collected by filtration to afford the title compound as a tosylate salt (5.7 g, 90% yield) as a white solid. ^1^H NMR (400 MHz, DMSO-*d*_6_): δ 8.28 (br s, 2H), 7.48 (d, *J* = 8.0 Hz, 2H), 7.12 (d, *J* = 8.0 Hz, 2H), 7.03 (d, *J* = 15.3 Hz, 1H), 6.75 (dd, *J* = 6.2, 15.5 Hz, 1H), 3.45 (s, 1H), 3.34 (br s, 1H), 3.07 (s, 3H), 2.29 (s, 3H), 1.12–0.99 (m, 1H), 0.63 (br d, *J* = 8.0 Hz, 2H), 0.56–0.49 (m, 1H), 0.46–0.38 (m, 1H).

#### 2,2-Difluoropropanimidamide (**9e**)

Oxalyl chloride (63.4 g, 500 mmol, 1.1 equiv) was added to a solution of 2,2-difluoropropanoic acid (**9d**, 50.0 g, 454 mmol, 1.0 equiv) and DMF (100 mg, catalytic amount) in 2-MeTHF (200 mL, 2.5 M) at 0 °C under an atmosphere of dry N_2_. After 2 h, ammonia in methanol (99.3 mL, 1136 mmol, 2.5 equiv) was added to the reaction mixture at 0 °C. After stirring for 1 h, the mixture was allowed to warm to room temperature and filtered. The filtrate was washed with brine (50 mL), dried over anhydrous Na_2_SO_4_, filtered, and concentrated under reduced pressure to afford 2,2-difluoropropanamide (35.0 g, 71% yield) as an off-white solid. ^1^H NMR (400 MHz, chloroform-*d*): δ 6.20 (br s, 2H), 1.75 (t, *J*_HF_ = 19.3 Hz, 3H). Trimethyloxonium tetrafluoroborate (Meerwein’s salt, 13.6 g, 91.7 mmol, 1 equiv) was added to a solution of 2,2-difluoropropanamide (10.0 g, 91.7 mmol, 1 equiv) in DCM (100 mL, 0.92 M) at 0 °C. The mixture was allowed to warm to room temperature and stirred for 16 h under an atmosphere of dry N_2_. The mixture was cooled to −40 °C, and ammonia in methanol (30.6 mL, 1400 mmol, 15 equiv) was added to the mixture. The mixture was allowed to warm to room temperature and stirred for 16 h under an atmosphere of dry N_2_. The mixture was filtered, and the filtrate was concentrated under reduced pressure to afford the title compound (11.5 g, ∼70% purity) as a yellow oil, which was used in the subsequent reaction without further purification. MS (ESI^+^): *m*/*z* 109.1 [M + H]^+^.

#### Ethyl 2-(1,1-difluoroethyl)-4-hydroxypyrimidine-5-carboxylate (**9f**)

A suspension of 2,2-difluoropropanimidamide (**9e**, 11.5 g, ∼70% purity, 74.5 mmol, 1 equiv), diethyl ethoxymethylenemalonate (20.7 g, 95.8 mmol, 1.3 equiv), and potassium carbonate (29.4 g, 212.8 mmol, 2.9 equiv) in MeCN (100 mL, 0.75 M) was heated at 70 °C for 4 h. After cooling to room temperature, the mixture was concentrated under reduced pressure to give a residue. The residue was taken up in MTBE (30 mL) and EtOAc (100 mL), and the mixture was stirred for 16 h. The formed precipitate was collected and added to EtOAc (200 mL). The mixture was added HCl (6 M in water) to a pH of 3 and extracted with EtOAc (50 mL × 2). The combined organic layers were washed with brine (30 mL), dried over anhydrous Na_2_SO_4_, filtered, and concentrated under reduced pressure to give a residue. The residue was added to MTBE (30 mL) and stirred for 2 h. The formed precipitate was collected, rinsed with MTBE (5 mL), and dried to afford the title compound (11.3 g, 53% yield over 2 steps) as an off-white solid. MS (ESI^+^): *m*/*z* 233.2 [M + H]^+^. ^1^H NMR (400 MHz, chloroform-*d*): δ 11.63 (br s, 1H), 9.02 (s, 1H), 4.50 (q, *J* = 7.2 Hz, 2H), 2.04 (t, *J*_HF_ = 18.7 Hz, 3H), 1.45 (t, *J* = 7.2 Hz, 3H).

#### Ethyl 2-(1,1-difluoroethyl)-4-phenoxypyrimidine-5-carboxylate (**9g**)

To a solution of ethyl 2-(1,1-difluoroethyl)-4-hydroxypyrimidine-5-carboxylate (**9f**, 10.8 g, 46.5 mmol, 1 equiv) and DIPEA (24.3 mL, 140 mmol, 3 equiv) in toluene (100 mL, 0.47 M) was added phosphorus oxychloride (14.3 g, 93.0 mmol, 2 equiv) at room temperature. The mixture was heated at 90 °C for 5 h under an atmosphere of dry N_2_. After cooling to room temperature, the mixture was quenched by ice–water (50 mL) and extracted with EtOAc (25 mL × 3). The combined organic layers were washed with saturated aqueous NaHCO_3_ (30 mL), brine (30 mL), dried over anhydrous Na_2_SO_4_, filtered, and concentrated under reduced pressure to afford crude ethyl 4-chloro-2-(1,1-difluoroethyl)­pyrimidine-5-carboxylate (11.1 g, 95% yield) as a brown oil, which was used in the subsequent reaction without further purification. MS (ESI^+^): *m*/*z* 251.2 [M + H]^+^. ^1^H NMR (400 MHz, chloroform-*d*): δ 9.20 (s, 1H), 4.49 (q, *J* = 7.1 Hz, 2H), 2.08 (t, *J*_HF_ = 18.5 Hz, 3H), 1.45 (t, *J* = 7.2 Hz, 3H). A suspension of the crude ethyl 4-chloro-2-(1,1-difluoroethyl)­pyrimidine-5-carboxylate (11.1 g, 44.3 mmol, 1 equiv), phenol (4.38 g, 46.5 mmol, 1.05 equiv), and potassium carbonate (9.18 g, 66.4 mmol, 1.5 equiv) in MeCN (100 mL, 0.44 mmol) was stirred at room temperature for 16 h. The mixture was filtered and concentrated under reduced pressure to give a residue. The residue was taken up in EtOAc (100 mL), washed with water (30 mL), brine (30 mL), dried over anhydrous Na_2_SO_4_, filtered, and concentrated under reduced pressure to afford ethyl 2-(1,1-difluoroethyl)-4-phenoxypyrimidine-5-carboxylate (14.0 g, 100% yield) as a brown solid. MS (ESI^+^): *m*/*z* 309.2 [M + H]^+^. ^1^H NMR (400 MHz, chloroform-*d*): δ 9.19 (s, 1H), 7.48–7.39 (m, 2H), 7.33–7.27 (m, 1H), 7.22–7.16 (m, 2H), 4.47 (q, *J* = 7.1 Hz, 2H), 1.84 (t, *J*_HF_ = 18.5 Hz, 3H), 1.44 (t, *J* = 7.2 Hz, 3H).

#### (*S*,*E*)-*N*-(1-cyclopropyl-3-(methylsulfonyl)­allyl)-2-(1,1-difluoroethyl)-4-phenoxypyrimidine-5-carboxamide (VVD-214)

Lithium hydroxide monohydrate (3.81 g, 90.8 mmol, 2 equiv) was added to a solution of ethyl 2-(1,1-difluoroethyl)-4-phenoxypyrimidine-5-carboxylate (**9g**, 14.0 g, 45.4 mmol, 1 equiv) in THF and water (1:1, 150 mL, 0.32 M) at room temperature. After stirring for 2 h, the mixture was concentrated to half the volume to remove organic solvents. 4 M HCl was added to the mixture to adjust the pH to 2. The formed precipitate was collected and dried to afford 2-(1,1-difluoroethyl)-4-phenoxypyrimidine-5-carboxylic acid (9.20 g, 71% yield over 3 steps) as an off-white solid. MS (ESI^+^): *m*/*z* 281.2 [M + H]^+^. ^1^H NMR (400 MHz, DMSO-*d*_6_): δ 13.83 (br s, 1H), 9.18 (s, 1H), 7.54–7.39 (m, 2H), 7.35–7.29 (m, 1H), 7.29–7.22 (m, 2H), 1.83 (t, *J*_HF_ = 19.0 Hz, 3H). HATU (3.05 g, 8.03 mmol, 1.5 equiv) was added to a solution of 2-(1,1-difluoroethyl)-4-phenoxypyrimidine-5-carboxylic acid (1.5 g, 5.35 mmol, 1 equiv), (*S*,*E*)-1-cyclopropyl-3-(methylsulfonyl)­prop-2-en-1-amine tosylate salt (**9c**, 1.86 g, 5.35 mmol, 1 equiv), and DIPEA (2.80 mL, 16.1 mmol, 3 equiv) in DMF (20 mL, 0.27 M) at room temperature. After stirring for 2 h under an atmosphere of dry N_2_, the mixture was concentrated under reduced pressure to give a residue. The residue was diluted with water (50 mL) and extracted with EtOAc (15 mL × 3). The combined organic layers were washed with brine, dried over anhydrous Na_2_SO_4_, filtered, and concentrated under reduced pressure to give a residue. The residue was purified by prep-HPLC (Welch Xtimate C18, 250 × 70 mm, 10 μm, 30–60% ACN in water (10 mM NH_4_HCO_3_)) to afford the title compound (2.28 g, 98% yield) as a white solid. Purity = 100%, e.e. = 100%. MS (ESI^+^): *m*/*z* 438.1 [M + H]^+^. HR-MS (ESI+): *m*/*z* calcd for [C_20_H_21_F_2_N_3_O_4_S + H]^+^: 438.1294; found, 438.1297. ^1^H NMR (400 MHz, chloroform-*d*): δ 9.42–9.55 (m, 1H), 7.75–7.81 (m, 1H), 7.49–7.54 (m, 2H), 7.37–7.42 (m, 1H), 7.23–7.26 (m, 2H), 7.03 (dd, *J* = 15.20, 4.94 Hz, 1H), 6.59–6.64 (m, 1H), 4.22–4.30 (m, 1H), 2.97 (s, 3H), 1.86 (t, *J*_HF_ = 18.51 Hz, 3H), 1.03–1.14 (m, 1H), 0.66–0.79 (m, 2H), 0.47–0.54 (m, 2H). ^1^H NMR (400 MHz, DMSO-*d*_6_): δ 9.03 (s, 1H), 8.94 (d, *J* = 8.3 Hz, 1H), 7.54–7.47 (m, 2H), 7.37–7.29 (m, 3H), 6.95–6.82 (m, 2H), 4.30 (m, 1H), 2.98 (s, 3H), 1.87 (t, *J*_HF_ = 19.0 Hz, 3H), 1.16–1.07 (m, 1H), 0.61–0.53 (m, 1H), 0.53–0.45 (m, 2H), 0.44–0.36 (m, 1H). ^13^C NMR (101 MHz, DMSO-*d*_6_): δ 165.47, 161.87 (t, *J*_CF_ = 29.1 Hz), 161.65, 158.73, 151.51, 144.93, 130.11, 129.67, 126.00, 121.55, 118.90 (t, *J*_CF_ = 241.0 Hz), 118.63, 53.00, 42.21, 22.48 (t, *J*_CF_ = 26.0 Hz), 14.46, 3.06, 2.86. ^19^F NMR (470 MHz, DMSO-*d*_6_): δ −92.19. LC–MS report, SFC report, and NMR spectra for **VVD-214** are included in Figure S6.

### General Procedure I: Oxidation of Primary Alcohol to Aldehyde

To a solution of the appropriate primary alcohol (1 equiv) in DCM (0.2 M) was added Dess–Martin periodinane (2 equiv) at room temperature. After stirring at room temperature under an atmosphere of dry N_2_ for 3 h, the mixture was quenched by saturated aqueous Na_2_S_2_O_3_ and extracted with DCM (three times). The combined organic layers were washed with saturated aqueous NaHCO_3_ (twice), washed with brine, dried over anhydrous Na_2_SO_4_, filtered, and concentrated under reduced pressure to afford the corresponding crude aldehyde, which was used in subsequent reactions without further purification.

**Scheme 2:**
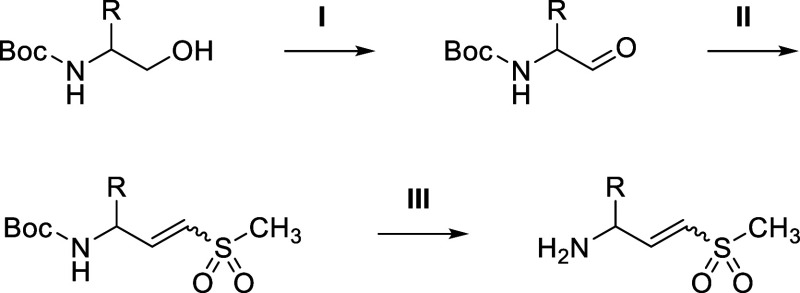
Synthesis of the Vinyl Sulfone Warhead

### General Procedure II: Horner–Wadsworth–Emmons Reaction for E and Z-Olefin Isomers Synthesis

A mixture of diethyl ((methylsulfonyl)­methyl)­phosphonate (1.2 equiv) and sodium hydride (1.1 equiv) in THF (0.5 M) was stirred at 0 °C for 1 h. After adding the appropriate aldehyde (1 equiv) in THF (1.5 M), the mixture was allowed to warm to room temperature and stirred for 1 h. The reaction mixture was poured into ice water and extracted with EtOAc (three times). The combined organic layers were washed with brine (twice), dried over anhydrous Na_2_SO_4_, filtered, and concentrated under reduced pressure to give a residue. The residue was purified as described to afford the corresponding E-olefin as the major product and Z-olefin as the minor product.

### General Procedure III: Boc Deprotection Using TsOH

To a solution of the appropriate *N*-Boc-protected intermediate (1 equiv) in MeCN (0.32 M) was added *p*-toluenesulfonic acid monohydrate (1.2 equiv). The mixture was stirred at 50 °C for 12 h. The mixture was cooled to room temperature, and the formed precipitate was collected by filtration to afford the crude amine as the tosylate salt, which was used in subsequent reactions without further purification.

#### (*E*)-3-(methylsulfonyl)­prop-2-en-1-amine Tosylate Salt

Commercially available *tert*-butyl (2-oxoethyl)­carbamate (6 g, 26.06 mmol) was used for General Procedure II and purified by flash silica gel chromatography (ISCO; 120g SepaFlash Silica Flash Column, eluent of 20–25% EtOAc/petroleum ether gradient at 100 mL/min) to afford *tert*-butyl (*E*)-(3-(methylsulfonyl)­allyl)­carbamate (5 g, 82% yield) as a white solid. ^1^H NMR (400 MHz, chloroform-*d*): δ 6.86–6.97 (m, 1H), 6.44–6.58 (m, 1H), 4.84 (br s, 1H), 4.00 (br s, 2H), 2.95 (s, 3H), 1.46 (s, 9H). *tert*-Butyl (*E*)-(3-(methylsulfonyl)­allyl)­carbamate (3 g, 12.75 mmol) was used for General Procedure III to afford the title compound as a white solid. ^1^H NMR (400 MHz, DMSO-*d*_6_): δ 8.10 (br s, 3H), 7.50 (d, *J* = 8.0 Hz, 2H), 7.13 (d, *J* = 7.9 Hz, 2H), 6.99 (d, *J* = 15.4 Hz, 1H), 6.75 (td, *J* = 5.5, 15.4 Hz, 1H), 3.76 (dd, *J* = 1.4, 5.5 Hz, 2H), 3.05 (s, 3H), 2.36–2.23 (m, 3H).

#### (*S*,*E*)-4-(Methylsulfonyl)­but-3-en-2-amine Tosylate Salt

*tert*-Butyl (*S*)-(1-hydroxypropan-2-yl)­carbamate (5 g, 57.07 mmol) was used for General Procedure I to afford *tert*-butyl (*S*)-(1-oxopropan-2-yl)­carbamate (4.95 g, 100%) as a yellow solid. ^1^H NMR (400 MHz, chloroform-*d*): δ 9.52 (s, 1H), 5.23 (br s, 1H), 4.25–4.11 (m, 1H), 1.41 (s, 9H), 1.29 (d, *J* = 7.2 Hz, 3H). *tert*-Butyl (*S*)-(1-oxopropan-2-yl)­carbamate (10.6 g, 46.19 mmol) was used for General Procedure II and purified by flash silica gel chromatography (ISCO; 80g SepaFlash Silica Flash Column, eluent of 20–50% EtOAc/petroleum ether gradient at 75 mL/min) to afford *tert*-butyl (*S,Z*)-(4-(methylsulfonyl)­but-3-en-2-yl)­carbamate (0.43 g, 3.7% yield) as the first peak and *tert*-butyl (*S,E*)-(4-(methylsulfonyl)­but-3-en-2-yl)­carbamate (5.0 g, 43% yield) as the second peak. *Z*-isomer: ^1^H NMR (400 MHz, chloroform-*d*): δ 6.28–6.24 (m, 1H), 6.20–6.13 (m, 1H), 5.21–5.10 (m, 1H), 4.68–4.57 (m, 1H), 3.20 (br s, 3H), 1.47–1.41 (m, 9H), 1.32 (d, *J* = 6.9 Hz, 3H). *E*-isomer: ^1^H NMR (400 MHz, chloroform-*d*): δ 6.86 (dd, *J* = 4.6, 15.1 Hz, 1H), 6.47 (dd, *J* = 1.4, 15.1 Hz, 1H), 4.60–4.41 (m, 2H), 2.95 (s, 3H), 1.46 (s, 9H), 1.36–1.29 (m, 3H). *tert*-Butyl (*S,E*)-(4-(methylsulfonyl)­but-3-en-2-yl)­carbamate (6.9 g, 27.7 mmol) was used for General Procedure III to afford the title compound (7 g, 79% yield) as a yellow solid. ^1^H NMR (400 MHz, DMSO-*d*_6_): δ 8.15 (br s, 3H), 7.49 (d, *J* = 8.0 Hz, 2H), 7.12 (d, *J* = 7.9 Hz, 2H), 6.99 (dd, *J* = 15.4, 1.3 Hz, 1H), 6.75 (dd, *J* = 15.4, 5.8 Hz, 1H), 4.11 (br s, 1H), 3.05 (s, 3H), 2.29 (s, 3H), 1.33 (d, *J* = 6.8 Hz, 3H).

#### (*S*,*Z*)-4-(Methylsulfonyl)­but-3-en-2-amine Tosylate Salt

*tert*-Butyl (*S,Z*)-(4-(methylsulfonyl)­but-3-en-2-yl)­carbamate (2.5 g, 10.0 mmol) was used for General Procedure III to afford the title compound (2.4, 74% yield) as a white solid. ^1^H NMR (400 MHz, DMSO-*d*_6_): δ 8.06 (br s, 3H), 7.54–7.45 (m, 2H), 7.13 (d, *J* = 7.9 Hz, 2H), 6.80–6.70 (m, 1H), 6.35 (dd, *J* = 9.4, 11.2 Hz, 1H), 4.84 (br d, *J* = 3.1 Hz, 1H), 3.14 (s, 3H), 2.29 (s, 3H), 1.31 (d, *J* = 6.6 Hz, 3H).

#### (*R*,*E*)-4-(Methylsulfonyl)­but-3-en-2-amine Tosylate Salt

*tert*-Butyl (*R*)-(1-hydroxypropan-2-yl)­carbamate was used instead of *tert*-butyl (*S*)-(1-hydroxypropan-2-yl)­carbamate at the first step of the synthesis route for (*S,E*)-4-(methylsulfonyl)­but-3-en-2-amine tosylate salt to afford the title compound as a white solid. ^1^H NMR (400 MHz, DMSO-*d*_6_): δ 8.13 (br s, 3H), 7.48 (d, *J* = 8.0 Hz, 2H), 7.12 (d, *J* = 7.9 Hz, 2H), 6.99 (dd, *J* = 1.3, 15.5 Hz, 1H), 6.75 (dd, *J* = 5.8, 15.4 Hz, 1H), 4.11 (br s, 1H), 3.05 (s, 3H), 2.29 (s, 3H), 1.33 (d, *J* = 6.8 Hz, 3H).

#### (*R*,*Z*)-4-(Methylsulfonyl)­but-3-en-2-amine Tosylate Salt

*tert*-Butyl (*R,Z*)-(4-(methylsulfonyl)­but-3-en-2-yl)­carbamate was used instead of *tert*-butyl (*S,Z*)-(4-(methylsulfonyl)­but-3-en-2-yl)­carbamate for the synthesis route of (*S,Z*)-4-(methylsulfonyl)­but-3-en-2-amine tosylate salt to afford the title compound as a white solid. ^1^H NMR (400 MHz, DMSO-*d*_6_): δ 8.07 (br s, 3H), 7.49 (d, *J* = 8.0 Hz, 2H), 7.13 (d, *J* = 7.9 Hz, 2H), 6.76 (d, *J* = 11.3 Hz, 1H), 6.35 (dd, *J* = 9.5, 11.2 Hz, 1H), 4.85 (br d, *J* = 4.5 Hz, 1H), 3.13 (s, 3H), 2.29 (s, 3H), 1.31 (d, *J* = 6.7 Hz, 3H).

#### 3-(Methylsulfonyl)­propan-1-amine Tosylate Salt

A mixture of *tert*-butyl (*E*)-(3-(methylsulfonyl)­allyl)­carbamate (500 mg, 2.12 mmol) and Pd/C (50 mg, wet) in MeOH (5 mL, 0.42 M) was stirred under an atmosphere of hydrogen gas (balloon, 15 psi) at room temperature for 2 h. The mixture was filtered and rinsed with MeOH (5 mL × 3). The combined filtrates were concentrated under reduced pressure to afford *tert*-butyl (3-(methylsulfonyl)­propyl)­carbamate (500 mg, 99% yield) as a white solid, which was used in the subsequent reaction without further purification. ^1^H NMR (400 MHz, chloroform-*d*): δ 4.74 (br s, 1H), 3.36–3.26 (m, 2H), 3.12–3.05 (m, 2H), 2.93 (s, 3H), 2.12–2.00 (m, 2H), 1.45 (s, 9H). *tert*-Butyl (3-(methylsulfonyl)­propyl)­carbamate (300 mg, 1.26 mmol) was used for General Procedure III to afford the title compound (330 mg, 84% yield) as a white solid. ^1^H NMR (400 MHz, DMSO-*d*_6_): δ 7.48 (d, *J* = 8.0 Hz, 2H), 7.11 (d, *J* = 7.9 Hz, 2H), 3.26–3.20 (m, 2H), 2.99 (s, 3H), 2.93 (br s, 2H), 2.29 (s, 3H), 2.02–1.91 (m, 2H).

#### (*S*,*E*)-1-Cyclobutyl-3-(methylsulfonyl)­prop-2-en-1-amine Tosylate Salt

LAH (636 mg, 16.77 mmol, 2.4 equiv) was added to a solution of methyl (*S*)-2-((*tert*-butoxycarbonyl)­amino)-2-cyclobutylacetate in THF (50 mL, 0.14 M) at 0 °C, and the mixture was allowed to warm to room temperature. After stirring for 8 h, the mixture was quenched by NaHSO_4_·10H_2_O and filtered. The filter cake was washed with EtOAc (8 mL × 3), and the combined filtrates were concentrated under reduced pressure to give a residue. The residue was purified by flash silica gel chromatography (Biotage; 20g Agela Flash Silica Flash Column, eluent of 0–60% EtOAc/petroleum ether) to afford *tert*-butyl (*S*)-(1-cyclobutyl-2-hydroxyethyl)­carbamate (1.5 g, 100% yield) as a yellow oil. ^1^H NMR (400 MHz, chloroform-*d*): δ 4.58 (br s, 1H), 3.65–3.61 (m, 1H), 3.47–3.40 (m, 1H), 2.43–2.34 (m, 1H), 2.27 (br s, 1H), 2.04–1.97 (m, 2H), 1.74 (br s, 4H), 1.45 (s, 9H). *tert*-Butyl (*S*)-(1-cyclobutyl-2-hydroxyethyl)­carbamate (1.5 g, 6.97 mmol) was used for General Procedure I to afford *tert*-butyl (*S*)-(1-cyclobutyl-2-oxoethyl)­carbamate (1.3 g, 87% yield) as a yellow oil. ^1^H NMR (400 MHz, DMSO-*d*_6_): δ 9.38 (d, *J* = 1.4 Hz, 1H), 7.30–7.24 (m, 1H), 3.90–3.80 (m, 1H), 2.63–2.56 (m, 1H), 1.88–1.77 (m, 4H), 1.82–1.76 (m, 3H), 1.40 (s, 9H). *tert*-Butyl (*S*)-(1-cyclobutyl-2-oxoethyl)­carbamate (1.0 g, 4.69 mmol) was used for General Procedure II and purified by flash silica gel chromatography (ISCO; 20g SepaFlash Silica Flash Column, eluent of 0–50% EtOAc/petroleum ether gradient) to afford *tert*-butyl (*S,E*)-(1-cyclobutyl-3-(methylsulfonyl)­allyl)­carbamate (450 mg, 33% yield) as a white solid. ^1^H NMR (400 MHz, chloroform-*d*): δ 6.79 (dd, *J* = 4.9, 15.1 Hz, 1H), 6.47 (dd, *J* = 1.6, 15.1 Hz, 1H), 4.49 (br d, *J* = 8.3 Hz, 1H), 4.31 (br s, 1H), 2.94 (s, 3H), 2.43–2.34 (m, 1H), 2.11–2.04 (m, 2H), 1.95–1.82 (m, 4H), 1.46 (s, 9H). *tert*-Butyl (*S,E*)-(1-cyclobutyl-3-(methylsulfonyl)­allyl)­carbamate (450 mg, 1.56 mmol) was used for General Procedure III to afford the title compound (400 mg, 71% yield) as a white solid. ^1^H NMR (400 MHz, DMSO-*d*_6_): δ 7.50–7.46 (m, 2H), 7.12 (d, *J* = 7.8 Hz, 2H), 7.01 (dd, *J* = 0.9, 15.3 Hz, 1H), 6.57 (dd, *J* = 7.4, 15.4 Hz, 1H), 3.96 (t, *J* = 8.3 Hz, 1H), 3.05 (s, 3H), 2.58–2.52 (m, 1H), 2.29 (s, 3H), 2.08–1.98 (m, 1H), 1.73 (br s, 5H).

#### (*R*,*E*)-1-Methoxy-4-(methylsulfonyl)­but-3-en-2-amine Tosylate Salt

(Trimethylsilyl)­diazomethane (2 M in hexane, 4.56 mL, 9.12 mmol, 2 equiv) was added to a solution of *N*-(*tert*-butoxycarbonyl)-*O*-methyl- *l* -serine (1 g, 4.56 mmol, 1 equiv) in DCM (10 mL) and MeOH (1 mL) at 0 °C. After 2 h, the reaction mixture was quenched by dropwise addition of AcOH at 0 °C until gas generation ceased. The mixture was concentrated under reduced pressure to afford crude methyl *N*-(*tert*-butoxycarbonyl)-*O*-methyl- *l* -serinate (1.1 g) as a yellow oil. ^1^H NMR (400 MHz, chloroform-*d*): δ 5.39–5.37 (m, 1H), 4.43–4.41 (m, 1H), 3.82–3.79 (m, 1H), 3.77 (s, 3H), 3.61–3.58 (m, 1H), 3.35 (s, 3H), 1.46 (s, 9H). LAH (469 mg, 12.35 mmol, 2.4 equiv) was added to a solution of methyl *N*-(*tert*-butoxycarbonyl)-*O*-methyl- *l* -serinate (1.2 g, 5.14 mmol, 1 equiv) in THF (20 mL, 0.25 M) at 0 °C, and the mixture was allowed to warm to room temperature. After stirring for 8 h, the mixture was quenched by NaHSO_4_·10H_2_O and filtered. The filter cake was washed with EtOAc (10 mL × 3), and the combined filtrates were concentrated under reduced pressure to give a residue. The residue was purified by flash silica gel chromatography (ISCO; 20g SepaFlash Silica Flash Column, eluent of 0–50% EtOAc/petroleum ether gradient) to afford *tert*-butyl (*R*)-(1-hydroxy-3-methoxypropan-2-yl)­carbamate (1.0 g, 95% yield) as a yellow oil. ^1^H NMR (400 MHz, chloroform-*d*): δ 5.17 (br s, 1H), 3.81–3.79 (m, 2H), 3.71–3.68 (m, 1H), 3.57–3.53 (m, 2H), 3.37 (s, 3H), 2.35 (br s, 1H), 1.46 (s, 9H). *tert*-Butyl (*R*)-(1-hydroxy-3-methoxypropan-2-yl)­carbamate (4.87 g, 23.7 mmol) was used for General Procedure I to afford *tert*-butyl (*S*)-(1-methoxy-3-oxopropan-2-yl)­carbamate (4.30 g, 89% yield) as a yellow oil, which was then used for General Procedure II and purified by flash silica gel chromatography (ISCO; 80g SepaFlash Silica Flash Column, eluent of 50% EtOAc/petroleum ether) to afford *tert*-butyl (*R,E*)-(1-methoxy-4-(methylsulfonyl)­but-3-en-2-yl)­carbamate (1.5 g, 25% yield) as a yellow oil. ^1^H NMR (400 MHz, chloroform-*d*): δ 6.92 (dd, *J* = 4.7, 15.1 Hz, 1H), 6.55 (dd, *J* = 1.7, 15.1 Hz, 1H), 4.53 (br s, 1H), 3.52 (d, *J* = 4.0 Hz, 2H), 3.50–3.49 (m, 1H), 3.36 (s, 3H), 2.96 (s, 3H), 1.46 (s, 9H). *tert*-Butyl (*R,E*)-(1-methoxy-4-(methylsulfonyl)­but-3-en-2-yl)­carbamate (1.5 g, 5.37 mmol) was used for General Procedure III to afford the title compound (1.2 g, 64% yield) as a white solid. ^1^H NMR (400 MHz, DMSO-*d*_6_): δ 8.30 (br s, 3H), 7.48 (d, *J* = 7.95 Hz, 2H), 7.11 (d, *J* = 7.82 Hz, 2H), 7.02 (dd, *J* = 15.53, 1.34 Hz, 1H), 6.70 (dd, *J* = 15.53, 6.11 Hz, 1H), 4.20–4.28 (m, 1H), 3.53–3.65 (m, 2H), 3.33 (s, 3H), 3.05 (s, 3H), 2.29 (s, 3H).

#### (*R*,*E*)-1-(Difluoromethoxy)-4-(methylsulfonyl)­but-3-en-2-amine Tosylate Salt

Potassium hydrogenfluoride (3.51 g, 44.97 mmol, 5.2 equiv) was added to a mixture of *tert*-butyl (*S*)-4-(hydroxymethyl)-2,2-dimethyloxazolidine-3-carboxylate (2.0 g, 8.65 mmol, 1 equiv) and (bromodifluoromethyl)­trimethylsilane (4.47 g, 22.0 mmol, 2.5 equiv) in DCM (10 mL) and water (10 mL) at room temperature. The mixture was heated at 50 °C for 16 h. After cooling to room temperature, the mixture was poured into water (30 mL) and extracted with DCM (30 mL × 3). The combined organic layers were washed with brine (20 mL × 2), dried over anhydrous Na_2_SO_4_, filtered, and concentrated under reduced pressure to give a residue. The residue was purified by flash silica gel chromatography (ISCO; 20g SepaFlash Silica Flash Column, eluent of 0–5% EtOAc/petroleum ether gradient at 100 mL/min) to afford *tert*-butyl (*R*)-4-((difluoromethoxy)­methyl)-2,2-dimethyloxazolidine-3-carboxylate (1.2 g, 49% yield) as a yellow oil. ^1^H NMR (400 MHz, chloroform-*d*): δ 6.22 (t, *J*_HF_ = 74.4 Hz, 1H), 4.18–4.00 (m, 2H), 3.99 (s, 2H), 3.84–3.64 (m, 1H), 1.57–1.45 (m, 15H). *p*-Toluenesulfonic acid monohydrate (162 mg, 0.85 mmol, 0.2 equiv) was added to *tert*-butyl (*R*)-4-((difluoromethoxy)­methyl)-2,2-dimethyloxazolidine-3-carboxylate (1.2 g, 4.27 mmol, 1 equiv) in MeOH (20 mL, 0.21 M), and the resultant mixture was stirred at room temperature for 16 h. The mixture was poured into water (30 mL) and extracted with DCM (30 mL × 3). The combined organic layers were washed with brine (10 mL × 2), dried over anhydrous Na_2_SO_4_, filtered, and concentrated under reduced pressure to give a residue. The residue was purified by flash silica gel chromatography (ISCO; 12g SepaFlash Silica Flash Column, eluent of 0–20% EtOAc/petroleum ether gradient at 100 mL/min) to afford *tert*-butyl (*R*)-(1-(difluoromethoxy)-3-hydroxypropan-2-yl)­carbamate (0.80 g, 78% yield) as a colorless oil. ^1^H NMR (400 MHz, chloroform-*d*): δ 6.24 (t, *J*_HF_ = 74.3 Hz, 1H), 4.96 (br d, *J* = 1.6 Hz, 1H), 4.05–3.93 (m, 2H), 3.87 (br s, 1H), 3.84–3.76 (m, 1H), 3.76–3.68 (m, 1H), 1.46 (s, 9H). *tert*-Butyl (*R*)-(1-(difluoromethoxy)-3-hydroxypropan-2-yl)­carbamate (400 mg, 1.66 mmol) was used for General Procedure I to afford *tert*-butyl (*S*)-(1-(difluoromethoxy)-3-oxopropan-2-yl)­carbamate (400 mg, 100% yield) as a yellow solid. ^1^H NMR (400 MHz, chloroform-*d*): δ 9.65 (s, 1H), 6.21 (t, *J*_HF_ = 73.5 Hz, 1H), 5.36 (br d, *J* = 2.6 Hz, 1H), 4.41 (br s, 1H), 4.39 (br s, 1H), 4.25–4.14 (m, 1H), 1.48 (s, 9H). *tert*-Butyl (*S*)-(1-(difluoromethoxy)-3-oxopropan-2-yl)­carbamate (400 mg, 1.66 mmol) was used for General Procedure II and purified by flash silica gel chromatography (ISCO; 12g SepaFlash Silica Flash Column, eluent of 0–25% EtOAc/petroleum ether gradient at 100 mL/min) to afford *tert*-butyl (*R,E*)-(1-(difluoromethoxy)-4-(methylsulfonyl)­but-3-en-2-yl)­carbamate (300 mg, 57% yield) as a yellow oil. ^1^H NMR (400 MHz, chloroform-*d*): δ 6.91 (dd, *J* = 4.6, 15.1 Hz, 1H), 6.61 (dd, *J* = 1.6, 15.2 Hz, 1H), 6.25 (t, *J*_HF_ = 73.2 Hz, 1H), 4.94 (br d, *J* = 8.3 Hz, 1H), 4.69 (br s, 1H), 4.03 (d, *J* = 4.1 Hz, 2H), 2.97 (s, 3H), 1.52–1.46 (m, 9H). *tert*-Butyl (*R,E*)-(1-(difluoromethoxy)-4-(methylsulfonyl)­but-3-en-2-yl)­carbamate (300 mg, 0.95 mmol) was used for General Procedure III to afford the title compound (150 mg, 41% yield) as a white solid. ^1^H NMR (400 MHz, methanol-*d*_4_): δ 7.70 (d, *J* = 8.3 Hz, 2H), 7.23 (d, *J* = 8.0 Hz, 2H), 7.05 (dd, *J* = 1.3, 15.5 Hz, 1H), 6.84 (dd, *J* = 6.4, 15.5 Hz, 1H), 6.54 (t, *J*_HF_ = 73.4 Hz, 1H), 4.42 (q, *J* = 5.3 Hz, 1H), 4.28–4.22 (m, 1H), 4.18–4.11 (m, 1H), 3.04 (s, 3H), 2.37 (s, 3H).

### General Procedure IV: Suzuki Cross-Coupling of Aryl Halide

(dppf)­PdCl_2_ (0.1 equiv) was added to a suspension of the appropriate aryl bromide or chloride (1 equiv), cyclopent-1-en-1-ylboronic acid (1.3 equiv), and potassium carbonate (2 equiv) in 1,4-dioxane and water (5:1, 0.25 M) at room temperature. The mixture was heated at 100 °C for 12 h under an atmosphere of dry N_2_. After cooling to room temperature, the mixture was diluted with water and extracted with EtOAc (3 times). The combined organic layers were dried over anhydrous Na_2_SO_4_, filtered, and concentrated under reduced pressure to give a residue. The residue was purified as described to afford the corresponding cyclopentene.

**3:**
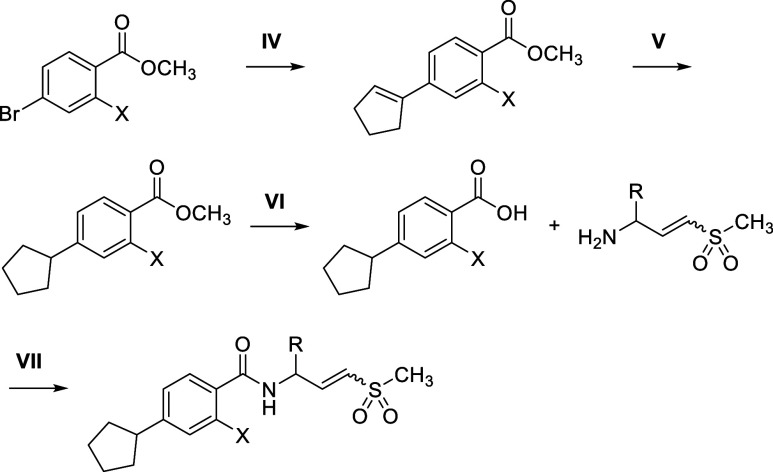
Synthesis of Benzamide Analogs

### General Procedure V: Hydrogenation of Olefin

A suspension of the appropriate cyclopentene (1 equiv) and Pd/C (10 wt %) in MeOH (0.22 M) was stirred at room temperature for 12 h under an atmosphere of hydrogen gas (balloon, 15 psi). The mixture was filtered and rinsed with MeOH (3 times). The filtrate was concentrated under reduced pressure to give the corresponding pentane, which was used without further purification unless otherwise noted.

### General Procedure VI: Saponification of Alkyl Ester

A mixture of the appropriate methyl or ethyl ester (1 equiv) and lithium hydroxide (5 equiv) in THF, MeOH, and water (2:2:1, 0.1 M) was stirred at room temperature for 12 h. The mixture was washed with water and acidified with 1 N HCl to pH 2, then the aqueous layer was extracted with EtOAc (3 times). The combined organic layers were washed with brine, dried over MgSO_4_, filtered, and concentrated to afford the crude carboxylic acid, which was used in subsequent reactions without further purification.

### General Procedure VII: Amide Coupling Using HATU

HATU (1.5 equiv) was added to a solution of the appropriate carboxylic acid (1 equiv), the appropriate amine (1.0–1.3 equiv), and DIPEA (2 equiv) in DCM (0.10 M) at 0 °C. After 15 min, the mixture was allowed to warm to room temperature and stirred for 2 h under an atmosphere of dry N_2_. The reaction mixture was poured into water and extracted with DCM (3 times). The combined organic layers were washed with brine, dried over anhydrous Na_2_SO_4_, filtered, and concentrated under reduced pressure to give a residue. The residue was purified as described to afford the corresponding amide.

#### (*E*)-4-Cyclopentyl-*N*-(3-(methylsulfonyl)­allyl)­benzamide (**1a**)

Methyl 4-bromobenzoate (300 mg, 1.40 mmol) was used for General Procedure IV and purified by flash silica gel chromatography (ISCO; 4g SepaFlash Silica Flash Column, eluent of 0–3% EtOAc/petroleum ether gradient at 80 mL/min) to afford methyl 4-(cyclopent-1-en-1-yl)­benzoate (280 mg, 99% yield) as a white solid. MS (ESI^+^): *m*/*z* 203.2 [M + H]^+^. ^1^H NMR (400 MHz, chloroform-*d*): δ 7.98 (d, *J* = 8.4 Hz, 2H), 7.49 (d, *J* = 8.4 Hz, 2H), 6.35 (t, *J* = 2.0 Hz, 1H), 3.92 (s, 3H), 2.78–2.70 (m, 2H), 2.57 (ddd, *J* = 2.4, 5.1, 9.8 Hz, 2H), 2.11–2.00 (m, 2H). Methyl 4-(cyclopent-1-en-1-yl)­benzoate (280 mg, 1.38 mmol) was used for General Procedure V to afford methyl 4-cyclopentylbenzoate (270 mg, 95% yield) as a white solid. MS (ESI^+^): *m*/*z* 205.1 [M + H]^+^. Methyl 4-cyclopentylbenzoate (270 mg, 1.32 mmol) was used for General Procedure VI to afford 4-cyclopentylbenzoic acid (230 mg, 91% yield) as a white solid. MS (ESI^+^): *m*/*z* 191.1 [M + H]^+^. 4-Cyclopentylbenzoic acid (50 mg, 0.26 mmol) and (*E*)-3-(methylsulfonyl)­prop-2-en-1-amine tosylate salt (96.9 mg, 0.32 mmol) were used for General Procedure VII and purified by prep-HPLC (Nanomicro Kromasil C18, 100 × 30 mm, 5 μm, 20–30% ACN in water (0.1% TFA)) to afford the title compound (56.1 mg, 69% yield) as a white solid. Purity = 100%. MS (ESI^+^): *m*/*z* 308.8 [M + H]^+^. ^1^H NMR (400 MHz, DMSO-*d*_6_): δ 8.77 (br t, *J* = 5.6 Hz, 1H), 7.82 (d, *J* = 8.2 Hz, 2H), 7.35 (d, *J* = 8.0 Hz, 2H), 6.80 (s, 1H), 6.75–6.67 (m, 1H), 4.13 (br t, *J* = 4.0 Hz, 2H), 3.09–3.02 (m, 1H), 3.01 (s, 3H), 2.09–1.97 (m, 2H), 1.85–1.72 (m, 2H), 1.72–1.61 (m, 1H), 1.66 (br dd, *J* = 4.8, 7.6 Hz, 1H), 1.60–1.49 (m, 2H).

#### (*E*)-4-Cyclopentyl-2-methoxy-*N*-(3-(methylsulfonyl)­allyl)­benzamide (**1b**)

Methyl 4-bromo-2-methoxybenzoate (5.0 g, 20.4 mmol) was used for General Procedure IV and purified by flash silica gel chromatography (ISCO; 80g SepaFlash Silica Flash Column, eluent of 0–100% EtOAc/petroleum ether gradient at 50 mL/min) to afford methyl 4-(cyclopent-1-en-1-yl)-2-methoxybenzoate (4.1 g, 87% yield) as a white solid. MS (ESI^+^): *m*/*z* 233.1 [M + H]^+^. ^1^H NMR (400 MHz, chloroform-*d*): δ 7.77 (d, *J* = 8.2 Hz, 1H), 7.04 (dd, *J* = 1.4, 8.0 Hz, 1H), 7.01 (s, 1H), 6.31 (quint, *J* = 2.2 Hz, 1H), 3.93 (s, 3H), 3.88 (s, 3H), 2.77–2.68 (m, 2H), 2.56 (qt, *J* = 2.4, 7.4 Hz, 2H), 2.04 (quint, *J* = 7.5 Hz, 2H). Methyl 4-(cyclopent-1-en-1-yl)-2-methoxybenzoate (4.1 g, 17.65 mmol) was used for General Procedure V to afford methyl 4-cyclopentyl-2-methoxybenzoate (4.1 g, 100% yield) as a yellow oil. ^1^H NMR (400 MHz, chloroform-*d*): δ 7.74 (d, *J* = 8.0 Hz, 1H), 6.90–6.80 (m, 2H), 3.90 (s, 3H), 3.87 (s, 3H), 3.12–2.90 (m, 1H), 2.15–2.00 (m, 2H), 1.91–1.50 (m, 6H). Methyl 4-cyclopentyl-2-methoxybenzoate (4.1 g, 17.7 mmol) was used for General Procedure VI to afford 4-cyclopentyl-2-methoxybenzoic acid (3.7 g, 95% yield) as a yellow solid. ^1^H NMR (400 MHz, DMSO-*d*_6_): δ 12.36 (s, 1H), 7.58 (d, *J* = 7.9 Hz, 1H), 6.96 (d, *J* = 1.1 Hz, 1H), 6.87 (dd, *J* = 1.2, 8.0 Hz, 1H), 3.81 (s, 3H), 3.08–2.92 (m, 1H), 2.07–1.96 (m, 2H), 1.88–1.47 (m, 6H). 4-Cyclopentyl-2-methoxybenzoic acid (86.0 mg, 0.39 mmol) and (*E*)-3-(methylsulfonyl)­prop-2-en-1-amine tosylate salt (120 mg, 0.39 mmol) were used for General Procedure VII and purified by prep-HPLC (Nanomicro Kromasil C18, 100 × 30 mm, 5 μm, 35–55% ACN in water (0.1% TFA)) to afford the title compound (55.9 mg, 42% yield) as a white solid. Purity = 99.8%. MS (ESI^+^): *m*/*z* 338.1 [M + H]^+^. ^1^H NMR (400 MHz, chloroform-*d*): δ 8.12 (d, *J* = 8.0 Hz, 2H), 7.10–6.96 (m, 2H), 6.88 (s, 1H), 6.52 (td, *J* = 2.0, 15.1 Hz, 1H), 4.40–4.31 (m, 2H), 4.01 (s, 3H), 3.12–2.99 (m, 1H), 2.94 (s, 3H), 2.17–2.06 (m, 2H), 1.90–1.79 (m, 2H), 1.78–1.68 (m, 2H), 1.67–1.57 (m, 2H).

#### (*E*)-2-Cyclobutoxy-4-cyclopentyl-*N*-(3-(methylsulfonyl)­allyl)­benzamide (**1c**)

A suspension of methyl 4-bromo-2-hydroxybenzoate (500 mg, 2.16 mmol, 1 equiv), bromocyclobutane (584.3 mg, 4.33 mmol, 2 equiv), potassium carbonate (598.2 mg, 4.33 mmol, 2 equiv), and sodium iodide (32.4 mg, 0.22 mmol, 0.1 equiv) in DMF (10 mL) was heated at 80 °C for 2 h. After cooling to room temperature, the mixture was diluted with water (30 mL), extracted with EtOAc (25 mL × 3). The combined organic layers were washed with brine (25 mL × 2), dried over anhydrous Na_2_SO_4_, filtered, and concentrated under reduced pressure to give a residue. The residue was purified by flash silica gel chromatography (ISCO; 4g SepaFlash Silica Flash Column, eluent of 0–50% EtOAc/petroleum ether gradient at 45 mL/min) to afford methyl 4-bromo-2-cyclobutoxybenzoate (500 mg, 81% yield) as a yellow oil. MS (ESI^+^): *m*/*z* 285.0, 287.0 [M + H]^+^. Methyl 4-bromo-2-cyclobutoxybenzoate (130 mg, 0.46 mmol) was used for General Procedure IV and purified by prep-TLC (SiO_2_ 400–500 mesh, EtOAc/petroleum ether = 1:1) to afford methyl 2-cyclobutoxy-4-(cyclopent-1-en-1-yl)­benzoate (45 mg, 36% yield) as a white solid. MS (ESI^+^): *m*/*z* 273.0 [M + H]^+^. ^1^H NMR (400 MHz, DMSO-*d*_6_): δ 7.63 (d, *J* = 8.1 Hz, 1H), 7.13–7.05 (m, 1H), 6.97–6.86 (m, 1H), 6.56–6.37 (m, 1H), 4.82 (quint, *J* = 7.2 Hz, 1H), 3.82–3.67 (m, 3H), 2.73–2.62 (m, 2H), 2.46–2.39 (m, 3H), 2.14–1.91 (m, 4H), 1.86–1.58 (m, 2H). Methyl 2-cyclobutoxy-4-(cyclopent-1-en-1-yl)­benzoate (45 mg, 0.17 mmol) was used for General Procedure V to afford methyl 2-cyclobutoxy-4-cyclopentylbenzoate (45 mg, 100% yield) as a yellow solid. MS (ESI^+^): *m*/*z* 275.1 [M + H]^+^. ^1^H NMR (400 MHz, DMSO-*d*_6_): δ 7.62–7.53 (m, 1H), 6.91–6.84 (m, 1H), 6.81–6.72 (m, 1H), 4.83–4.68 (m, 1H), 3.80–3.70 (m, 3H), 3.08–2.93 (m, 1H), 2.47–2.34 (m, 2H), 2.13–1.92 (m, 5H), 1.85–1.42 (m, 9H). Methyl 2-cyclobutoxy-4-cyclopentylbenzoate (45 mg, 0.17 mmol) was used for General Procedure VI to afford 2-cyclobutoxy-4-cyclopentylbenzoic acid, which was directly used for General Procedure VII along with (*E*)-3-(methylsulfonyl)­prop-2-en-1-amine tosylate salt (106.3 mg, 0.35 mmol) and purified by prep-HPLC (Nanomicro Kromasil C18, 100 × 30 mm, 5 μm, 33–63% ACN in water (0.075% TFA)) to afford the title compound (32 mg, 49% yield in two steps) as a yellow solid. Purity = 97.9%. MS (ESI^+^): *m*/*z* 378.2 [M + H]^+^. ^1^H NMR (400 MHz, DMSO-*d*_6_): δ 8.39–8.20 (m, 1H), 7.70–7.58 (m, 1H), 6.95–6.66 (m, 4H), 4.92–4.76 (m, 1H), 4.23–4.10 (m, 2H), 3.07–2.94 (m, 4H), 2.48–2.40 (m, 2H), 2.25–2.11 (m, 2H), 2.07–1.96 (m, 2H), 1.87–1.47 (m, 8H).

#### (*E*)-4-Cyclopentyl-*N*-(3-(methylsulfonyl)­allyl)-2-phenoxybenzamide (**1d**)

A solution of methyl 4-bromo-2-fluorobenzoate (500 mg, 2.15 mmol, 1 equiv) and phenol (240 mg, 2.55 mmol, 1.2 equiv) in ACN (10 mL, 0.21 M) was heated at 80 °C for 12 h. The mixture was filtered and concentrated under reduced pressure to afford crude methyl 4-bromo-2-phenoxybenzoate (425 mg, 64% yield) as a colorless oil, which was used in the subsequent reaction without further purification. MS (ESI^+^): *m*/*z* 307.0, 309.0 [M + H]^+^. The crude methyl 4-bromo-2-phenoxybenzoate (425 mg, 1.38 mmol) was used for General Procedure IV and purified by flash silica gel chromatography (Biotage; 20g Agela Flash Silica Flash Column, eluent of 0–19% EtOAc/petroleum ether) to afford methyl 4-(cyclopent-1-en-1-yl)-2-phenoxybenzoate (400 mg, 98% yield) as a yellow solid. MS (ESI^+^): *m*/*z* 295.3 [M + H]^+^. ^1^H NMR (400 MHz, chloroform-*d*): δ 7.95–7.85 (m, 1H), 7.35–7.27 (m, 2H), 7.27–7.24 (m, 1H), 7.09–7.04 (m, 1H), 6.95 (dd, *J* = 0.9, 8.7 Hz, 1H), 6.38–6.24 (m, 1H), 3.97–3.70 (m, 3H), 2.75–2.49 (m, 4H), 2.03 (quint d, *J* = 7.5, 18.4 Hz, 2H). Methyl 4-(cyclopent-1-en-1-yl)-2-phenoxybenzoate (400 mg, 1.36 mmol) was used for General Procedure V and purified by prep-TLC (SiO_2_ 400–500 mesh, EtOAc/petroleum ether = 1:5) to afford methyl 4-cyclopentyl-2-phenoxybenzoate (235 mg, 58% yield) as a white solid. MS (ESI^+^): *m*/*z* 297.1 [M + H]^+^. ^1^H NMR (400 MHz, chloroform-*d*): δ 7.87 (d, *J* = 8.1 Hz, 1H), 7.35–7.28 (m, 2H), 7.11–7.04 (m, 2H), 6.94 (dd, *J* = 0.8, 8.6 Hz, 2H), 6.89 (d, *J* = 1.3 Hz, 1H), 3.77 (s, 3H), 3.03–2.91 (m, 1H), 2.08–2.01 (m, 2H), 1.81–1.75 (m, 2H), 1.73–1.62 (m, 2H), 1.56–1.47 (m, 2H). Methyl 4-cyclopentyl-2-phenoxybenzoate was used for General Procedure VI to afford 4-cyclopentyl-2-phenoxybenzoic acid (60 mg, 78% yield) as a white solid, which was used for General Procedure VII along with (*E*)-3-(methylsulfonyl)­prop-2-en-1-amine tosylate salt (84.8 mg, 0.28 mmol) and purified by prep-TLC (SiO_2_ 400–500 mesh, EtOAc) to afford the title compound (44.1 mg, 52% yield) as a yellow solid. Purity = 100%. MS (ESI^+^): *m*/*z* 400.1 [M + H]^+^. ^1^H NMR (400 MHz, chloroform-*d*): δ 8.17–8.13 (m, 1H), 7.88 (br t, *J* = 5.3 Hz, 1H), 7.45–7.41 (m, 2H), 7.23 (t, *J* = 7.4 Hz, 1H), 7.13 (dd, *J* = 1.3, 8.2 Hz, 1H), 7.08–7.05 (m, 2H), 6.96 (td, *J* = 4.1, 15.1 Hz, 1H), 6.74–6.70 (m, 1H), 6.34 (td, *J* = 2.0, 15.1 Hz, 1H), 4.32 (ddd, *J* = 2.0, 4.1, 6.0 Hz, 2H), 2.98–2.89 (m, 1H), 2.83 (s, 3H), 2.04–1.98 (m, 2H), 1.78–1.72 (m, 2H), 1.70–1.64 (m, 2H), 1.55–1.46 (m, 2H).

### Synthetic Procedure for Pyrimidine Analogs

(*E*)-2-Cyclopentyl-4-methoxy-*N*-(3-(methylsulfonyl)­allyl)­pyrimidine-5-carboxamide (**1e**). Sodium methoxide (391 mg, 7.25 mmol, 1.5 equiv) was added to a solution of methyl 2,4-dichloropyrimidine-5-carboxylate (1.0 g, 4.83 mol, 1 equiv) in MeOH (10 mL, 0.48 M) at room temperature. After stirring for 16 h, the mixture was diluted with water (20 mL) and extracted with EtOAc (20 mL × 2). The combined organic layers were dried over anhydrous Na_2_SO_4_, filtered, and concentrated under reduced pressure to give a residue. The residue was purified by flash silica gel chromatography (SiO_2_ 230–400 mesh, eluent of 30% EtOAc/petroleum ether) to afford methyl 2-chloro-4-methoxypyrimidine-5-carboxylate (220 mg, 22% yield) as a colorless oil. Methyl 2-chloro-4-methoxypyrimidine-5-carboxylate (140 mg, 0.69 mmol) was used for General Procedure IV and purified by prep-TLC (SiO_2_ 400–500 mesh, EtOAc/petroleum ether = 1:3) to afford methyl 2-(cyclopent-1-en-1-yl)-4-methoxypyrimidine-5-carboxylate (120 mg, 74% yield) as a yellow solid. MS (ESI^+^): *m*/*z* 235.1 [M + H]^+^. ^1^H NMR (400 MHz, chloroform-*d*): δ 8.81–9.18 (m, 1H), 7.06–7.21 (m, 1H), 4.08–4.20 (m, 3H), 3.83–3.99 (m, 3H), 2.75–3.01 (m, 2H), 2.52–2.71 (m, 2H), 1.97–2.11 (m, 2H). Methyl 2-(cyclopent-1-en-1-yl)-4-methoxypyrimidine-5-carboxylate (120 mg, 0.51 mmol) was used for General Procedure V and purified by prep-TLC (SiO_2_ 400–500 mesh, EtOAc/petroleum ether = 1:3) to afford methyl 2-cyclopentyl-4-methoxypyrimidine-5-carboxylate (100 mg, 83% yield) as a white solid. ^1^H NMR (400 MHz, chloroform-*d*): δ 8.92 (s, 1H), 4.09 (s, 3H), 3.91 (s, 4H), 3.32 (br t, 1H), 2.09 (br d, 2H), 1.90–1.99 (m, 2H), 1.83 (br d, 2H), 1.68–1.75 (m, 2H). Methyl 2-cyclopentyl-4-methoxypyrimidine-5-carboxylate (100 mg, 0.42 mmol) was used for General Procedure VI to afford 2-cyclopentyl-4-methoxypyrimidine-5-carboxylic acid (90 mg, 96% yield) as a white solid. ^1^H NMR (400 MHz, chloroform-*d*): δ 9.13 (s, 1H), 4.19 (s, 3H), 3.38 (t, 1H), 2.06–2.16 (m, 2H), 1.81–2.01 (m, 4H), 1.73 (td, 2H). 2-Cyclopentyl-4-methoxypyrimidine-5-carboxylic acid (90 mg, 0.41 mmol) and (*E*)-3-(methylsulfonyl)­prop-2-en-1-amine tosylate salt (149 mg, 0.49 mmol) were used for General Procedure VII and purified by prep-TLC (SiO_2_ 400–500 mesh, EtOAc/petroleum ether = 1:1) to afford the title compound (40.5 mg, 29% yield) as a white solid. Purity = 98.8%. MS (ESI^+^): *m*/*z* 340.0 [M + H]^+^. ^1^H NMR (400 MHz, chloroform-*d*): δ 9.14–9.30 (m, 1H), 7.77–7.90 (m, 1H), 6.89–7.01 (m, 1H), 6.52 (br d, *J* = 15.13 Hz, 1H), 4.22–4.42 (m, 5H), 3.37–3.58 (m, 1H), 2.80–3.03 (m, 3H), 2.09–2.31 (m, 2H), 1.82–2.03 (m, 4H), 1.76 (br dd, *J* = 6.75, 4.75 Hz, 2H).

### General Procedure VIII: Pinner Pyrimidine Synthesis

Diethyl ethoxymethylenemalonate (1 equiv) was added to a solution of the appropriate amidine (1 equiv) and sodium ethoxide (5 equiv) in EtOH (1.5 M) at room temperature. The mixture was heated at 90 °C for 2 h under an atmosphere of dry N_2_. After cooling to room temperature, the mixture was concentrated under reduced pressure to give a residue. The residue was partitioned between water and DCM. The combined organic layers were dried over anhydrous Na_2_SO_4_, filtered, and concentrated under reduced pressure to give a residue. The residue was purified as described to afford the corresponding pyrimidine.

**4:**
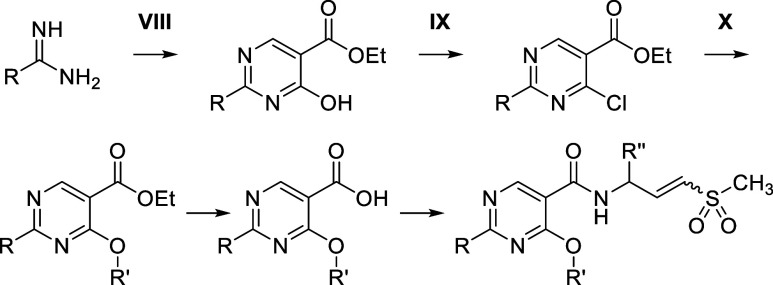
Synthesis of Pyrimidine Analogs (Except for Pyrimidines **6a**, **6d**, and **7d**)

### General Procedure IX: Chlorination of Hydroxy Pyrimidine

A solution of the appropriate 4-hydroxy pyrimidine in POCl_3_ (0.5 M) was heated at 80 °C for 3 h under an atmosphere of dry N_2_. After cooling to room temperature, the mixture was concentrated under reduced pressure to afford the corresponding 4-chloropyrimidine, which was used in subsequent reactions without further purification.

### General Procedure X: Nucleophilic Aromatic Substitution (SNAr)

A suspension of the appropriate 4-chloropyrimidine (1 equiv), the appropriate hydroxyl compound (1 equiv), and potassium carbonate (2.5 equiv) in ACN (0.6 M) was heated at 80 °C for 12 h under an atmosphere of dry N_2_. After cooling to room temperature, the reaction mixture was concentrated under reduced pressure to give a residue. The residue was partitioned between water and EtOAc. The combined organic layers were dried over anhydrous Na_2_SO_4_, filtered, and concentrated under reduced pressure to give a residue. The residue was purified as described to afford the corresponding ether.

#### (*E*)-2-Cyclopentyl-*N*-(3-(methylsulfonyl)­allyl)-4-phenoxypyrimidine-5-carboxamide (**1f**)

A mixture of cyclopentanecarbonitrile (5.0 g, 52.6 mmol, 1 equiv) and HCl in MeOH (75 mL, 3M) was stirred at room temperature for 12 h in a sealed vessel. The mixture was concentrated under reduced pressure to afford methyl cyclopentanecarbimidate (6.6 g, 99% yield) as a white solid. ^1^H NMR (400 MHz, methanol-*d*_4_): δ 5.50 (s, 1H), 4.21–4.13 (m, 3H), 3.16–3.03 (m, 1H), 2.15–2.01 (m, 2H), 1.94–1.77 (m, 5H), 1.75–1.67 (m, 2H). A mixture of the crude methyl cyclopentanecarbimidate (6.6 g, 51.9 mmol, 1 equiv) and ammonia in MeOH (20 mL, 7N) was stirred at room temperature for 12 h in a sealed vessel. The mixture was concentrated under reduced pressure to afford cyclopentanecarboxamidine (5.5 g, 94% yield) as a light yellow solid, which was used in the subsequent reaction without further purification. ^1^H NMR (400 MHz, methanol-*d*_4_): δ 2.92–2.79 (m, 1H), 2.64 (d, *J* = 9.4 Hz, 1H), 2.16–2.05 (m, 2H), 1.92–1.81 (m, 2H), 1.78–1.65 (m, 4H). The crude cyclopentanecarboxamidine (5.5 g, 49.0 mmol) was used for General Procedure VIII and purified by flash silica gel chromatography (Biotage; 80g Agela Flash Silica Flash Column, eluent of 0–100% EtOAc/petroleum ether) to afford ethyl 2-cyclopentyl-4-hydroxypyrimidine-5-carboxylate (2.3 g, 20% yield) as a light yellow solid. MS (ESI^+^): *m*/*z* 237.1 [M + H]^+^. ^1^H NMR (400 MHz, methanol-*d*_4_): δ 8.60 (s, 1H), 4.32 (q, *J* = 7.0 Hz, 2H), 3.17–2.98 (m, 1H), 2.11–2.01 (m, 2H), 1.92–1.79 (m, 4H), 1.75–1.66 (m, 2H), 1.35 (t, *J* = 7.1 Hz, 3H). Ethyl 2-cyclopentyl-4-hydroxypyrimidine-5-carboxylate (2.3 g, 9.7 mmol) was used for General Procedure IX to afford crude ethyl 4-chloro-2-cyclopentylpyrimidine-5-carboxylate (2.0 g, 81% yield) as a brown oil. ^1^H NMR (400 MHz, chloroform-*d*): δ 9.11 (s, 1H), 4.49–4.41 (m, 2H), 3.47 (quint, *J* = 8.2 Hz, 1H), 2.22–2.11 (m, 2H), 1.98–1.86 (m, 4H), 1.80–1.65 (m, 3H), 1.43 (t, *J* = 7.1 Hz, 3H). Ethyl 4-chloro-2-cyclopentylpyrimidine-5-carboxylate (1.5 g, 5.89 mmol) and phenol (0.55 g, 5.89 mmol) were used for General Procedure X and purified by prep-HPLC (Welch Xtimate C18, 100 × 25 mm, 3 μm, 35–65% ACN in water (0.04% HCl)) to afford ethyl 2-cyclopentyl-4-phenoxypyrimidine-5-carboxylate (1.5 g, 82% yield) as a yellow solid. MS (ESI^+^): *m*/*z* 313.1 [M + H]^+^. ^1^H NMR (400 MHz, chloroform-*d*): δ 9.08–9.00 (m, 1H), 7.45–7.37 (m, 2H), 7.26–7.14 (m, 1H), 7.19–7.14 (m, 2H), 6.93–6.82 (m, 1H), 4.43 (q, *J* = 7.1 Hz, 2H), 3.21 (quint, *J* = 7.8 Hz, 1H), 2.00–1.89 (m, 2H), 1.76–1.66 (m, 2H), 1.64–1.51 (m, 4H), 1.41 (t, *J* = 7.1 Hz, 3H). Ethyl 2-cyclopentyl-4-phenoxypyrimidine-5-carboxylate (700 mg, 2.24 mmol) was used for General Procedure VI to afford 2-cyclopentyl-4-phenoxypyrimidine-5-carboxylic acid (450 mg, 71% yield) as a yellow solid. MS (ESI^+^): *m*/*z* 285.1 [M + H]^+^. ^1^H NMR (400 MHz, chloroform-*d*): δ 9.23 (s, 1H), 7.50–7.38 (m, 2H), 7.33–7.28 (m, 1H), 7.18 (d, *J* = 7.7 Hz, 2H), 3.27 (quint, *J* = 7.9 Hz, 1H), 1.96 (br dd, *J* = 7.0, 11.7 Hz, 2H), 1.75–1.66 (m, 2H), 1.65–1.54 (m, 4H). 2-Cyclopentyl-4-phenoxypyrimidine-5-carboxylic acid (100 mg, 0.35 mmol) and (*E*)-3-(methylsulfonyl)­prop-2-en-1-amine tosylate salt (114 mg, 0.37 mmol) were used for General Procedure VII and purified by prep-HPLC (Welch Xtimate C18, 100 × 25 mm, 3 μm, 25–65% ACN in water (0.04% HCl)) to afford the title compound (34.3 mg, 24% yield) as a white solid. Purity = 98.7%. MS (ESI^+^): *m*/*z* 402.1 [M + H]^+^. ^1^H NMR (400 MHz, chloroform-*d*): δ 9.32 (s, 1H), 7.83 (br s, 1H), 7.53–7.44 (m, 2H), 7.39–7.32 (m, 1H), 7.18 (d, *J* = 7.8 Hz, 2H), 7.02 (td, *J* = 4.5, 15.2 Hz, 1H), 6.56 (br d, *J* = 15.1 Hz, 1H), 4.38 (br t, *J* = 4.3 Hz, 2H), 3.24 (quint, *J* = 7.7 Hz, 1H), 2.96 (s, 3H), 2.00–1.89 (m, 2H), 1.73–1.65 (m, 2H), 1.57 (br d, *J* = 5.0 Hz, 4H).

#### (*S*,*E*)-2-Cyclopentyl-*N*-(4-(methylsulfonyl)­but-3-en-2-yl)-4-phenoxypyrimidine-5-Carboxamide **(2a)**

2-Cyclopentyl-4-phenoxypyrimidine-5-carboxylic acid (100 mg, 0.35 mmol) and (*S,E*)-4-(methylsulfonyl)­but-3-en-2-amine tosylate salt (119 mg, 0.37 mmol) were used for General Procedure VII and purified by prep-HPLC (Welch Xtimate C18, 100 × 25 mm, 3 μm, 25–65% ACN in water (0.04% HCl)) to afford the title compound (91.9 mg, 63% yield) as a yellow solid. Purity = 99.8%, e.e. = 98.2%. MS (ESI^+^): *m*/*z* 416.1 [M + H]^+^. HR-MS (ESI^+^): *m*/*z* calcd for [C_21_H_25_N_3_O_4_S + H]^+^: 416.1639; found, 416.1644. ^1^H NMR (400 MHz, chloroform-*d*): δ 9.29 (s, 1H), 7.73 (br d, *J* = 7.3 Hz, 1H), 7.55–7.47 (m, 2H), 7.41–7.34 (m, 1H), 7.21 (d, *J* = 7.6 Hz, 2H), 6.95 (dd, *J* = 4.9, 15.1 Hz, 1H), 6.59 (dd, *J* = 1.3, 15.2 Hz, 1H), 5.10–4.99 (m, 1H), 3.41 (quint, *J* = 7.6 Hz, 1H), 2.96 (s, 3H), 2.01 (br dd, *J* = 3.5, 8.5 Hz, 2H), 1.68 (ddd, *J* = 3.7, 8.2, 15.7 Hz, 2H), 1.63–1.55 (m, 4H), 1.48 (d, *J* = 7.1 Hz, 3H). ^1^H NMR (400 MHz, DMSO-*d*_6_): δ 8.82 (s, 1H), 8.66 (d, *J* = 8.0 Hz, 1H), 7.47 (dd, *J* = 8.9, 7.0 Hz, 2H), 7.33–7.25 (m, 3H), 6.83 (d, *J* = 2.9 Hz, 2H), 4.85 (m, 1H), 3.12 (p, *J* = 7.8 Hz, 1H), 2.96 (s, 3H), 1.92–1.81 (m, 2H), 1.72–1.62 (m, 2H), 1.59–1.48 (m, 4H), 1.32 (d, *J* = 7.1 Hz, 3H). ^13^C NMR (101 MHz, DMSO-*d*_6_): δ 175.13, 164.86, 162.20, 158.59, 151.82, 146.80, 129.57, 129.44, 125.57, 121.85, 114.58, 47.51, 45.25, 42.18, 32.15, 32.13, 25.30, 19.25. LC–MS report, SFC report, and NMR spectra for compound **2a** are included in Figure S6.

#### (*R*,*E*)-2-Cyclopentyl-*N*-(4-(methylsulfonyl)­but-3-en-2-yl)-4-phenoxypyrimidine-5-Carboxamide **(2b)**

2-Cyclopentyl-4-phenoxypyrimidine-5-carboxylic acid (100 mg, 0.35 mmol) and (*R,E*)-4-(methylsulfonyl)­but-3-en-2-amine tosylate salt (119 mg, 0.37 mmol) were used for General Procedure VII and purified by prep-HPLC (Welch Xtimate C18, 100 × 25 mm, 3 μm, 25–65% ACN in water (0.04% HCl)) to afford the title compound (97.4 mg, 66% yield) as a yellow solid. Purity = 99.8%, e.e. = 95.8%. MS (ESI^+^): *m*/*z* 416.1 [M + H]^+^. ^1^H NMR (400 MHz, chloroform-*d*): δ 9.30 (s, 1H), 7.67 (br d, *J* = 7.3 Hz, 1H), 7.54–7.47 (m, 2H), 7.40–7.35 (m, 1H), 7.20 (d, *J* = 7.8 Hz, 2H), 6.95 (dd, *J* = 4.9, 15.1 Hz, 1H), 6.57 (d, *J* = 15.3 Hz, 1H), 5.11–4.99 (m, 1H), 3.36 (quint, *J* = 7.7 Hz, 1H), 2.95 (s, 3H), 2.03–1.94 (m, 2H), 1.75–1.64 (m, 2H), 1.63–1.55 (m, 4H), 1.47 (d, *J* = 7.0 Hz, 3H).

#### (*S*,*Z*)-2-Cyclopentyl-*N*-(4-(methylsulfonyl)­but-3-en-2-yl)-4-phenoxypyrimidine-5-Carboxamide **(2c)**

2-Cyclopentyl-4-phenoxypyrimidine-5-carboxylic acid (67.5 mg, 0.24 mmol) and (*S,Z*)-4-(methylsulfonyl)­but-3-en-2-amine tosylate salt (80.1 mg, 0.25 mmol) were used for General Procedure VII and purified by prep-HPLC (Waters Xbridge Prep OBD C18, 150 × 40 mm, 10 μm, 40–70% ACN in water (10 mM NH_4_HCO_3_)) to afford the title compound (58.1 mg, 59% yield) as a yellow solid. Purity = 100%, e.e. = 100%. MS (ESI^+^): *m*/*z* 416.2 [M + H]^+^. ^1^H NMR (400 MHz, chloroform-*d*): δ 9.26 (s, 1H), 7.67 (br d, *J* = 6.1 Hz, 1H), 7.52–7.45 (m, 2H), 7.38–7.31 (m, 1H), 7.19 (d, *J* = 7.8 Hz, 2H), 6.38–6.32 (m, 1H), 6.29–6.21 (m, 1H), 5.65–5.54 (m, 1H), 3.30 (s, 3H), 3.22 (quint, *J* = 7.7 Hz, 1H), 1.98–1.89 (m, 2H), 1.74–1.64 (m, 2H), 1.63–1.56 (m, 4H), 1.48 (d, *J* = 7.0 Hz, 3H).

#### (*R*,*Z*)-2-Cyclopentyl-*N*-(4-(methylsulfonyl)­but-3-en-2-yl)-4-phenoxypyrimidine-5-Carboxamide **(2d)**

2-Cyclopentyl-4-phenoxypyrimidine-5-carboxylic acid (67.5 mg, 0.24 mmol) and (*R,Z*)-4-(methylsulfonyl)­but-3-en-2-amine tosylate salt (80.2 mg, 0.25 mmol) were used for General Procedure VII and purified by prep-HPLC (Waters Xbridge Prep OBD C18, 150 × 40 mm, 10 μm, 40–70% ACN in water (10 mM NH_4_HCO_3_)) to afford the title compound (45.7 mg, 46% yield) as a yellow solid. Purity = 100%, e.e. = 100%. MS (ESI^+^): *m*/*z* 416.2 [M + H]^+^. ^1^H NMR (400 MHz, chloroform-*d*): δ 9.26 (s, 1H), 7.67 (br d, *J* = 5.8 Hz, 1H), 7.52–7.45 (m, 2H), 7.39–7.32 (m, 1H), 7.19 (d, *J* = 7.6 Hz, 2H), 6.39–6.33 (m, 1H), 6.29–6.21 (m, 1H), 5.65–5.55 (m, 1H), 3.30 (s, 3H), 3.22 (quint, *J* = 7.8 Hz, 1H), 1.93 (br dd, *J* = 6.9, 12.1 Hz, 2H), 1.74–1.64 (m, 2H), 1.63–1.56 (m, 4H), 1.48 (d, *J* = 6.9 Hz, 3H), 1.35 (br d, *J* = 6.3 Hz, 1H), 1.27 (br dd, *J* = 7.0, 16.0 Hz, 1H).

#### 2-Cyclopentyl-*N*-(3-(methylsulfonyl)­propyl)-4-phenoxypyrimidine-5-Carboxamide **(2e)**

2-Cyclopentyl-4-phenoxypyrimidine-5-carboxylic acid (200 mg, 0.49 mmol) and 3-(methylsulfonyl)­propan-1-amine tosylate salt (160 mg, 0.52 mmol) were used for General Procedure VII and purified by prep-HPLC (Phenomenex Luna C18, 100 × 40 mm, 5 μm, 20–65% ACN in water (0.04% HCl)) to afford the title compound (61.7 mg, 31% yield) as a white solid. Purity = 99.8%. MS (ESI^+^): *m*/*z* 404.1 [M + H]^+^. ^1^H NMR (400 MHz, chloroform-*d*): δ 9.29 (s, 1H), 7.81 (br t, *J* = 5.4 Hz, 1H), 7.52–7.43 (m, 2H), 7.38–7.31 (m, 1H), 7.22–7.14 (m, 2H), 3.75–3.66 (m, 2H), 3.31–3.20 (m, 1H), 3.19–3.11 (m, 2H), 2.92 (s, 3H), 2.29–2.17 (m, 2H), 2.03–1.87 (m, 2H), 1.74–1.64 (m, 2H), 1.63–1.51 (m, 4H).

#### (*S*,*E*)-2-Cyclopentyl-*N*-Methyl-*N*-(4-(methylsulfonyl)­but-3-en-2-yl)-4-phenoxypyrimidine-5-Carboxamide **(3f)**

2-Cyclopentyl-4-phenoxypyrimidine-5-carboxylic acid (1.0 g, 3.52 mmol, 1 equiv) and commercial ethyl methyl- *l* -alaninate (1.18 g, 7.03 mmol, 2 equiv) were used for General Procedure VII and purified by flash silica gel chromatography (SiO_2_ 230–400 mesh, eluent of 0–50% EtOAc/petroleum ether) to afford ethyl *N*-(2-cyclopentyl-4-phenoxypyrimidine-5-carbonyl)-*N*-methyl- *l* -alaninate (0.81 g, 58% yield) as a yellow oil. ^1^H NMR (400 MHz, chloroform-*d*): δ 8.58 (s, 1H), 7.41 (dd, *J* = 8.3, 3.7 Hz, 2H), 7.23 (s, 1H), 7.15 (d, *J* = 8.0 Hz, 2H), 4.41 (q, *J* = 7.0 Hz, 1H), 4.21 (q, *J* = 6.8 Hz, 2H), 3.17 (d, *J* = 8.0 Hz, 1H), 3.02 (s, 3H), 1.73 (s, 2H), 1.66 (s, 2H), 1.61 (d, *J* = 7.4 Hz, 4H), 1.51 (s, 3H), 1.24 (d, *J* = 7.1 Hz, 3H). Sodium borohydride (35.7 mg, 0.94 mmol, 1.5 equiv) was added to a solution of ethyl *N*-(2-cyclopentyl-4-phenoxypyrimidine-5-carbonyl)-*N*-methyl-l-alaninate (250 mg, 0.63 mmol, 1 equiv) in EtOH (5 mL, 0.13 M) at 0 °C. The mixture was allowed to warm to room temperature and stirred for 12 h. The mixture was quenched with 1 N HClaq (5 mL) at 0 °C. The residue was diluted with water (5 mL) and extracted with DCM (10 mL × 3). The combined organic layers were washed with brine (10 mL × 2), dried over anhydrous Na_2_SO_4_, filtered, and concentrated under reduced pressure to afford crude (*S*)-2-cyclopentyl-*N*-(1-hydroxypropan-2-yl)-*N*-methyl-4-phenoxypyrimidine-5-carboxamide (150 mg, 67% yield), which was used in the subsequent reaction without further purification. ^1^H NMR (400 MHz, methanol-*d*_4_): δ 8.50 (s, 1H), 7.45 (t, *J* = 8.0 Hz, 2H), 7.29 (t, *J* = 7.4 Hz, 1H), 7.22–7.14 (m, 2H), 3.87 (s, 1H), 3.74–3.63 (m, 1H), 3.47 (s, 3H), 3.19 (q, *J* = 7.8 Hz, 1H), 3.00–2.93 (m, 1H), 1.97–1.88 (m, 2H), 1.72 (tdd, *J* = 7.7, 5.3, 2.5 Hz, 2H), 1.61 (dddd, *J* = 10.8, 9.3, 7.1, 4.3 Hz, 4H), 1.23 (dd, *J* = 6.8, 1.5 Hz, 3H). Dess–Martin periodinane (358 mg, 0.84 mmol, 2 equiv) was added to a solution of (*S*)-2-cyclopentyl-*N*-(1-hydroxypropan-2-yl)-*N*-methyl-4-phenoxypyrimidine-5-carboxamide (150 mg, 0.42 mmol, 1 equiv) in DCM (5 mL, 0.08 M) at 0 °C. The mixture was allowed to warm to room temperature and stirred for 12 h. The mixture was filtered, and the filter cake was rinsed with DCM (10 mL × 3). The combined organic filtrates were concentrated under reduced pressure to provide crude (*S*)-2-cyclopentyl-*N*-methyl-*N*-(1-oxopropan-2-yl)-4-phenoxypyrimidine-5-carboxamide (120 mg, 80% yield) as a colorless oil, which was used in the subsequent reaction without further purification. ^1^H NMR (400 MHz, chloroform-*d*): δ 9.66 (s, 1H), 8.60 (d, *J* = 10.6 Hz, 1H), 7.44–7.40 (m, 2H), 7.30–7.27 (m, 1H), 7.17–7.13 (m, 2H), 4.71 (q, *J* = 7.1 Hz, 1H), 3.20 (td, *J* = 7.8, 4.2 Hz, 1H), 3.06 (s, 3H), 1.75–1.70 (m, 2H), 1.67–1.63 (m, 2H), 1.62–1.54 (m, 4H), 1.49 (d, *J* = 7.1 Hz, 3H). The crude (*S*)-2-cyclopentyl-*N*-methyl-*N*-(1-oxopropan-2-yl)-4-phenoxypyrimidine-5-carboxamide (120 mg, 0.34 mmol) was used for General Procedure II and purified by prep-HPLC (Waters Xbridge BEH C18, 100 × 25 mm, 5 μm, 35–65% ACN in water (10 mM NH_4_HCO_3_)) to afford the title compound (19 mg, 13% yield) as a pale yellow solid. Purity = 100%. MS (ESI^+^): *m*/*z* 430.2 [M + H]^+^. ^1^H NMR (400 MHz, DMSO-*d*_6_): δ 8.70–8.62 (m, 1H), 7.49–7.40 (m, 2H), 7.33–7.13 (m, 3H), 6.89–6.61 (m, 2H), 5.47–4.60 (m, 1H), 3.18–3.05 (m, 2H), 3.03–2.78 (m, 6H), 1.96–1.79 (m, 2H), 1.76–1.62 (m, 2H), 1.62–1.48 (m, 4H), 1.43–1.27 (m, 3H).

#### (*S*,*E*)-4-(Cyclohexyloxy)-2-Cyclopentyl-*N*-(4-(methylsulfonyl)­but-3-en-2-yl)­pyrimidine-5-Carboxamide (**4a**)

DIAD (0.31 g, 1.2 equiv) was added dropwise to a solution of ethyl 2-cyclopentyl-4-hydroxypyrimidine-5-carboxylate (300 mg, 1.27 mmol, 1 equiv) and cyclohexanol (127 mg, 1.27 mmol, 1 equiv) in THF (6 mL, 0.21 M) at 0 °C. The mixture was heated at 40 °C for 2 h. After cooling to room temperature, the mixture was partitioned between water (5 mL) and EtOAc (5 mL). The aqueous phase was extracted with EtOAc (5 mL × 2), and the combined organic layers were dried over anhydrous Na_2_SO_4_, filtered and concentrated under reduced pressure to give a residue. The residue was purified by prep-TLC (SiO_2_ 400–500 mesh, EtOAc/petroleum ether = 10:1) to afford ethyl 4-(cyclohexyloxy)-2-cyclopentylpyrimidine-5-carboxylate (100 mg, 25% yield) as a yellow oil. ^1^H NMR (400 MHz, chloroform-*d*): δ 8.89 (s, 1H), 5.38–5.26 (m, 1H), 4.36 (q, *J* = 7.1 Hz, 2H), 3.35–3.20 (m, 1H), 2.12–2.02 (m, 2H), 1.98–1.86 (m, 4H), 1.85–1.77 (m, 4H), 1.75–1.64 (m, 4H), 1.59–1.51 (m, 1H), 1.51–1.41 (m, 3H), 1.38 (t, *J* = 7.1 Hz, 3H). Ethyl 4-(cyclohexyloxy)-2-cyclopentylpyrimidine-5-carboxylate (100 mg, 0.31 mmol) was used for General Procedure VI to afford 4-(cyclohexyloxy)-2-cyclopentylpyrimidine-5-carboxylic acid (90 mg, 99% yield) as a white solid. ^1^H NMR (400 MHz, chloroform-*d*): δ 9.15 (s, 1H), 5.53–5.41 (m, 1H), 3.50–3.37 (m, 1H), 2.19–2.02 (m, 4H), 1.96–1.88 (m, 2H), 1.86–1.78 (m, 4H), 1.77–1.68 (m, 4H), 1.66–1.58 (m, 1H), 1.56–1.38 (m, 3H). 4-(Cyclohexyloxy)-2-cyclopentylpyrimidine-5-carboxylic acid (80 mg, 0.28 mmol) and (*S,E*)-4-(methylsulfonyl)­but-3-en-2-amine tosylate salt (88.6 mg, 0.28 mmol) were used for General Procedure VII and purified by prep-HPLC (Waters Xbridge Prep OBD C18, 150 × 40 mm, 10 μm, 50–80% ACN in water (10 mM NH_4_HCO_3_)) to afford the title compound (33.7 mg, 29% yield) as a yellow oil. Purity = 100%, e.e. = 96.9%. MS (ESI^+^): *m*/*z* 422.2 [M + H]^+^. ^1^H NMR (400 MHz, chloroform-*d*): δ 9.22–9.10 (m, 1H), 7.80 (br d, *J* = 7.6 Hz, 1H), 6.94 (dd, *J* = 4.8, 15.1 Hz, 1H), 6.50 (dd, *J* = 1.6, 15.1 Hz, 1H), 5.50–5.39 (m, 1H), 5.07–4.90 (m, 1H), 3.42–3.27 (m, 1H), 2.95 (s, 3H), 2.16–2.03 (m, 4H), 1.95–1.80 (m, 4H), 1.78–1.69 (m, 4H), 1.65 (td, *J* = 4.3, 8.6 Hz, 2H), 1.61–1.50 (m, 4H), 1.44 (d, *J* = 7.1 Hz, 3H).

#### (*S*,*E*)-2-Cyclopentyl-*N*-(4-(methylsulfonyl)­but-3-en-2-yl)-4-((tetrahydro-2*H*-pyran-4-yl)­oxy)­pyrimidine-5-Carboxamide **(4b)**

Ethyl 4-chloro-2-cyclopentylpyrimidine-5-carboxylate (500 mg, 1.96 mmol) and tetrahydro-2*H*-pyran-4-ol (200 mg, 1.96 mmol) were used for General Procedure X while potassium *tert*-butoxide (1.77 mL, 1 M in THF, 1.77 mmol) was used as the base and purified by flash silica gel chromatography (ISCO; 12g SepaFlash Silica Flash Column, eluent of 5–10% EtOAc/*n*-hexane) to afford ethyl 2-cyclopentyl-4-((tetrahydro-2*H*-pyran-4-yl)­oxy)­pyrimidine-5-carboxylate (275 mg, 44% yield) as a colorless oil. MS (ESI^+^): *m*/*z* 321.1 [M + H]^+^. ^1^H NMR (400 MHz, chloroform-*d*): δ 8.93 (s, 1H), 5.52 (dt, *J* = 7.16, 3.49 Hz, 1H), 4.38 (q, *J* = 7.13 Hz, 2H), 4.00 (ddd, *J* = 11.38, 7.44, 3.56 Hz, 2H), 3.68 (ddd, *J* = 11.35, 7.29, 3.63 Hz, 2H), 3.25–3.37 (m, 1H), 2.04–2.14 (m, 4H), 1.80–1.93 (m, 6H), 1.67–1.75 (m, 2H), 1.39 (t, *J* = 7.13 Hz, 3H). Ethyl 2-cyclopentyl-4-((tetrahydro-2*H*-pyran-4-yl)­oxy)­pyrimidine-5-carboxylate (275 mg, 0.86 mmol) was used for General Procedure VI to afford 2-cyclopentyl-4-((tetrahydro-2*H*-pyran-4-yl)­oxy)­pyrimidine-5-carboxylic acid (230 mg, 92% yield) as a white solid. MS (ESI^+^): *m*/*z* 293.2 [M + H]^+^. ^1^H NMR (400 MHz, chloroform-*d*): δ 9.15 (s, 1H), 5.62 (dt, *J* = 8.32, 4.22 Hz, 1H), 3.95–4.06 (m, 2H), 3.67 (ddd, *J* = 11.85, 8.66, 3.13 Hz, 2H), 3.30–3.41 (m, 1H), 2.14–2.21 (m, 2H), 2.06–2.14 (m, 2H), 1.82–1.98 (m, 6H), 1.70–1.76 (m, 2H). 2-Cyclopentyl-4-((tetrahydro-2*H*-pyran-4-yl)­oxy)­pyrimidine-5-carboxylic acid (230 mg, 0.55 mmol) and (*S,E*)-4-(methylsulfonyl)­but-3-en-2-amine tosylate salt (186 mg, 0.58 mmol) were used for General Procedure VII and purified by prep-HPLC (Phenomenex Luna C18, 100 × 40 mm, 5 μm, 30–60% ACN in water (0.04% HCl)) to afford the title compound (64.5 mg, 27% yield) as a pink solid. Purity = 98.3%, e.e. = 99.1%. MS (ESI^+^): *m*/*z* 424.1 [M + H]^+^. ^1^H NMR (400 MHz, chloroform-*d*): δ 9.17 (br s, 1H), 7.68 (br d, *J* = 4.63 Hz, 1H), 6.92 (br dd, *J* = 14.13, 3.75 Hz, 1H), 6.54 (br d, *J* = 14.76 Hz, 1H), 5.63 (br s, 1H), 4.92–5.03 (m, 1H), 3.99 (br d, *J* = 4.38 Hz, 2H), 3.64–3.74 (m, 2H), 3.53 (br d, *J* = 1.88 Hz, 1H), 2.96 (s, 3H), 2.07–2.30 (m, 4H), 1.71–1.98 (m, 8H), 1.47 (br d, *J* = 6.75 Hz, 3H).

#### (*S*,*E*)-4-(4-Chlorophenoxy)-2-Cyclopentyl-*N*-(4-(methylsulfonyl)­but-3-en-2-yl)­pyrimidine-5-Carboxamide (**4c**)

Ethyl 4-chloro-2-cyclopentylpyrimidine-5-carboxylate (300 mg, 1.18 mmol) and 4-chlorophenol (151 mg, 1.18 mmol) were used for General Procedure X and purified by prep-TLC (SiO_2_ 400–500 mesh, EtOAc/petroleum ether = 1:1) to afford ethyl 4-(4-chlorophenoxy)-2-cyclopentylpyrimidine-5-carboxylate (220 mg, 54% yield) as a light yellow oil. MS (ESI^+^): *m*/*z* 427.0 [M + H]^+^. ^1^H NMR (400 MHz, chloroform-*d*): δ 9.05 (s, 1H), 7.40–7.36 (m, 2H), 7.14–7.09 (m, 2H), 4.43 (q, *J* = 7.0 Hz, 2H), 3.23 (t, *J* = 7.8 Hz, 1H), 2.01–1.92 (m, 2H), 1.73–1.57 (m, 6H), 1.41 (t, *J* = 7.1 Hz, 3H). Ethyl 4-(4-chlorophenoxy)-2-cyclopentylpyrimidine-5-carboxylate (220 mg, 0.63 mmol) was used for General Procedure VI to afford 4-(4-chlorophenoxy)-2-cyclopentylpyrimidine-5-carboxylic acid (170 mg, 84% yield) as a light yellow solid. MS (ESI^+^): *m*/*z* 319.0 [M + H]^+^. 4-(4-Chlorophenoxy)-2-cyclopentylpyrimidine-5-carboxylic acid (90 mg, 0.28 mmol) and (*S,E*)-4-(methylsulfonyl)­but-3-en-2-amine tosylate salt (95 mg 0.30 mmol) were used for General Procedure VII and purified by prep-HPLC (Waters Xbridge Prep OBD C18, 150 × 40 mm, 10 μm, 40–65% ACN in water (10 mM NH_4_HCO_3_)) to afford the title compound (71 mg, 56% yield) as a yellow solid. Purity = 100%, e.e. = 96.7%. MS (ESI^+^): *m*/*z* 450.1 [M + H]^+^. ^1^H NMR (400 MHz, chloroform-*d*): δ 9.30 (s, 1H), 7.46 (d, *J* = 8.8 Hz, 3H), 7.14 (d, *J* = 8.8 Hz, 2H), 6.95 (dd, *J* = 4.9, 15.1 Hz, 1H), 6.58–6.50 (m, 1H), 5.10–4.99 (m, 1H), 3.29–3.20 (m, 1H), 2.96 (s, 3H), 2.01–1.93 (m, 2H), 1.73–1.67 (m, 2H), 1.66–1.57 (m, 4H), 1.46 (d, *J* = 7.0 Hz, 3H).

#### (*S*,*E*)-4-(3-Chlorophenoxy)-2-Cyclopentyl-*N*-(4-(methylsulfonyl)­but-3-en-2-yl)­pyrimidine-5-Carboxamide **(4d)**

Ethyl 4-chloro-2-cyclopentylpyrimidine-5-carboxylate (600 mg, 2.36 mmol) and 3-chlorophenol (333 mg, 2.59 mmol) were used for General Procedure X to afford crude ethyl 4-(3-chlorophenoxy)-2-cyclopentylpyrimidine-5-carboxylate (640 mg, 78% yield) as a brown oil, which was used in the subsequent reaction without further purification. MS (ESI^+^): *m*/*z* 347.0 [M + H]^+^. The crude ethyl 4-(3-chlorophenoxy)-2-cyclopentylpyrimidine-5-carboxylate (300 mg, 0.87 mmol) was used for General Procedure VI to afford 4-(3-chlorophenoxy)-2-cyclopentylpyrimidine-5-carboxylic acid (180 mg, 65% yield) as a colorless oil. MS (ESI^+^): *m*/*z* 319.0 [M + H]^+^. ^1^H NMR (400 MHz, chloroform-*d*): δ 9.23 (s, 1H), 7.40–7.35 (m, 1H), 7.31–7.27 (m, 1H), 7.25 (t, *J* = 2.1 Hz, 1H), 7.13–7.08 (m, 1H), 3.29 (quint, *J* = 7.8 Hz, 1H), 2.05–1.94 (m, 2H), 1.76–1.58 (m, 6H). 4-(3-Chlorophenoxy)-2-cyclopentylpyrimidine-5-carboxylic acid (70 mg, 0.22 mmol) and (*S,E*)-4-(methylsulfonyl)­but-3-en-2-amine tosylate salt (74 mg, 0.23 mmol) were used for General Procedure VII and purified by prep-HPLC (Waters Xbridge BEH C18, 100 × 25 mm, 5 μm, 50–85% ACN in water (10 mM NH_4_HCO_3_)) to afford the title compound (46.9 mg, 47% yield) as a yellow solid. Purity = 100%. MS (ESI^+^): *m*/*z* 450.1 [M + H]^+^. ^1^H NMR (400 MHz, chloroform-*d*): δ 9.30 (s, 1H), 7.43 (t, *J* = 8.1 Hz, 2H), 7.37–7.32 (m, 1H), 7.25 (t, *J* = 2.0 Hz, 1H), 7.10 (dd, *J* = 1.4, 8.1 Hz, 1H), 6.95 (dd, *J* = 5.0, 15.1 Hz, 1H), 6.54 (dd, *J* = 1.3, 15.2 Hz, 1H), 5.10–4.99 (m, 1H), 3.25 (quint, *J* = 7.7 Hz, 1H), 2.96 (s, 3H), 2.04–1.91 (m, 2H), 1.75–1.67 (m, 2H), 1.66–1.57 (m, 5H), 1.47 (d, *J* = 7.1 Hz, 3H).

#### (*S*,*E*)-4-(2-Chlorophenoxy)-2-Cyclopentyl-*N*-(4-(methylsulfonyl)­but-3-en-2-yl)­pyrimidine-5-Carboxamide **(4e)**

Ethyl 4-chloro-2-cyclopentylpyrimidine-5-carboxylate (320 mg, 1.26 mmol) and 2-chlorophenol (178 mg, 1.38 mmol) were used for General Procedure X and purified by prep-TLC (SiO_2_ 400–500 mesh, EtOAc/petroleum ether = 1:5) to afford ethyl 4-(2-chlorophenoxy)-2-cyclopentylpyrimidine-5-carboxylate (240 mg, 55% yield) as a brown oil. MS (ESI^+^): *m*/*z* 347.1 [M + H]^+^. ^1^H NMR (400 MHz, chloroform-*d*): δ 9.07 (br s, 1H), 7.50–7.44 (m, 1H), 7.36–7.30 (m, 1H), 7.26–7.19 (m, 2H), 4.44 (q, *J* = 7.1 Hz, 2H), 3.28–3.13 (m, 1H), 1.96–1.85 (m, 2H), 1.67 (br dd, *J* = 6.6, 11.3 Hz, 2H), 1.60–1.49 (m, 4H), 1.42 (t, *J* = 7.1 Hz, 3H). Ethyl 4-(2-chlorophenoxy)-2-cyclopentylpyrimidine-5-carboxylate (100 mg, 0.29 mmol) was used for General Procedure VI to afford 4-(2-chlorophenoxy)-2-cyclopentylpyrimidine-5-carboxylic acid (92 mg, 100% yield) as a yellow oil. MS (ESI^+^): *m*/*z* 319.1 [M + H]^+^. ^1^H NMR (400 MHz, chloroform-*d*): δ 9.23 (s, 1H), 7.49–7.45 (m, 1H), 7.33 (dd, *J* = 1.6, 7.8 Hz, 1H), 7.27 (s, 1H), 7.23 (s, 1H), 3.26 (quint, *J* = 7.7 Hz, 1H), 1.92 (br dd, *J* = 8.1, 12.7 Hz, 2H), 1.72–1.59 (m, 2H), 1.57–1.47 (m, 4H). 4-(2-Chlorophenoxy)-2-cyclopentylpyrimidine-5-carboxylic acid (92 mg, 0.29 mmol) and (*S,E*)-4-(methylsulfonyl)­but-3-en-2-amine tosylate salt (106 mg, 0.33 mmol) were used for General Procedure VII and purified by prep-HPLC (Waters Xbridge BEH C18, 100 × 25 mm, 5 μm, 50–85% ACN in water (10 mM NH_4_HCO_3_)) to afford the title compound (79.7 mg, 61% yield) as a white solid. Purity = 100%. MS (ESI^+^): *m*/*z* 450.1 [M + H]^+^. ^1^H NMR (400 MHz, chloroform-*d*): δ 9.31 (s, 1H), 7.62 (br d, *J* = 7.7 Hz, 1H), 7.54 (dd, *J* = 1.3, 7.9 Hz, 1H), 7.44–7.29 (m, 3H), 6.97 (dd, *J* = 4.7, 15.1 Hz, 1H), 6.56 (dd, *J* = 1.7, 15.1 Hz, 1H), 5.12–5.02 (m, 1H), 3.24 (quint, *J* = 7.8 Hz, 1H), 2.95 (s, 3H), 2.01–1.89 (m, 2H), 1.74–1.64 (m, 2H), 1.61–1.54 (m, 4H), 1.47 (d, *J* = 7.1 Hz, 3H).

#### (*S*,*E*)-2-Cyclopentyl-4-(3-fluorophenoxy)-*N*-(4-(methylsulfonyl)­but-3-en-2-yl)­pyrimidine-5-Carboxamide (**4f**)

Ethyl 4-chloro-2-cyclopentylpyrimidine-5-carboxylate (400 mg, 1.57 mmol) and 3-fluorophenol (211 mg, 1.88 mmol) were used for General Procedure X to afford crude ethyl 2-cyclopentyl-4-(3-fluorophenoxy)­pyrimidine-5-carboxylate (300 mg, 58% yield) as a brown oil, which was used in the subsequent reaction without further purification. MS (ESI^+^): *m*/*z* 331.0 [M + H]^+^. The crude ethyl 2-cyclopentyl-4-(3-fluorophenoxy)­pyrimidine-5-carboxylate (200 mg, 0.61 mmol) was used for General Procedure VI to afford 2-cyclopentyl-4-(3-fluorophenoxy)­pyrimidine-5-carboxylic acid (80 mg, 44% yield) as a white solid. ^1^H NMR (400 MHz, chloroform-*d*): δ 9.21 (s, 1H), 7.40 (td, *J* = 8.3, 6.4 Hz, 1H), 7.86 (s, 1H), 7.07–6.93 (m, 3H), 3.27 (p, *J* = 7.8 Hz, 1H), 2.04–1.92 (m, 2H), 1.73 (t, *J* = 6.5 Hz, 2H), 1.67–1.58 (m, 4H). 2-Cyclopentyl-4-(3-fluorophenoxy)­pyrimidine-5-carboxylic acid (80 mg, 0.26 mmol) and (*S,E*)-4-(methylsulfonyl)­but-3-en-2-amine tosylate salt (89.3 mg, 0.28 mmol) were used for General Procedure VII and purified by prep-HPLC (Phenomenex Gemini-NX C18, 75 × 30 mm, 3 μm, 30–70% ACN in water (10 mM NH_4_HCO_3_)) to afford the title compound (33 mg, 29% yield) as a yellow solid. Purity = 99.7%, e.e. = 95.0%. MS (ESI^+^): *m*/*z* 434.1 [M + H]^+^. ^1^H NMR (400 MHz, chloroform-*d*): δ 9.31 (s, 1H), 7.49–7.40 (m, 2H), 7.13–7.04 (m, 1H), 7.02–6.91 (m, 3H), 6.54 (dd, *J* = 1.4, 15.2 Hz, 1H), 5.10–4.97 (m, 1H), 3.26 (br t, *J* = 7.6 Hz, 1H), 2.96 (s, 3H), 2.04–1.90 (m, 2H), 1.71 (br dd, *J* = 2.4, 7.4 Hz, 2H), 1.65–1.58 (m, 4H), 1.47 (d, *J* = 7.0 Hz, 3H).

#### (*S*,*E*)-2-Cyclopentyl-*N*-(4-(methylsulfonyl)­but-3-en-2-yl)-4-(*m*-tolyloxy)­pyrimidine-5-Carboxamide (**4g**)

Ethyl 4-chloro-2-cyclopentylpyrimidine-5-carboxylate (1.0 g, 2.36 mmol) and *m*-cresol (255 mg, 2.36 mmol) were used for General Procedure X and purified by flash silica gel chromatography (ISCO; 4g SepaFlash Silica Flash Column, eluent of 0–4% EtOAc/petroleum ether gradient at 100 mL/min) to afford ethyl 2-cyclopentyl-4-(*m*-tolyloxy)­pyrimidine-5-carboxylate (750 mg, 98% yield) as a white solid. MS (ESI^+^): *m*/*z* 327.1 [M + H]^+^. ^1^H NMR (400 MHz, chloroform-*d*): δ 9.03 (s, 1H), 7.32–7.28 (m, 1H), 7.12–6.87 (m, 3H), 4.49–4.37 (m, 2H), 3.22 (t, *J* = 7.8 Hz, 1H), 2.39 (s, 3H), 2.00–1.88 (m, 2H), 1.80–1.67 (m, 2H), 1.67–1.60 (m, 2H), 1.59–1.56 (m, 2H), 1.41 (t, *J* = 7.1 Hz, 3H). Ethyl 2-cyclopentyl-4-(*m*-tolyloxy)­pyrimidine-5-carboxylate (400 mg, 1.23 mmol) was used for General Procedure VI to afford 2-cyclopentyl-4-(*m*-tolyloxy)­pyrimidine-5-carboxylic acid (220 mg, 60% yield) as a white solid. MS (ESI^+^): *m*/*z* 299.2 [M + H]^+^. ^1^H NMR (400 MHz, chloroform-*d*): δ 9.11 (s, 1H), 7.25–7.17 (m, 1H), 7.03 (br d, *J* = 7.0 Hz, 1H), 6.97–6.89 (m, 2H), 3.19 (br t, *J* = 7.6 Hz, 1H), 2.38–2.30 (m, 3H), 1.96–1.77 (m, 4H), 1.70 (br d, *J* = 5.5 Hz, 2H), 1.57 (br d, *J* = 6.5 Hz, 2H). 2-Cyclopentyl-4-(*m*-tolyloxy)­pyrimidine-5-carboxylic acid (220 mg, 0.52 mmol) and (*S,E*)-4-(methylsulfonyl)­but-3-en-2-amine tosylate salt (174 mg, 0.54 mmol) were used for General Procedure VII and purified by prep-HPLC (Waters Xbridge BEH C18, 100 × 25 mm, 5 μm, 50–85% ACN in water (10 mM NH_4_HCO_3_)) to afford the title compound (58.6 mg, 26% yield) as a pale yellow solid. Purity = 99.9%, e.e. = 100%. MS (ESI^+^): *m*/*z* 430.1 [M + H]^+^. ^1^H NMR (400 MHz, chloroform-*d*): δ 9.29 (s, 1H), 7.59 (br d, *J* = 7.6 Hz, 1H), 7.35 (t, *J* = 7.8 Hz, 1H), 7.15 (d, *J* = 7.5 Hz, 1H), 7.04–6.90 (m, 3H), 6.59–6.48 (m, 1H), 5.13–4.98 (m, 1H), 3.24 (t, *J* = 7.8 Hz, 1H), 2.95 (s, 3H), 2.43 (s, 3H), 2.00–1.88 (m, 2H), 1.77–1.66 (m, 2H), 1.65–1.53 (m, 4H), 1.46 (d, *J* = 7.1 Hz, 3H).

#### (*S*,*E*)-2-Methyl-*N*-(4-(methylsulfonyl)­but-3-en-2-yl)-4-phenoxypyrimidine-5-Carboxamide (**5a**)

Commercial ethyl 4-hydroxy-2-methylpyrimidine-5-carboxylate (1.0 g, 5.49 mmol) was used for General Procedure IX to afford crude ethyl 4-chloro-2-methylpyrimidine-5-carboxylate (1.1 g, 100% yield) as a brown oil, which was used in the subsequent reaction without further purification. The crude ethyl 4-chloro-2-methylpyrimidine-5-carboxyl (300 mg, 1.50 mmol) and phenol (169 mg, 1.79 mmol) were used for General Procedure X to afford crude ethyl 2-methyl-4-phenoxypyrimidine-5-carboxylate (386 mg, 100% yield), which was used in the subsequent reaction without further purification. MS (ESI^+^): *m*/*z* 259.1 [M + H]^+^. ^1^H NMR (400 MHz, chloroform-*d*): δ 9.03 (s, 1H), 7.46–7.40 (m, 2H), 7.26–7.22 (m, 1H), 7.17 (d, *J* = 7.5 Hz, 2H), 4.43 (q, *J* = 7.1 Hz, 2H), 2.55 (s, 3H), 1.42 (t, *J* = 7.1 Hz, 3H). The crude ethyl 2-methyl-4-phenoxypyrimidine-5-carboxylate (200 mg, 0.77 mmol) was used for General Procedure VI to afford 2-methyl-4-phenoxypyrimidine-5-carboxylic acid (140 mg, 79% yield) as a yellow oil. MS (ESI^+^): *m*/*z* 231.1 [M + H]^+^. ^1^H NMR (400 MHz, chloroform-*d*): δ 9.22 (s, 1H), 7.48–7.42 (m, 2H), 7.34–7.28 (m, 1H), 7.19 (d, *J* = 7.7 Hz, 2H), 2.60 (s, 3H). 2-Methyl-4-phenoxypyrimidine-5-carboxylic acid (59.6 mg, 0.26 mmol) and (*S,E*)-4-(methylsulfonyl)­but-3-en-2-amine tosylate salt (80 mg, 0.25 mmol) were used for General Procedure VII and purified by prep-HPLC (Waters Xbridge BEH C18, 100 × 25 mm, 5 μm, 15–45% ACN in water (10 mM NH_4_HCO_3_)) to afford the title compound (44.5 mg, 48% yield) as a white solid. Purity = 100%. MS (ESI^+^): *m*/*z* 362.1 [M + H]^+^. ^1^H NMR (400 MHz, chloroform-*d*): δ 9.29 (s, 1H), 7.54–7.46 (m, 2H), 7.40–7.34 (m, 1H), 7.19 (d, *J* = 7.7 Hz, 2H), 6.95 (dd, *J* = 4.9, 15.2 Hz, 1H), 6.54 (dd, *J* = 1.6, 15.0 Hz, 1H), 5.09–4.99 (m, 1H), 2.95 (s, 3H), 2.57 (s, 3H), 1.46 (d, *J* = 7.1 Hz, 3H).

#### (*S*,*E*)-2-Ethyl-*N*-(4-(methylsulfonyl)­but-3-en-2-yl)-4-phenoxypyrimidine-5-Carboxamide (**5b**)

Propanamidine hydrochloride salt (2.0 g, 18.4 mmol) was used for General Procedure VIII to afford crude ethyl 2-ethyl-4-hydroxypyrimidine-5-carboxylate (1.2 g, 33% yield) as a white solid. MS (ESI^+^): *m*/*z* 197.1 [M + H]^+^. The crude ethyl 2-ethyl-4-hydroxypyrimidine-5-carboxylate (500 mg, 2.55 mmol) was used for General Procedure IX to afford crude ethyl 4-chloro-2-ethylpyrimidine-5-carboxylate (547 mg, 100% yield) as a brown oil, which was used without further purification. The crude ethyl 4-chloro-2-ethylpyrimidine-5-carboxylate (547 mg, 2.55 mmol) and phenol (242 mg, 2.57 mmol) were used for General Procedure X to afford ethyl 2-ethyl-4-phenoxypyrimidine-5-carboxylate (640 mg, 92% yield) as a brown oil. MS (ESI^+^): *m*/*z* 273.2 [M + H]^+^. ^1^H NMR (400 MHz, chloroform-*d*): δ 9.05 (s, 1H), 7.46–7.39 (m, 2H), 7.26–7.22 (m, 1H), 7.21–7.15 (m, 2H), 4.43 (q, *J* = 7.1 Hz, 2H), 2.81 (q, *J* = 7.6 Hz, 2H), 1.42 (t, *J* = 7.1 Hz, 3H), 1.19 (t, *J* = 7.6 Hz, 3H). Ethyl 2-ethyl-4-phenoxypyrimidine-5-carboxylate (100 mg, 0.37 mmol) was used for General Procedure VI to afford 2-ethyl-4-phenoxypyrimidine-5-carboxylic acid (85 mg, 95% yield) as a white solid. . MS (ESI^+^): *m*/*z* 245.0 [M + H]^+^. ^1^H NMR (400 MHz, chloroform-*d*): δ 9.25 (s, 1H), 7.49–7.42 (m, 2H), 7.34–7.28 (m, 1H), 7.22–7.17 (m, 2H), 2.87 (q, *J* = 7.6 Hz, 2H), 1.20 (t, *J* = 7.5 Hz, 3H). 2-Ethyl-4-phenoxypyrimidine-5-carboxylic acid (63.2 mg, 0.26 mmol) and (*S,E*)-4-(methylsulfonyl)­but-3-en-2-amine tosylate salt (87.3 mg, 0.27 mmol) were used for General Procedure VII and purified by prep-HPLC (Waters Xbridge BEH C18, 100 × 25 mm, 5 μm, 30–65% ACN in water (10 mM NH_4_HCO_3_)) to afford the title compound (56.7 mg, 58% yield) as a pale yellow solid. Purity = 100%. MS (ESI^+^): *m*/*z* 376.1 [M + H]^+^. ^1^H NMR (400 MHz, chloroform-*d*): δ 9.31 (s, 1H), 7.58 (br d, *J* = 7.5 Hz, 1H), 7.53–7.45 (m, 2H), 7.39–7.33 (m, 1H), 7.20 (d, *J* = 7.7 Hz, 2H), 6.96 (dd, *J* = 4.9, 15.2 Hz, 1H), 6.54 (dd, *J* = 1.5, 15.2 Hz, 1H), 5.10–4.99 (m, 1H), 2.96 (s, 3H), 2.84 (q, *J* = 7.5 Hz, 2H), 1.47 (d, *J* = 7.1 Hz, 3H), 1.19 (t, *J* = 7.6 Hz, 3H).

#### (*S*,*E*)-2-Isopropyl-*N*-(4-(methylsulfonyl)­but-3-en-2-yl)-4-phenoxypyrimidine-5-Carboxamide (**5c**)

2-Methylpropanamidine hydrochloride salt (2.0 g, 16.3 mmol) was used for General Procedure VIII to afford crude ethyl 4-hydroxy-2-isopropylpyrimidine-5-carboxylate (3.0 g, 88% yield) as a yellow solid, which was used in the subsequent reaction without further purification. MS (ESI^+^): *m*/*z* 211.1 [M + H]^+^. ^1^H NMR (400 MHz, methanol-*d*_4_): δ 8.52 (s, 1H), 4.27 (q, *J* = 7.1 Hz, 2H), 2.87–2.76 (m, 1H), 1.34 (t, *J* = 7.1 Hz, 3H), 1.24 (s, 3H), 1.22 (s, 3H). The crude ethyl 4-hydroxy-2-isopropylpyrimidine-5-carboxylate (1.0 g, 4.76 mmol) was used for General Procedure IX to afford crude ethyl 4-chloro-2-isopropylpyrimidine-5-carboxylate (850 mg, 78% yield) as a yellow oil, which was used in the subsequent reaction without further purification. MS (ESI^+^): *m*/*z* 229.1 [M + H]^+^. ^1^H NMR (400 MHz, chloroform-*d*): δ 9.06 (s, 1H), 4.44 (q, *J* = 7.1 Hz, 2H), 3.25 (septet, *J* = 6.9 Hz, 1H), 1.42 (t, *J* = 7.1 Hz, 3H), 1.37 (s, 3H), 1.35 (s, 3H). The crude ethyl 4-chloro-2-isopropylpyrimidine-5-carboxylate (850 mg, 3.72 mmol) and phenol (350 mg, 3.72 mmol) were used for General Procedure X to afford ethyl 2-isopropyl-4-phenoxypyrimidine-5-carboxylate (700 mg, 66% yield) as a brown oil. MS (ESI^+^): *m*/*z* 287.1 [M + H]^+^. ^1^H NMR (400 MHz, chloroform-*d*): δ 9.07 (s, 1H), 7.48–7.39 (m, 2H), 7.27 (s, 1H), 7.22–7.17 (m, 2H), 4.44 (q, *J* = 7.1 Hz, 2H), 3.04 (septet, *J* = 6.9 Hz, 1H), 1.42 (t, *J* = 7.1 Hz, 3H), 1.19 (s, 3H), 1.18 (s, 3H). Ethyl 2-isopropyl-4-phenoxypyrimidine-5-carboxylate (500 mg, 1.75 mmol) was used for General Procedure VI to afford 2-isopropyl-4-phenoxypyrimidine-5-carboxylic acid (350 mg, 78% yield) as a yellow solid. MS (ESI^+^): *m*/*z* 259.0 [M + H]^+^. ^1^H NMR (400 MHz, chloroform-*d*): δ 9.24 (s, 1H), 7.48–7.41 (m, 2H), 7.33–7.28 (m, 1H), 7.22–7.18 (m, 2H), 3.09 (td, *J* = 6.8, 13.7 Hz, 1H), 2.16–2.05 (m, 1H), 1.45–1.34 (m, 1H), 1.33–1.23 (m, 1H), 1.20 (s, 3H), 1.18 (s, 3H). 2-Isopropyl-4-phenoxypyrimidine-5-carboxylic acid (100 mg, 0.39 mmol) and (*S,E*)-4-(methylsulfonyl)­but-3-en-2-amine tosylate salt (130.7 mg, 0.41 mmol) were used for General Procedure VII and purified by prep-HPLC (Welch Xtimate C18, 100 × 25 mm, 3 μm, 25–65% ACN in water (0.04% HCl)) to afford the title compound (88 mg, 57% yield) as a yellow solid. Purity = 98.0%, e.e. = 99.3%. MS (ESI^+^): *m*/*z* 390.1 [M + H]^+^. ^1^H NMR (400 MHz, chloroform-*d*): δ 9.33 (s, 1H), 7.66 (br d, *J* = 7.1 Hz, 1H), 7.55–7.46 (m, 2H), 7.42–7.35 (m, 1H), 7.21 (d, *J* = 7.6 Hz, 2H), 6.95 (dd, *J* = 4.9, 15.2 Hz, 1H), 6.57 (dd, *J* = 1.4, 15.1 Hz, 1H), 5.10–5.00 (m, 1H), 3.22 (td, *J* = 6.8, 13.7 Hz, 1H), 2.96 (s, 3H), 1.48 (d, *J* = 7.0 Hz, 3H), 1.20 (d, *J* = 1.4 Hz, 3H), 1.19 (d, *J* = 1.4 Hz, 1H), 1.19–1.17 (m, 1H), 1.20–1.16 (m, 1H).

#### (*S*,*E*)-2-(*tert*-Butyl)-*N*-(4-(methylsulfonyl)­but-3-en-2-yl)-4-phenoxypyrimidine-5-Carboxamide (**5d**)

2,2-Dimethylpropanamidine hydrochloride salt (2.0 g, 14.6 mmol) was used for General Procedure VIII and purified by flash silica gel chromatography (ISCO; 12g SepaFlash Silica Flash Column, eluent of 0–95% EtOAc/petroleum ether gradient at 100 mL/min) to afford ethyl 2-(*tert*-butyl)-4-hydroxypyrimidine-5-carboxylate (0.80 g, 24% yield) as a brown solid. MS (ESI^+^): *m*/*z* 225.3 [M + H]^+^. Ethyl 2-(*tert*-butyl)-4-hydroxypyrimidine-5-carboxylate (600 mg, 2.68 mmol) was used for General Procedure IX to afford crude ethyl 2-(*tert*-butyl)-4-chloropyrimidine-5-carboxylate (520 mg, 80% yield) as a brown solid, which was used in the subsequent reaction without further purification. MS (ESI^+^): *m*/*z* 243.1 [M + H]^+^. Ethyl 2-(*tert*-butyl)-4-chloropyrimidine-5-carboxylate (520 mg, 2.14 mmol) and phenol (1.71 mmol) was used for General Procedure X and purified by prep-TLC (SiO_2_ 400–500 mesh, EtOAc/petroleum ether = 1:5) to afford ethyl 2-(*tert*-butyl)-4-phenoxypyrimidine-5-carboxylate (200 mg, 31% yield) as a brown oil. MS (ESI^+^): *m*/*z* 301.2 [M + H]^+^. ^1^H NMR (400 MHz, chloroform-*d*): δ 9.08 (s, 1H), 7.45–7.38 (m, 2H), 7.26–7.22 (m, 1H), 7.21–7.16 (m, 2H), 4.43 (q, *J* = 7.1 Hz, 2H), 1.41 (t, *J* = 7.1 Hz, 3H), 1.42 (br s, 1H), 1.22–1.21 (m, 1H), 1.22 (s, 9H). Ethyl 2-(*tert*-butyl)-4-phenoxypyrimidine-5-carboxylate (100 mg, 0.33 mmol) was used for General Procedure VI to afford 2-(*tert*-butyl)-4-phenoxypyrimidine-5-carboxylic acid (91 mg, 100% yield) as a white solid. MS (ESI^+^): *m*/*z* 273.1 [M + H]^+^. ^1^H NMR (400 MHz, chloroform-*d*): δ 9.26 (s, 1H), 7.47–7.40 (m, 2H), 7.31–7.28 (m, 1H), 7.22–7.18 (m, 2H), 1.28–1.20 (m, 9H). 2-(*tert*-Butyl)-4-phenoxypyrimidine-5-carboxylic acid (50 mg, 0.18 mmol) and (*S,E*)-4-(methylsulfonyl)­but-3-en-2-amine tosylate salt (62 mg, 0.19 mmol) were used for General Procedure VII and purified by prep-HPLC (Waters Xbridge BEH C18, 100 × 25 mm, 5 μm, 40–75% ACN in water (10 mM NH_4_HCO_3_)) to afford the title compound (53.8 mg, 73% yield) as a white solid. Purity = 100%, e.e. = 97.0%. MS (ESI^+^): *m*/*z* 404.2 [M + H]^+^. HR-MS (ESI^+^): *m*/*z* calcd for [C_20_H_25_N_3_O_4_S + H]^+^: 404.1639; found, 404.1643. ^1^H NMR (400 MHz, chloroform-*d*): δ 9.33 (s, 1H), 7.60 (br d, *J* = 7.6 Hz, 1H), 7.52–7.45 (m, 2H), 7.38–7.32 (m, 1H), 7.20 (d, *J* = 7.8 Hz, 2H), 6.96 (dd, *J* = 4.8, 15.1 Hz, 1H), 6.55 (dd, *J* = 1.5, 15.2 Hz, 1H), 5.09–4.99 (m, 1H), 2.96 (s, 3H), 1.47 (d, *J* = 7.1 Hz, 3H), 1.22 (s, 9H). ^1^H NMR (400 MHz, DMSO-*d*_6_): δ 8.86 (s, 1H), 8.69 (d, *J* = 8.0 Hz, 1H), 7.51–7.44 (m, 2H), 7.34–7.26 (m, 3H), 6.83 (d, *J* = 2.5 Hz, 2H), 4.85 (m, 1H), 2.97 (s, 3H), 1.33 (d, *J* = 7.1 Hz, 3H), 1.17 (s, 9H). ^13^C NMR (101 MHz, DMSO-*d*_6_): δ 177.46, 164.55, 162.22, 158.41, 151.81, 146.79, 129.57, 129.33, 125.50, 121.80, 114.41, 45.24, 42.18, 39.20, 28.92, 19.24. LC–MS report, SFC report, and NMR spectra for compound **5d** are included in Figure S2.

#### (*S*,*E*)-*N*-(4-(Methylsulfonyl)­but-3-en-2-yl)-4-Phenoxy-2-(trifluoromethyl)­pyrimidine-5-Carboxamide (**6b**)

Commercial ethyl 4-chloro-2-(trifluoromethyl)­pyrimidine-5-carboxylate (290 mg, 1.14 mmol) and phenol (127 mg, 1.37 mmol) were used for General Procedure X and purified by flash silica gel chromatography (Biotage; 4g Agela Flash Silica Flash Column, eluent of 10% EtOAc/heptanes) to afford ethyl 4-phenoxy-2-(trifluoromethyl)­pyrimidine-5-carboxylate (342 mg, 96% yield). ^1^H NMR (400 MHz, DMSO-*d*_6_): δ 9.29 (s, 1H), 7.53–7.46 (m, 2H), 7.36–7.27 (m, 3H), 4.40 (q, *J* = 7.1 Hz, 2H), 1.35 (t, *J* = 7.1 Hz, 3H). Ethyl 4-phenoxy-2-(trifluoromethyl)­pyrimidine-5-carboxylate (342 mg, 1.10 mol) was used for General Procedure VI to afford 4-phenoxy-2-(trifluoromethyl)­pyrimidine-5-carboxylic acid (282 mg, 91% yield). 4-Phenoxy-2-(trifluoromethyl)­pyrimidine-5-carboxylic acid (114 mg, 0.40 mmol) and (*S,E*)-4-(methylsulfonyl)­but-3-en-2-amine tosylate salt (168 mg, 0.52 mmol) were used for General Procedure VII and purified by prep-HPLC (Phenomenex Kinetex C18, 150 × 21.2 mm, 5 μm, 10–70% ACN in water (0.1% Formic Acid (FA))) to afford the title compound (66 mg, 40% yield) as a white solid. Purity = 100%. MS (ESI^+^): *m*/*z* 416.0 [M + H]^+^. ^1^H NMR (400 MHz, DMSO-*d*_6_): δ 9.12 (s, 1H), 8.94 (d, *J* = 8.0 Hz, 1H), 7.51 (dd, *J* = 8.9, 7.1 Hz, 2H), 7.34 (dd, *J* = 8.9, 7.1 Hz, 3H), 6.85–6.83 (m, 2H), 4.91–4.82 (m, 1H), 2.97 (s, 3H), 1.34 (d, *J* = 7.1 Hz, 3H).

#### (*S*,*E*)-2-(1,1-Difluoroethyl)-*N*-(4-(methylsulfonyl)­but-3-en-2-yl)-4-phenoxypyrimidine-5-Carboxamide (**6c**)

2-(1,1-Difluoroethyl)-4-phenoxypyrimidine-5-carboxylic acid (synthesis is described in Experimental Section, 70 mg, 0.25 mmol) and (*S,E*)-4-(methylsulfonyl)­but-3-en-2-amine tosylate salt (84.3 mg, 0.26 mmol) were used for General Procedure VII and purified by prep-HPLC (Phenomenex Luna C18, 80 × 40 mm, 3 μm, 30–60% ACN in water (10 mM NH_4_HCO_3_)) to afford the title compound (58 mg, 56% yield) as a white solid. Purity = 99.5%, e.e. = 96.3%. MS (ESI^+^): *m*/*z* 412.1 [M + H]^+^. HR-MS (ESI^+^): *m*/*z* calcd for [C_18_H_19_F_2_N_3_O_4_S + H]^+^: 412.1137; found, 412.1142. ^1^H NMR (400 MHz, chloroform-*d*): δ 9.47 (s, 1H), 7.59 (br d, *J* = 7.4 Hz, 1H), 7.54–7.48 (m, 2H), 7.41–7.35 (m, 1H), 7.22 (d, *J* = 7.6 Hz, 2H), 6.95 (dd, *J* = 5.1, 15.2 Hz, 1H), 6.55 (dd, *J* = 1.5, 15.1 Hz, 1H), 5.12–5.00 (m, 1H), 2.95 (s, 3H), 1.85 (t, *J*_HF_ = 18.5 Hz, 3H), 1.49 (d, *J* = 7.1 Hz, 3H). ^1^H NMR (400 MHz, DMSO-*d*_6_): δ 9.03 (s, 1H), 8.89 (d, *J* = 8.0 Hz, 1H), 7.54–7.45 (m, 2H), 7.36–7.29 (m, 3H), 6.84 (d, *J* = 1.5 Hz, 2H), 4.86 (m, 1H), 2.97 (s, 3H), 1.86 (t, *J*_HF_ = 19.0 Hz, 3H), 1.34 (d, *J* = 7.1 Hz, 3H). ^13^C NMR (101 MHz, DMSO-*d*_6_): δ 165.51, 161.89 (t, *J*_CF_ = 29.1 Hz), 161.47, 158.81, 151.52, 146.58, 129.67*, 126.01, 121.58, 118.90 (t, *J*_CF_ = 241.0 Hz), 118.55, 45.37, 42.18, 22.47 (t, *J*_CF_ = 26.1 Hz), 19.22 (*two peaks completely overlap). ^19^F NMR (470 MHz, DMSO-*d*_6_): δ −92.20. LC–MS report, SFC report, and NMR spectra for compound **6c** are included in Figure S3.

#### (*S*,*E*)-*N*-(1-Cyclobutyl-3-(methylsulfonyl)­allyl)-2-(1,1-Difluoroethyl)-4-phenoxypyrimidine-5-Carboxamide (**7a**)

2-(1,1-Difluoroethyl)-4-phenoxypyrimidine-5-carboxylic acid (50 mg, 0.18 mmol) and (*S,E*)-1-cyclobutyl-3-(methylsulfonyl)­prop-2-en-1-amine tosylate salt (67.7 mg, 0.19 mmol) were used for General Procedure VII and purified by prep-HPLC (Waters Xbridge BEH C18, 100 × 30 mm, 10 μm, 35–65% ACN in water (10 mM NH_4_HCO_3_)) to afford the title compound (43.4 mg, 54% yield) as a pale yellow solid. Purity = 100%, e.e. = 100%. MS (ESI^+^): *m*/*z* 452.1 [M + H]^+^. ^1^H NMR (400 MHz, methanol-*d*_4_): δ 1.76–1.87 (m, 4H), 1.91–2.04 (m, 4H), 2.08–2.15 (m, 2H), 2.59–2.70 (m, 1H), 2.93–2.94 (m, 3H), 4.56–4.60 (m, 1H), 4.82–4.84 (m, 1H), 6.81–6.84 (m, 2H), 7.26–7.37 (m, 3H), 7.45–7.52 (m, 2H), 8.98–9.01 (m, 1H).

#### (*R*,*E*)-2-(1,1-Difluoroethyl)-*N*-(1-methoxy-4-(methylsulfonyl)­but-3-en-2-yl)-4-phenoxypyrimidine-5-Carboxamide (**7b**)

2-(1,1-Difluoroethyl)-4-phenoxypyrimidine-5-carboxylic acid (50 mg, 0.18 mmol) and (*R,E*)-1-methoxy-4-(methylsulfonyl)­but-3-en-2-amine tosylate salt (62.7 mg, 0.18 mmol) were used for General Procedure VII and purified by prep-HPLC (Waters Xbridge BEH C18, 100 × 30 mm, 10 μm, 30–60% ACN in water (10 mM NH_4_HCO_3_)) to afford the title compound (36.8 mg, 47% yield) as a pale yellow solid. Purity = 100%, e.e. = 93.6%. MS (ESI^+^): *m*/*z* 442.1 [M + H]^+^. HR-MS (ESI^+^): *m*/*z* calcd for [C_19_H_21_F_2_N_3_O_5_S + H]^+^: 442.1243; found, 442.1247. ^1^H NMR (400 MHz, chloroform-*d*): δ 9.47 (s, 1H), 8.23 (br d, *J* = 7.8 Hz, 1H), 7.53–7.47 (m, 2H), 7.39–7.34 (m, 1H), 7.27–7.24 (m, 2H), 7.00 (dd, *J* = 5.4, 15.1 Hz, 1H), 6.63 (dd, *J* = 1.6, 15.1 Hz, 1H), 5.12–5.06 (m, 1H), 3.67 (d, *J* = 3.8 Hz, 2H), 3.35 (s, 3H), 2.95 (s, 3H), 1.88 (t, *J*_HF_ = 18.5 Hz, 3H). ^1^H NMR (400 MHz, DMSO-*d*_6_): δ 9.05 (s, 1H), 8.92 (d, *J* = 8.3 Hz, 1H), 7.50 (dd, *J* = 8.8, 7.1 Hz, 2H), 7.33 (d, *J* = 7.8 Hz, 3H), 6.96–6.81 (m, 2H), 5.02 (m, 1H), 3.58 (qd, *J* = 9.9, 5.5 Hz, 2H), 3.29 (s, 3H), 2.96 (s, 3H), 1.87 (t, *J*_HF_ = 19.0 Hz, 3H). ^13^C NMR (101 MHz, DMSO-*d*_6_): δ 165.54, 161.97 (t, *J*_CF_ = 29.1 Hz), 161.83, 158.96, 151.47, 143.24, 131.29, 129.66, 126.02, 121.55, 118.90 (t, *J*_CF_ = 241.1 Hz), 118.22, 72.41, 58.46, 49.55, 42.14, 22.47 (t, *J*_CF_ = 26.1 Hz). ^19^F NMR (470 MHz, DMSO-*d*_6_): δ −92.21. LC–MS report, SFC report, and NMR spectra for compound **7b** are included in Figure S4.

#### (*R*,*E*)-2-(1,1-Difluoroethyl)-*N*-(1-(difluoromethoxy)-4-(methylsulfonyl)­but-3-en-2-yl)-4-phenoxypyrimidine-5-Carboxamide (**7c**)

2-(1,1-Difluoroethyl)-4-phenoxypyrimidine-5-carboxylic acid (65 mg, 0.23 mmol) and (*R,E*)-1-(difluoromethoxy)-4-(methylsulfonyl)­but-3-en-2-amine tosylate salt (90 mg, 0.23 mmol) were used for General Procedure VII and purified by prep-HPLC (Waters Xbridge Prep OBD C18, 150 × 40 mm, 10 μm, 30–60% ACN in water (10 mM NH_4_HCO_3_)) to afford the title compound (36.3 mg, 31% yield) as a white solid. Purity = 95.2% e.e. = 99.9%. MS (ESI^+^): *m*/*z* 478.1 [M + H]^+^. ^1^H NMR (400 MHz, chloroform-*d*): δ 9.49 (s, 1H), 8.10 (br d, *J* = 7.9 Hz, 1H), 7.54–7.46 (m, 2H), 7.42–7.34 (m, 1H), 7.21 (d, *J* = 7.9 Hz, 2H), 6.99 (dd, *J* = 5.1, 15.1 Hz, 1H), 6.68 (dd, *J* = 1.6, 15.2 Hz, 1H), 6.21 (t, *J*_HF_ = 72.9 Hz, 1H), 5.33–5.23 (m, 1H), 4.26–4.13 (m, 2H), 2.97 (s, 3H), 1.86 (t, *J*_HF_ = 18.5 Hz, 3H).

#### (R)-*N*-(1-Cyclopropyl-3-(methylsulfonyl)­propyl)-2-(1,1-Difluoroethyl)-4-phenoxypyrimidine-5-Carboxamide (**8a**)

A suspension of (*S,E*)-*N*-(1-cyclopropyl-3-(methylsulfonyl)­allyl)-2-(1,1-difluoroethyl)-4-phenoxypyrimidine-5-carboxamide (**VVD-214**, 60 mg, 0.14 mmol) and Pd/C (20 mg, wet) in EtOAc (5 mL, 0.03 M) was stirred at room temperature for 1 h under an atmosphere of hydrogen gas (balloon, 15 psi). The mixture was filtered, and the filtrate was concentrated to give a residue. The residue was partitioned between water (10 mL) and EtOAc (15 mL × 3). The combined organic layers were washed with brine (10 mL × 2), dried over anhydrous Na_2_SO_4_, filtered, and concentrated under reduced pressure to give a residue. The residue was purified by prep-HPLC (Waters Xbridge BEH C18, 100 × 30 mm, 10 μm, 30–60% ACN in water (10 mM NH_4_HCO_3_)) to afford the title compound (24.1 mg, 40% yield) as a white solid. Purity = 100%, e.e. = 100%. MS (ESI^+^): *m*/*z* 440.1 [M + H]^+^. ^1^H NMR (400 MHz, chloroform-*d*): δ 9.45 (s, 1H), 7.64 (br d, *J* = 8.1 Hz, 1H), 7.54–7.45 (m, 2H), 7.41–7.33 (m, 1H), 7.23 (d, *J* = 7.8 Hz, 2H), 3.63 (dt, *J* = 2.9, 8.3 Hz, 1H), 3.31–3.10 (m, 2H), 2.93 (s, 3H), 2.41–2.17 (m, 2H), 1.85 (t, *J*_HF_ = 18.5 Hz, 3H), 1.03–0.95 (m, 1H), 0.72–0.64 (m, 1H), 0.62–0.53 (m, 1H), 0.46–0.37 (m, 2H).

### General Procedure XI: Suzuki Cross-Coupling of 2-Chloropyrimidine

A mixture of ethyl 2-(methylthio)-4-phenoxypyrimidine-5-carboxylate (1 equiv), sodium carbonate (1.2 equiv), the appropriate boronic ester or acid (1.2 equiv), and PdCl_2_(PPh_3_)_2_ (0.08 equiv) in 1.4-dioxane and water (4:1, 0.30 M) was heated at 80 °C for 16 h. After cooling to room temperature, the mixture was partitioned between water and EtOAc. The combined organic layers were dried over anhydrous Na_2_SO_4_, filtered, and concentrated under reduced pressure to give a residue. The residue was purified as described to afford the corresponding olefin.

**5:**
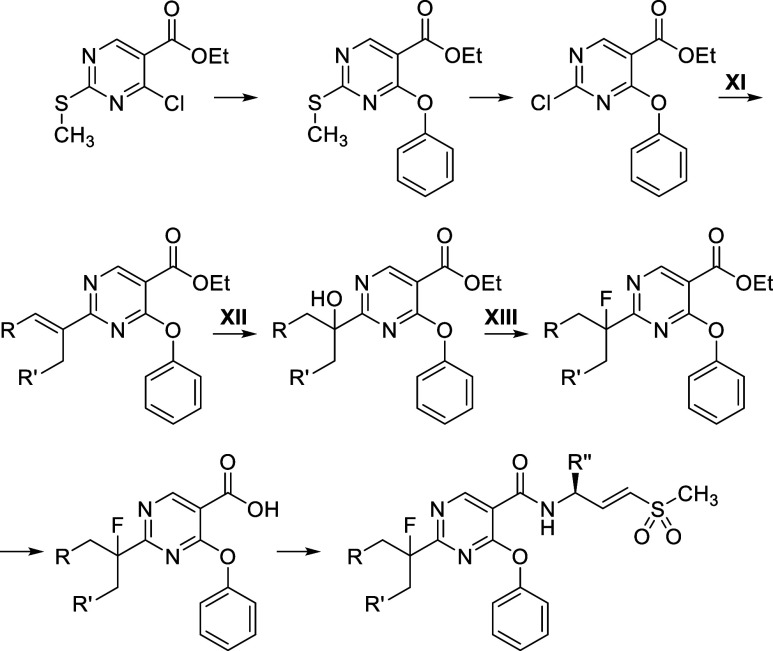
Synthesis of Pyrimidines **6a**, **6d**, and **7d**

### General Procedure XII: Mn­(III)-Catalyzed Mukaiyama Hydration

A mixture of the appropriate olefin (1 equiv), Mn­(dpm)_3_ (0.07 equiv), and phenylsilane (1 equiv) in DCM and IPA (3:1, 0.24 M) was stirred under an atmosphere of oxygen (balloon, 15 psi) at room temperature for 12 h. The mixture was filtered through a pad of Celite, and the filtrate was concentrated. The residue was purified as described to afford the corresponding tertiary alcohol.

### General Procedure XIII: Fluorination of Alcohol Using DAST

DAST (1.5 equiv) was added dropwise to a solution of the appropriate tertiary alcohol in DCM (0.12 M) at 0 °C. The mixture was allowed to warm to room temperature and stirred for 12 h. The mixture was partitioned between water and EtOAc. The combined organic layers were dried over anhydrous Na_2_SO_4_, filtered, and concentrated under reduced pressure to give a residue. The residue was purified as described to afford the corresponding alkyl fluoride.

#### (*S*,*E*)-2-(1-Fluorocyclopentyl)-*N*-(4-(methylsulfonyl)­but-3-en-2-yl)-4-phenoxypyrimidine-5-Carboxamide (**6a**)

Commercial ethyl 4-chloro-2-(methylthio)­pyrimidine-5-carboxylate (15 g, 64.7 mmol) and phenol (7.3 g, 77.7 mmol) were used for General Procedure X and purified by flash silica gel chromatography (SiO_2_ 230–400 mesh, eluent of 0–10% EtOAc/petroleum ether) to afford ethyl 2-(methylthio)-4-phenoxypyrimidine-5-carboxylate (18 g, 96% yield) as a white solid. ^1^H NMR (400 MHz, chloroform-*d*): δ 8.95 (s, 1H), 7.48–7.40 (m, 2H), 7.21–7.16 (m, 2H), 6.90–6.82 (m, 1H), 4.44 (q, *J* = 7.1 Hz, 2H), 2.26 (s, 3H), 1.43 (t, *J* = 7.1 Hz, 3H). SO_2_Cl_2_ (25 mL) in DCM (20 mL) was added to a solution of ethyl 2-(methylthio)-4-phenoxypyrimidine-5-carboxylate (7.0 g, 24.1 mmol) in ACN (100 mL, 0.24 M) at 0 °C. The mixture was allowed to warm to room temperature and stirred for 12 h. The mixture was poured into a saturated NaHCO_3_ aqueous solution (50 mL) at 0 °C. The aqueous layer was extracted with EtOAc (100 mL × 3). The combined organic layers were dried over anhydrous Na_2_SO_4_, filtered, and concentrated under reduced pressure to give a residue. The residue was purified by flash silica gel chromatography (SiO_2_ 230–400 mesh, eluent of 0–20% EtOAc/petroleum ether) to afford ethyl 2-chloro-4-phenoxypyrimidine-5-carboxylate (6.5 g, 97% yield) as a white solid. ^1^H NMR (400 MHz, chloroform-*d*): δ 8.97–9.02 (m, 1H), 7.42–7.49 (m, 2H), 7.28–7.34 (m, 1H), 7.15–7.22 (m, 2H), 4.45 (q, *J* = 7.13 Hz, 2H), 1.42 (t, *J* = 7.13 Hz, 3H). Cyclopent-1-en-1-ylboronic acid (96.4 mg, 0.86 mmol) was used for General Procedure XI and purified by flash silica gel chromatography (SiO_2_ 230–400 mesh, eluent of 0–20% EtOAc/petroleum ether) to afford ethyl 2-(cyclopent-1-en-1-yl)-4-phenoxypyrimidine-5-carboxylate (180 mg, 81% yield) as a white solid. MS (ESI^+^): *m*/*z* 311.2 [M + H]^+^. ^1^H NMR (400 MHz, chloroform-*d*): δ 9.12 (s, 1H), 7.40–7.49 (m, 2H), 7.28–7.32 (m, 1H), 7.23 (br d, *J* = 7.82 Hz, 2H), 6.87 (br s, 1H), 4.46 (q, *J* = 7.09 Hz, 2H), 2.67 (br t, *J* = 6.17 Hz, 2H), 2.54 (br s, 2H), 1.99 (quint, *J* = 7.46 Hz, 2H), 1.44 (t, *J* = 7.15 Hz, 3H). Ethyl 2-(cyclopent-1-en-1-yl)-4-phenoxypyrimidine-5-carboxylate (150 mg, 0.48 mmol) was used for General Procedure XII and purified by flash silica gel chromatography (SiO_2_ 230–400 mesh, eluent of 0–10% EtOAc/petroleum ether) to afford ethyl 2-(1-hydroxycyclopentyl)-4-phenoxypyrimidine-5-carboxylate (75 mg, 47% yield) as a white solid. ^1^H NMR (400 MHz, chloroform-*d*): δ 9.11 (s, 1H), 7.48–7.35 (m, 2H), 7.32–7.28 (m, 1H), 7.17 (d, *J* = 7.9 Hz, 2H), 4.46 (q, *J* = 7.1 Hz, 2H), 2.04–1.98 (m, 1H), 1.93–1.75 (m, 4H), 1.74–1.56 (m, 3H), 1.43 (t, *J* = 7.1 Hz, 3H). Ethyl 2-(1-hydroxycyclopentyl)-4-phenoxypyrimidine-5-carboxylate (200 mg, 0.61 mmol) was used for General Procedure XIII and purified by flash silica gel chromatography (SiO_2_ 230–400 mesh, eluent of 0–10% EtOAc/petroleum ether) to afford ethyl 2-(1-fluorocyclopentyl)-4-phenoxypyrimidine-5-carboxylate (80 mg, 40% yield) as a white solid. ^1^H NMR (400 MHz, chloroform-*d*): δ 9.14 (s, 1H), 7.46–7.38 (m, 2H), 7.27 (s, 1H), 7.17 (d, *J* = 7.7 Hz, 2H), 4.45 (q, *J* = 7.0 Hz, 2H), 2.19–2.03 (m, 3H), 2.03–1.93 (m, 1H), 1.92–1.77 (m, 2H), 1.74–1.59 (m, 2H), 1.43 (t, *J* = 7.1 Hz, 3H). Ethyl 2-(1-fluorocyclopentyl)-4-phenoxypyrimidine-5-carboxylate (110 mg, 0.33 mmol) was used for General Procedure VI to afford 2-(1-fluorocyclopentyl)-4-phenoxypyrimidine-5-carboxylic acid (75 mg, 75% yield) as a white solid. ^1^H NMR (400 MHz, chloroform-*d*): δ 9.33 (s, 1H), 7.49–7.42 (m, 2H), 7.36–7.28 (m, 1H), 7.22–7.15 (m, 2H), 2.21–1.94 (m, 4H), 1.93–1.82 (m, 2H), 1.75–1.61 (m, 2H). 2-(1-Fluorocyclopentyl)-4-phenoxypyrimidine-5-carboxylic acid (75 mg, 0.25 mmol) and (*S,E*)-4-(methylsulfonyl)­but-3-en-2-amine tosylate salt (79.7 mg, 0.25 mmol) were used for General Procedure VII and purified by prep-HPLC (Waters Xbridge BEH C18, 100 × 30 mm, 10 μm, 30–60% ACN in water (10 mM NH_4_HCO_3_)) to afford the title compound (61.5 mg, 57% yield) as a pale yellow solid. Purity = 100%, e.e. = 97.1%. MS (ESI^+^): *m*/*z* 434.2 [M + H]^+^. ^1^H NMR (400 MHz, chloroform-*d*): δ 9.41 (s, 1H), 7.58 (br d, *J* = 7.3 Hz, 1H), 7.52–7.45 (m, 2H), 7.41–7.32 (m, 1H), 7.19 (d, *J* = 8.2 Hz, 2H), 6.95 (dd, *J* = 5.0, 15.2 Hz, 1H), 6.55 (d, *J* = 15.2 Hz, 1H), 5.17–4.97 (m, 1H), 2.96 (s, 3H), 2.23–2.04 (m, 3H), 2.02–1.94 (m, 1H), 1.91–1.81 (m, 2H), 1.68 (br s, 2H), 1.47 (d, *J* = 7.0 Hz, 3H).

#### (*S*,*E*)-2-(2-Fluoropropan-2-yl)-*N*-(4-(methylsulfonyl)­but-3-en-2-yl)-4-phenoxypyrimidine-5-Carboxamide (**6d**)

4,4,5,5-Tetramethyl-2-(prop-1-en-2-yl)-1,3,2-dioxaborolane (332 mg, 1.97 mmol) was used for General Procedure XI and purified by flash silica gel chromatography (SiO_2_ 230–400 mesh, eluent of 0–20% EtOAc/petroleum ether) to afford ethyl 4-phenoxy-2-(prop-1-en-2-yl)­pyrimidine-5-carboxylate (180 mg, 35% yield) as a yellow oil. MS (ESI^+^): *m*/*z* 285.2 [M + H]^+^. Ethyl 4-phenoxy-2-(prop-1-en-2-yl)­pyrimidine-5-carboxylate (300 mg, 1.06 mmol) was used for General Procedure XII and purified by flash silica gel chromatography (SiO_2_ 230–400 mesh, eluent of 0–10% EtOAc/petroleum ether) to afford ethyl 2-(2-hydroxypropan-2-yl)-4-phenoxypyrimidine-5-carboxylate (200 mg, 63% yield) as a colorless oil. MS (ESI^+^): *m*/*z* 303.2 [M + H]^+^. Ethyl 2-(2-hydroxypropan-2-yl)-4-phenoxypyrimidine-5-carboxylate (100 mg, 0.33 mmol) was used for General Procedure XIII and purified by prep-TLC (SiO_2_ 400–500 mesh, EtOAc/petroleum ether = 1:5) to afford ethyl 2-(2-fluoropropan-2-yl)-4-phenoxypyrimidine-5-carboxylate (50 mg, 50% yield) as a colorless oil. ^1^H NMR (400 MHz, chloroform-*d*): δ 9.18 (s, 1H), 7.41–7.48 (m, 2H), 7.29–7.32 (m, 1H), 7.20 (d, *J* = 7.88 Hz, 2H), 4.48–4.48 (m, 1H), 4.48 (q, *J* = 7.13 Hz, 1H), 1.61 (d, *J*_HF_ = 21.6 Hz, 6H), 1.45 (t, *J* = 7.13 Hz, 3H). Ethyl 2-(2-fluoropropan-2-yl)-4-phenoxypyrimidine-5-carboxylate (50 mg, 0.16 mmol) was used for General Procedure VI to afford 2-(2-fluoropropan-2-yl)-4-phenoxypyrimidine-5-carboxylic acid (40 mg, 88% yield) as a colorless oil. MS (ESI^+^): *m*/*z* 277.1 [M + H]^+^. 2-(2-Fluoropropan-2-yl)-4-phenoxypyrimidine-5-carboxylic acid (40 mg, 0.14 mmol) and (*S,E*)-4-(methylsulfonyl)­but-3-en-2-amine tosylate salt (51.2 mg, 0.15 mmol) were used for General Procedure VII and purified by prep-HPLC (Waters Xbridge BEH C18, 100 × 30 mm, 10 μm, 30–60% ACN in water (10 mM NH_4_HCO_3_)) to afford the title compound (34.1 mg, 58% yield) as a white solid. Purity = 99.9%, e.e. = 96.6%. MS (ESI^+^): *m*/*z* 408.1 [M + H]^+^. ^1^H NMR (400 MHz, chloroform-*d*): δ 9.43 (s, 1H), 7.59 (br d, *J* = 7.50 Hz, 1H), 7.47–7.53 (m, 2H), 7.33–7.40 (m, 1H), 7.20 (d, *J* = 7.63 Hz, 2H), 6.96 (dd, *J* = 15.20, 4.94 Hz, 1H), 6.55 (dd, *J* = 15.13, 1.50 Hz, 1H), 5.01–5.10 (m, 1H), 2.96 (s, 3H), 1.594 (d, *J*_HF_ = 21.6 Hz, 3H), 1.587 (d, *J*_HF_ = 21.6 Hz, 3H), 1.48 (d, *J* = 7.13 Hz, 3H).

#### (*S*,*E*)-*N*-(1-Cyclopropyl-3-(methylsulfonyl)­allyl)-2-(2-Fluoropropan-2-yl)-4-phenoxypyrimidine-5-Carboxamide (**7d**)

2-(2-Fluoropropan-2-yl)-4-phenoxypyrimidine-5-carboxylic acid (50 mg, 0.18 mmol) and (*S,E*)-1-cyclopropyl-3-(methylsulfonyl)­prop-2-en-1-amine tosylate salt (synthesis described in Experimental Section, 66.3 mg, 0.19 mmol) were used for General Procedure VII and purified by prep-HPLC (Waters Xbridge BEH C18, 100 × 30 mm, 10 μm, 30–60% ACN in water (10 mM NH_4_HCO_3_)) to afford the title compound (41.8 mg, 53% yield) as a white solid. Purity = 100%, e.e. = 100%. MS (ESI^+^): *m*/*z* 434.1 [M + H]^+^. HR-MS (ESI^+^): *m*/*z* calcd for [C_21_H_24_FN_3_O_4_S + H]^+^: 434.1544; found, 434.1550. ^1^H NMR (400 MHz, chloroform-*d*): δ 9.42 (s, 1H), 7.77 (br d, *J* = 7.25 Hz, 1H), 7.47–7.54 (m, 2H), 7.34–7.40 (m, 1H), 7.22 (d, *J* = 8.00 Hz, 2H), 7.03 (dd, *J* = 15.07, 4.82 Hz, 1H), 6.61 (dd, *J* = 15.26, 1.25 Hz, 1H), 4.21–4.30 (m, 1H), 2.97 (s, 3H), 1.62–1.57 (m, 6H), 1.03–1.13 (m, 1H), 0.63–0.79 (m, 2H), 0.44–0.55 (m, 2H). ^1^H NMR (400 MHz, DMSO-*d*_6_): δ 8.94 (s, 1H), 8.84 (d, *J* = 8.3 Hz, 1H), 7.53–7.45 (m, 2H), 7.36–7.27 (m, 3H), 6.87 (d, *J* = 2.6 Hz, 2H), 4.29 (m, 1H), 2.98 (s, 3H), 1.562 (d, *J*_HF_ = 21.5 Hz, 3H), 1.558 (d, *J*_HF_ = 21.5 Hz, 3H), 1.12 (dtd, *J* = 10.7, 8.0, 4.1 Hz, 1H), 0.56 (td, *J* = 7.8, 2.0 Hz, 1H), 0.49 (qd, *J* = 7.5, 4.9 Hz, 2H), 0.41–0.36 (m, 1H). ^13^C NMR (101 MHz, DMSO-*d*_6_): δ 169.94 (d, *J*_CF_ = 22.1 Hz), 164.97, 162.05, 158.57, 151.67, 145.03, 130.06, 129.52, 125.73, 121.62, 116.52, 94.96 (d, *J*_CF_ = 173.4 Hz), 52.96, 42.21, 26.28 (d, *J*_CF_ = 24.7 Hz), 26.26 (d, *J*_CF_ = 24.5 Hz), 14.48, 3.06, 2.86. ^19^F NMR (470 MHz, DMSO-*d*_6_): δ −139.10. LC–MS report, SFC report, and NMR spectra for compound **7d** are included in Figure S5.

### Synthetic Procedure for Pyridine, Pyridazine, and Pyrazine Analogs

Pyridines **3a–c**, Pyridazine **3d**, and Pyrazine **3e**.

#### (*S*,*E*)-6-Cyclopentyl-*N*-(4-(methylsulfonyl)­but-3-en-2-yl)-2-phenoxynicotinamide (**3a**)

Ethyl 2,6-dichloronicotinate (0.5 g, 2.27 mmol) was used for General Procedure IV and purified by flash silica gel chromatography (ISCO; 12g SepaFlash Silica Flash Column, eluent of 0–3% EtOAc/petroleum ether gradient at 100 mL/min) to afford ethyl 2-chloro-6-(cyclopent-1-en-1-yl)­nicotinate (250 mg, 46% yield) as a yellow oil. MS (ESI^+^): *m*/*z* 252.0 [M + H]^+^. ^1^H NMR (400 MHz, chloroform-*d*): δ 8.11 (d, *J* = 8.0 Hz, 1H), 7.29 (d, *J* = 8.0 Hz, 1H), 6.82 (quint, *J* = 2.3 Hz, 1H), 4.41 (q, *J* = 7.1 Hz, 2H), 2.83–2.74 (m, 2H), 2.61 (ddd, *J* = 2.6, 5.0, 9.9 Hz, 2H), 2.07 (quint, *J* = 7.6 Hz, 2H), 1.42 (t, *J* = 7.1 Hz, 3H). Ethyl 2-chloro-6-(cyclopent-1-en-1-yl)­nicotinate (265 mg, 1.05 mmol) and phenol (148 mg, 1.58 mmol) were used for General Procedure X and purified by prep-TLC (SiO_2_ 400–500 mesh, EtOAc/petroleum ether = 1:5) to afford ethyl 6-(cyclopent-1-en-1-yl)-2-phenoxynicotinate (140 mg, 43% yield) as a colorless oil. MS (ESI^+^): *m*/*z* 310.1 [M + H]^+^. ^1^H NMR (400 MHz, chloroform-*d*): δ 8.24 (d, *J* = 7.9 Hz, 1H), 7.44–7.36 (m, 2H), 7.23–7.16 (m, 3H), 7.05 (d, *J* = 7.9 Hz, 1H), 6.50 (br s, 1H), 4.44–4.36 (m, 2H), 2.80 (br s, 1H), 2.66–2.57 (m, 2H), 2.53–2.47 (m, 1H), 1.99 (quint, *J* = 7.3 Hz, 2H), 1.46–1.37 (m, 3H). Ethyl 6-(cyclopent-1-en-1-yl)-2-phenoxynicotinate (140 mg, 0.45 mmol) was used for General Procedure V and purified by prep-TLC (SiO_2_ 400–500 mesh, EtOAc/petroleum ether = 1:5) to afford ethyl 6-cyclopentyl-2-phenoxynicotinate (65 mg, 46% yield) as a yellow oil. MS (ESI^+^): *m*/*z* 312.2 [M + H]^+^. ^1^H NMR (400 MHz, chloroform-*d*): δ 8.16 (d, *J* = 7.8 Hz, 1H), 7.39–7.32 (m, 2H), 7.19–7.11 (m, 3H), 6.92 (d, *J* = 7.9 Hz, 1H), 4.37 (q, *J* = 7.1 Hz, 2H), 3.10–3.00 (m, 1H), 1.95–1.83 (m, 2H), 1.65–1.54 (m, 6H), 1.36 (t, *J* = 7.1 Hz, 3H). Ethyl 6-cyclopentyl-2-phenoxynicotinate (65 mg, 0.21 mmol) was used for General Procedure VI to afford 6-cyclopentyl-2-phenoxynicotinic acid (60 mg, 100% yield) as a white solid. ^1^H NMR (400 MHz, chloroform-*d*): δ 8.42 (d, *J* = 7.8 Hz, 1H), 7.48–7.40 (m, 2H), 7.32–7.28 (m, 1H), 7.20 (d, *J* = 7.8 Hz, 2H), 7.04 (d, *J* = 7.9 Hz, 1H), 3.07 (quint, *J* = 7.1 Hz, 1H), 1.96–1.81 (m, 2H), 1.65–1.48 (m, 6H). 6-Cyclopentyl-2-phenoxynicotinic acid (60 mg, 0.21 mmol) and (*S,E*)-4-(methylsulfonyl)­but-3-en-2-amine tosylate salt (71.5 mg, 0.2 mmol) were used for General Procedure VII and purified by prep-HPLC (Phenomenex Gemini-NX C18, 75 × 30 mm, 3 μm, 30–70% ACN in water (10 mM NH_4_HCO_3_)) to afford the title compound (41.1 mg, 47% yield) as a pale yellow solid. Purity = 100%, e.e. = 92.2%. MS (ESI^+^): *m*/*z* 415.2 [M + H]^+^. ^1^H NMR (400 MHz, chloroform-*d*): δ 8.44 (d, *J* = 7.8 Hz, 1H), 7.93 (br d, *J* = 7.5 Hz, 1H), 7.48–7.40 (m, 2H), 7.29 (s, 1H), 7.17 (d, *J* = 7.9 Hz, 2H), 7.02 (d, *J* = 7.8 Hz, 1H), 6.96 (dd, *J* = 4.6, 15.1 Hz, 1H), 6.52 (dd, *J* = 1.5, 15.1 Hz, 1H), 5.09–4.98 (m, 1H), 3.10–3.01 (m, 1H), 2.92 (s, 3H), 1.89 (br d, *J* = 6.1 Hz, 2H), 1.61–1.52 (m, 6H), 1.44 (d, *J* = 7.1 Hz, 3H).

#### (*S*,*E*)-6-Cyclopentyl-*N*-(4-(methylsulfonyl)­but-3-en-2-yl)-4-phenoxynicotinamide (**3b**)

Methyl 4,6-dichloronicotinate (2.0 g, 9.71 mmol) was used for General Procedure IV and purified by flash silica gel chromatography (ISCO; 40g SepaFlash Silica Flash Column, eluent of 0–4% EtOAc/petroleum ether gradient at 100 mL/min) to afford methyl 4-chloro-6-(cyclopent-1-en-1-yl)­nicotinate (1.5 g, 65% yield) as a yellow oil. MS (ESI^+^): *m*/*z* 238.1 [M + H]^+^. ^1^H NMR (400 MHz, chloroform-*d*): δ 9.00 (s, 1H), 7.40 (s, 1H), 6.83–6.78 (m, 1H), 3.96 (s, 3H), 2.82–2.74 (m, 2H), 2.62 (qt, *J* = 2.6, 7.5 Hz, 2H), 2.14–2.03 (m, 2H). Methyl 4-chloro-6-(cyclopent-1-en-1-yl)­nicotinate (500 mg, 2.10 mmol) and phenol (198 mg, 2.10 mmol) were used for General Procedure X and purified by flash silica gel chromatography (ISCO; 12g SepaFlash Silica Flash Column, eluent of 0–25% EtOAc/petroleum ether gradient at 100 mL/min) to afford methyl 6-(cyclopent-1-en-1-yl)-4-phenoxynicotinate (310 mg, 50% yield) as a white solid. MS (ESI^+^): *m*/*z* 296.2 [M + H]^+^. ^1^H NMR (400 MHz, chloroform-*d*): δ 9.01 (s, 1H), 7.47–7.40 (m, 2H), 7.27–7.22 (m, 1H), 7.10 (d, *J* = 7.8 Hz, 2H), 6.69 (s, 1H), 6.62 (br s, 1H), 3.91 (s, 3H), 2.66–2.58 (m, 2H), 2.58–2.50 (m, 2H), 1.99 (quint, *J* = 7.5 Hz, 2H). Methyl 6-(cyclopent-1-en-1-yl)-4-phenoxynicotinate (310 mg, 1.05 mmol) was used for General Procedure V to afford crude methyl 6-cyclopentyl-4-phenoxynicotinate (310 mg, 99% yield) as a yellow oil, which was used in the subsequent reaction without further purification. MS (ESI^+^): *m*/*z* 298.1 [M + H]^+^. ^1^H NMR (400 MHz, chloroform-*d*): δ 8.98 (s, 1H), 7.47–7.40 (m, 2H), 7.27–7.23 (m, 1H), 7.12–7.08 (m, 2H), 6.54 (s, 1H), 3.90 (s, 3H), 3.04 (quint, *J* = 8.2 Hz, 1H), 2.03–1.93 (m, 2H), 1.82–1.73 (m, 2H), 1.72–1.61 (m, 4H). The crude methyl 6-cyclopentyl-4-phenoxynicotinate (100 mg, 0.34 mmol) was used for General Procedure VI to afford 6-cyclopentyl-4-phenoxynicotinic acid (95.3 mg, 100% yield) as a white solid. ^1^H NMR (400 MHz, chloroform-*d*): δ 9.20 (s, 1H), 7.53–7.45 (m, 2H), 7.37–7.31 (m, 1H), 7.19–7.13 (m, 2H), 6.54 (s, 1H), 3.16–3.04 (m, 1H), 2.06–1.95 (m, 2H), 1.82–1.73 (m, 2H), 1.71–1.59 (m, 4H). 6-Cyclopentyl-4-phenoxynicotinic acid (60 mg, 0.21 mmol) and (*S,E*)-4-(methylsulfonyl)­but-3-en-2-amine tosylate salt (68.1 mg, 0.21 mmol) were used for General Procedure VII and purified by prep-HPLC (Phenomenex Gemini-NX C18, 75 × 30 mm, 3 μm, 30–70% ACN in water (10 mM NH_4_HCO_3_)) to afford the title compound (65.4 mg, 75% yield) as a yellow solid. Purity = 100%, e.e. = 99.7%. MS (ESI^+^): *m*/*z* 415.1 [M + H]^+^. ^1^H NMR (400 MHz, chloroform-*d*): δ 9.24 (s, 1H), 7.57–7.44 (m, 3H), 7.41–7.35 (m, 1H), 7.16 (d, *J* = 8.0 Hz, 2H), 6.94 (dd, *J* = 4.7, 15.1 Hz, 1H), 6.55–6.45 (m, 2H), 5.11–4.99 (m, 1H), 3.13–3.02 (m, 1H), 2.92 (s, 3H), 1.98 (br s, 2H), 1.84–1.73 (m, 2H), 1.72–1.59 (m, 4H), 1.42 (d, *J* = 7.0 Hz, 3H).

#### (*S*,*E*)-5-Cyclopentyl-*N*-(4-(methylsulfonyl)­but-3-en-2-yl)-3-phenoxypicolinamide (**3c**)

Methyl 5-bromo-3-chloropicolinate (5 g, 20.0 mmol) and phenol (1.97 g, 21.0 mmol) were used for General Procedure X and purified by flash silica gel chromatography (SiO_2_ 230–400 mesh, eluent of 0–90% EtOAc/petroleum ether) to afford methyl 5-bromo-3-phenoxypicolinate (2.0 g, 33% yield). Methyl 5-bromo-3-phenoxypicolinate (500 mg, 1.62 mmol) was used for General Procedure IV and purified by flash silica gel chromatography (SiO_2_ 230–400 mesh, Eluent of 0–20% EtOAc/Petroleum ether) to afford methyl 5-(cyclopent-1-en-1-yl)-3-phenoxypicolinate (100 mg, 21% yield) as a white oil. MS (ESI^+^): *m*/*z* 296.1 [M + H]^+^. ^1^H NMR (400 MHz, chloroform-*d*): δ 8.57 (d, *J* = 1.5 Hz, 1H), 7.41–7.33 (m, 2H), 7.29 (d, *J* = 1.5 Hz, 1H), 7.18–7.12 (m, 1H), 7.01 (d, *J* = 7.9 Hz, 2H), 6.36 (br s, 1H), 3.92 (s, 3H), 2.67–2.62 (m, 2H), 2.56 (dt, *J* = 2.4, 7.4 Hz, 2H), 2.03 (quint, *J* = 7.5 Hz, 2H). Methyl 5-(cyclopent-1-en-1-yl)-3-phenoxypicolinate (100 mg, 0.34 mmol) was used for General Procedure V to afford crude methyl 5-cyclopentyl-3-phenoxypicolinate (80 mg, 80% yield) as a white solid, which was used in the subsequent reaction without further purification. ^1^H NMR (400 MHz, chloroform-*d*): δ 8.37 (d, *J* = 1.6 Hz, 1H), 7.40–7.34 (m, 2H), 7.19 (d, *J* = 1.8 Hz, 1H), 7.18–7.13 (m, 1H), 6.99 (d, *J* = 8.3 Hz, 2H), 3.93–3.90 (m, 3H), 3.07–2.97 (m, 1H), 2.13–2.04 (m, 2H), 1.82–1.75 (m, 2H), 1.72–1.66 (m, 2H), 1.54–1.48 (m, 2H). The crude methyl 5-cyclopentyl-3-phenoxypicolinate (80 mg, 0.27 mmol) was used for General Procedure VI to afford 5-cyclopentyl-3-phenoxypicolinic acid (50 mg, 66% yield) as a white solid. MS (ESI^+^): *m*/*z* 284.3 [M + H]^+^. ^1^H NMR (400 MHz, chloroform-*d*): δ 8.23 (s, 1H), 7.48–7.36 (m, 2H), 7.22 (br d, *J* = 7.1 Hz, 1H), 7.20–7.16 (m, 1H), 7.06 (br d, *J* = 8.0 Hz, 2H), 3.07–2.96 (m, 1H), 2.08 (br d, *J* = 5.1 Hz, 2H), 1.79 (br s, 2H), 1.70 (br s, 2H), 1.54–1.48 (m, 2H). 5-Cyclopentyl-3-phenoxypicolinic acid (50 mg, 0.18 mmol) and (*S,E*)-4-(methylsulfonyl)­but-3-en-2-amine tosylate salt (62.4 mg, 0.19 mmol) were used for General Procedure VII and purified by prep-HPLC (Phenomenex Gemini-NX C18, 150 × 25 mm, 10 μm, 37–67% ACN in water (10 mM NH_4_HCO_3_)) to afford the title compound (30.6 mg, 41% yield) as a yellow oil. Purity = 99.8%, e.e. = 96.9%. MS (ESI^+^): *m*/*z* 415.1 [M + H]^+^. ^1^H NMR (400 MHz, chloroform-*d*): δ 8.27 (d, *J* = 1.5 Hz, 1H), 7.88 (br d, *J* = 8.1 Hz, 1H), 7.40–7.33 (m, 2H), 7.22 (d, *J* = 1.5 Hz, 1H), 7.17–7.11, (m, 1H), 7.02–6.96 (m, 2H), 6.93 (dd, *J* = 4.4, 15.1 Hz, 1H), 6.48 (dd, *J* = 1.7, 15.1 Hz, 1H), 5.00–4.92 (m, 1H), 3.08–2.98 (m, 1H), 2.90 (s, 3H), 2.15–2.05 (m, 2H), 1.84–1.76 (m, 2H), 1.75–1.66 (m, 2H), 1.55 (td, *J* = 3.1, 5.9 Hz, 2H), 1.43 (d, *J* = 7.1 Hz, 3H).

#### (*S*,*E*)-5-Cyclopentyl-*N*-(4-(methylsulfonyl)­but-3-en-2-yl)-3-phenoxypyrazine-2-Carboxamide (**3d**)

Methyl 3,5-dichloropyrazine-2-carboxylate (500 mg, 2.42 mmol) was used for General Procedure IV and purified by flash silica gel chromatography (Biotage; 20g Agela Flash Silica Flash Column, eluent of 0–7% EtOAc/petroleum ether) to afford methyl 3-chloro-5-(cyclopent-1-en-1-yl)­pyrazine-2-carboxylate (300 mg, 52% yield) as a yellow oil. MS (ESI^+^): *m*/*z* 239.1 [M + H]^+^. Methyl 3-chloro-5-(cyclopent-1-en-1-yl)­pyrazine-2-carboxylate (200 mg, 0.84 mmol) and phenol (118 mg, 1.26 mmol) were used for General Procedure X and purified by prep-TLC (SiO_2_ 400–500 mesh, EtOAc/Petroleum ether = 1:3) to afford methyl 5-(cyclopent-1-en-1-yl)-3-phenoxypyrazine-2-carboxylate (180 mg, 72% yield) as a colorless oil. MS (ESI^+^): *m*/*z* 297.1 [M + H]^+^. Methyl 5-(cyclopent-1-en-1-yl)-3-phenoxypyrazine-2-carboxylate (150 mg, 0.51 mmol) was used for General Procedure V to afford crude methyl 5-cyclopentyl-3-phenoxypyrazine-2-carboxylate (140 mg, 93% yield) as a colorless oil. MS (ESI^+^): *m*/*z* 299.1 [M + H]^+^. The crude methyl 5-cyclopentyl-3-phenoxypyrazine-2-carboxylate (80 mg, 0.27 mmol) was used for General Procedure VI to afford 5-cyclopentyl-3-phenoxypyrazine-2-carboxylic acid (65 mg, 85% yield) as a colorless oil. 5-Cyclopentyl-3-phenoxypyrazine-2-carboxylic acid (65 mg, 0.23 mmol) and (*S,E*)-4-(methylsulfonyl)­but-3-en-2-amine tosylate salt (80.0 mg, 0.25 mmol) were used for General Procedure VII and purified by prep-HPLC (Waters Xbridge BEH C18, 100 × 30 mm, 10 μm, 45–80% ACN in water (10 mM NH_4_HCO_3_)) to afford the title compound (24.7 mg, 26% yield) as a yellow oil. Purity = 100%. MS (ESI^+^): *m*/*z* 416.1 [M + H]^+^. ^1^H NMR (400 MHz, chloroform-*d*): δ 8.17–8.11 (m, 1H), 7.80 (br d, *J* = 7.9 Hz, 1H), 7.44–7.36 (m, 2H), 7.26–7.20 (m, 1H), 7.16 (d, *J* = 7.6 Hz, 2H), 6.97 (dd, *J* = 4.6, 15.1 Hz, 1H), 6.56 (dd, *J* = 1.3, 15.1 Hz, 1H), 5.10–4.97 (m, 1H), 3.19–3.08 (m, 1H), 2.95 (s, 3H), 2.00–1.88 (m, 2H), 1.64–1.60 (m, 4H), 1.59–1.53 (m, 2H), 1.48 (d, *J* = 7.1 Hz, 3H), 1.45–1.44 (m, 1H).

**Scheme 6:**
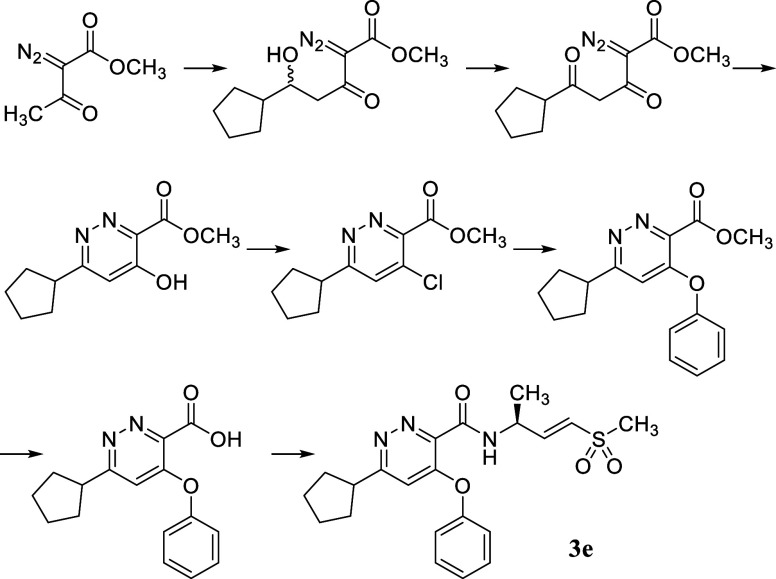
Synthesis of Pyridazine **3e**

#### (*S*,*E*)-6-Cyclopentyl-*N*-(4-(methylsulfonyl)­but-3-en-2-yl)-4-phenoxypyridazine-3-Carboxamide (**3e**)

TiCl_4_ (1.28 mL, 11.6 mmol, 1.1 equiv) was added to a solution of methyl 2-diazo-3-oxobutanoate (1.5 g, 10.6 mmol, 1 equiv) and triethylamine (1.62 mL, 11.6 mmol, 1.1 equiv) in DCM (20 mL, 0.53 M) at 0 °C. After 1 h, cyclopentanecarbaldehyde (932 mg, 9.50 mmol, 0.9 equiv) was added to the mixture and allowed to warm to room temperature. After 4 h, the mixture was quenched with saturated aqueous NH_4_Cl and extracted with DCM (20 mL × 3). The combined organic layers were washed with saturated aqueous NaHCO_3_ (15 mL × 3), dried over anhydrous Na_2_SO_4_, filtered, and concentrated under reduced pressure to give a residue. The residue was purified by flash silica gel chromatography (SiO_2_ 230–400 mesh, eluent of 10–30% EtOAc/petroleum ether) to afford methyl 5-cyclopentyl-2-diazo-5-hydroxy-3-oxopentanoate (1.3 g, 51% yield) as a yellow oil. ^1^H NMR (400 MHz, chloroform-*d*): δ 3.86–3.92 (m, 1H), 3.85 (s, 3H), 3.14 (dd, *J* = 17.01, 2.25 Hz, 1H), 2.94 (dd, *J* = 16.95, 9.57 Hz, 2H), 1.89–2.02 (m, 1H), 1.79–1.89 (m, 1H), 1.67–1.77 (m, 1H), 1.55–1.67 (m, 4H), 1.39–1.49 (m, 1H), 1.19–1.30 (m, 1H). IBX (1.97 g, 7.0 mmol, 1.3 equiv) was added to a solution of methyl 5-cyclopentyl-2-diazo-5-hydroxy-3-oxopentanoate (1.3 g, 5.41 mmol, 1.0 equiv) in ACN (15 mL, 0.36 M) at room temperature. After 2 h, the mixture was filtered and concentrated under reduced pressure to afford crude methyl 5-cyclopentyl-2-diazo-3,5-dioxopentanoate (1.3 g, 100% yield) as a yellow oil. ^1^H NMR (400 MHz, chloroform-*d*): δ 4.05 (s, 2H), 3.81 (s, 3H), 2.59–2.72 (m, 1H), 1.79–1.96 (m, 8H). Hexamethylphosphorous triamide (822 mg, 5.04 mmol, 1 equiv) was added to a solution of the crude methyl 5-cyclopentyl-2-diazo-3,5-dioxopentanoate (1.2 g, 5.04 mmol, 1 equiv) in DCM (15 mL, 0.34 M) at room temperature. After 16 h, the mixture was concentrated and purified by prep-HPLC to afford methyl 6-cyclopentyl-4-hydroxypyridazine-3-carboxylate (0.8 g, 71% yield) as a yellow solid. MS (ESI^+^): *m*/*z* 223.1 [M + H]^+^. ^1^H NMR (400 MHz, methanol-*d*_4_): δ 6.59 (s, 1H), 3.90 (s, 3H), 3.05 (quint, *J* = 8.38 Hz, 1H), 2.10–2.20 (m, 2H), 1.82–1.93 (m, 2H), 1.63–1.82 (m, 4H). Methyl 6-cyclopentyl-4-hydroxypyridazine-3-carboxylate (200 mg, 0.90 mmol) was used for General Procedure IX to afford crude methyl 4-chloro-6-cyclopentylpyridazine-3-carboxylate (216 mg, 100% yield) as a yellow oil. ^1^H NMR (400 MHz, chloroform-*d*): δ 7.98 (s, 1H), 4.10 (s, 3H), 3.76–3.85 (m, 1H), 2.35–2.45 (m, 2H), 1.93–2.00 (m, 2H), 1.75–1.91 (m, 4H). The crude methyl 4-chloro-6-cyclopentylpyridazine-3-carboxylate (200 mg, 0.83 mmol) and phenol (78.2 mg) were used for General Procedure X while cesium carbonate (1.6 g, 6 equiv) was used as the base, and purified by prep-TLC (SiO_2_ 400–500 mesh, EtOAc/Petroleum ether = 1:1) to afford methyl 6-cyclopentyl-4-phenoxypyridazine-3-carboxylate (70 mg, 28% yield) as a white solid. ^1^H NMR (400 MHz, chloroform-*d*): δ 7.43–7.53 (m, 2H), 7.30–7.37 (m, 1H), 7.12 (d, *J* = 7.88 Hz, 2H), 6.63 (s, 1H), 4.04 (s, 3H), 3.27 (quint, *J* = 8.10 Hz, 1H), 2.00–2.15 (m, 2H), 1.78–1.84 (m, 2H), 1.64–1.77 (m, 4H). Methyl 6-cyclopentyl-4-phenoxypyridazine-3-carboxylate (70 mg, 0.23 mmol) was used for General Procedure VI to afford 6-cyclopentyl-4-phenoxypyridazine-3-carboxylic acid (67 mg, 100% yield) as a yellow oil. ^1^H NMR (400 MHz, methanol-*d*_4_): δ 7.45–7.55 (m, 2H), 7.29–7.37 (m, 1H), 7.22 (d, *J* = 7.75 Hz, 2H), 6.66 (s, 1H), 3.22 (quint, *J* = 8.25 Hz, 1H), 1.98–2.08 (m, 2H), 1.73–1.79 (m, 2H), 1.60–1.73 (m, 4H). 6-Cyclopentyl-4-phenoxypyridazine-3-carboxylic acid (67 mg, 0.24 mmol) and (*S,E*)-4-(methylsulfonyl)­but-3-en-2-amine tosylate salt (83.3 mg, 0.26 mmol) were used for General Procedure VII and purified by prep-HPLC (Waters Xbridge BEH C18, 150 × 40 mm, 10 μm, 30–60% ACN in water (10 mM NH_4_HCO_3_)) to afford the title compound (85.9 mg, 87% yield) as a white solid. Purity = 99.3%, e.e. = 96.2%. MS (ESI^+^): *m*/*z* 416.1 [M + H]^+^. ^1^H NMR (400 MHz, chloroform-*d*): δ 8.10 (br d, *J* = 8.13 Hz, 1H), 7.48 (t, *J* = 7.82 Hz, 2H), 7.30–7.37 (m, 1H), 7.14 (br d, *J* = 7.75 Hz, 2H), 6.98 (dd, *J* = 15.13, 4.75 Hz, 1H), 6.67 (s, 1H), 6.59 (d, *J* = 15.13 Hz, 1H), 4.95–5.12 (m, 1H), 3.14–3.32 (m, 1H), 2.95 (s, 3H), 2.01–2.15 (m, 2H), 1.65–1.89 (m, 6H), 1.48 (d, *J* = 7.00 Hz, 3H).

### Helicase Activity Assay

The helicase assay was adapted from a published protocol.^39^ Human WRN construct Helicase Core w/HRDC (hWRN^519–1227^) was diluted in assay buffer (50 mM Tris-HCl pH 7.5, 2 mM MgCl_2_, 100 mM NaCl, 0.01% Tween-20, 0.0025 U ml^–1^ poly deoxyinosinic-deoxycytidylic acid sodium salt, 0.003% bovine serum albumin, 1 mM DTT), with or without 0.2 mM ATP, and plated in the assay plate (10 μL per well). Proteins were preincubated with compounds (12-point dose–response, 0–50 μM, 3-fold serial dilutions, 0.5% DMSO) at room temperature for 30 min. The reaction was initiated by the addition of a 10 μL per well mixture containing ATP, the DNA-capturing strand (GAA­CGA­ACA­CAT­CGGG­TACG), and the TAMRA-labeled DNA duplex DNA OLIGOA-BHQ2: TTT­TTT­TTT­TTT­TTT­TTT­TTT­TTT­TTT­TTT­CGT­ACC­CGA­TGT­GTTC­GTTC/BHQ2, OLIGOB-TAMRA: TAMRA/GAA­CGA­ACA­CAT­CGG­GTA­CGT­TTT­TTT­TTT­TTT­TTT­TTT­TTT­TTTT­TTTT. After 30 min, end point measurement of fluorescence was performed (ex./em. 535/585 nm, CLARIOstar (BMG Labtech)). Activity was normalized to no-enzyme negative controls (0%) and to DMSO-treated positive control (100%). The reference compound NSC 617145 (Tocris, no. 5340), a nonspecific helicase inhibitor, was used as a control for inhibition. Unless otherwise specified, all results were derived from a representative experiment with two technical replicates.

### Cellular Growth Inhibition Assay

Cell lines HCT116 (ATCC, no. CCL-228) and SW480 (ATCC, no. CCL-228) were authenticated at ATCC using the short tandem repeat method, cultured according to the manufacturer’s instructions, and routinely tested for mycoplasma. HCT116 and SW480 were seeded overnight in 384-well plates at 500–1000 cells per well in complete media. Compound dilutions were then added to the remaining cells in a 1:3, 10-point dose–response format starting at 100 μM. At the end of the 5 day incubation period, CTG 2.0 reagent was added to each well. Luminescence was read on a CLARIOstar plate reader (BMG Labtech). Relative growth was calculated by normalizing the growth of compound-treated cells to that of DMSO-treated cells using the following equation: (treatment *t*_5_ *– t*_0_)/(DMSO *t*_5_ *– t*_0_)) × 100, where *t*_5_ represents cell viability after 5 days of treatment and *t*_0_ represents cell viability before treatment. The concentration required to reach half-maximal response (GI_50_) is reported. Unless otherwise specified, all figures were derived from a representative experiment with two technical replicates.

### GSH Reactivity Assay

Compounds (500 μM) were incubated at room temperature for 2 and 6 h in buffer comprising 0.1 M Tris, pH 8.8 and 30% acetonitrile with 50 μM GSH. At the end of the designated time point, samples were spiked with 1 mM final concentration of Ellman’s reagent, and absorbance was read at 440 nm. The concentration of GSH remaining was derived from a standard curve, and the observed rate (*k*_obs_/[*I*]) was calculated assuming pseudo-first-order reaction kinetics from the following equations: d­(GSH)/d*t* = −*k* × (GSH), (GSH)_*t*_ = (GSH)_*t*0_ × e^–*kt*^.

### In Vivo Efficacy Study

Animals were maintained in accordance with the guidelines for the care and use of laboratory animals as approved (ACUP number: EB17-010-032) by the appropriate Institutional Animal Care and Use Committees from the National Institutes of Health. All animal procedures were conducted in a facility at Vividion (San Diego, CA), accredited by the Association for Assessment and Accreditation of Laboratory Animal Care.

Five-week-old female homozygous Foxn1 < nu > mice were obtained from Jackson Laboratories (catalogue no. 007850). Mice were maintained under pathogen-free conditions on a 12/12 h light/dark cycle with room temperature at 24 ± 3 °C and ambient relative humidity. Sterilized food and water were provided ad libitum.

Following the acclimation period (3–7 days), each mouse was inoculated subcutaneously in the right lower flank with 10^7^ HCT116 tumor cells of 95% or greater viability in a 1:1 mix of phosphate buffered saline (PBS) and Matrigel (1:1 v/v; Corning, catalogue no. 356237). Mice were randomized into treatment groups (*n* = 5–10 per group) based on tumor size, with a mean of roughly 150 mm^3^ for randomization across all studies. Studies were not blinded. The start of the study, where randomization and dosing began, was designated as Day 0.

Mice were administered study compound (via SC for **5d**, PO for **VVD-214**) or vehicle daily, and tumors and body weights were measured twice per week. Tumor size was measured in two dimensions using a digital caliper, with volume calculated in cubic millimeters using the formula *V* (mm^3^) = (*a* × *b* ^2^)/2, where a and b are the length and width of the tumor, respectively. Tumors were snap-frozen in liquid nitrogen and stored in bead-beater tubes at −80 °C for proteomic analysis.

### Intact Protein Mass Spectrometry and Rate Determination

To observe covalent ligand engagement of recombinant WRN protein, at 2 μM, was incubated with increasing concentrations of compound in 25 mM HEPES, pH 7.5, 150 mM NaCl, 1 mM MgCl_2_, 1 mM DTT, 10 mM octyl β-d-glucopyranoside, and 200 μM ADP. The reaction was quenched with 0.5% FA (final concentration) and analyzed on an Agilent LC1290 Infinity II instrument coupled to a 6545 QTOF MS (Agilent Technologies). A sample volume of 15 μL, equivalent to approximately 1.5 pmol of WRN protein, was injected. The resulting data were deconvoluted to protein masses using Agilent MassHunter BioConfirm Software, v.11.0. Peak intensities were used to quantify the percentage of compound modification relative to the unmodified protein peak by dividing modified protein intensity by the sum of the unmodified and modified protein intensities. *k*_obs_/[*I*] was calculated using assumptions for pseudo-first-order reaction kinetics (d­(compound)/d*t* = −*k* × (compound), (compound)_*t*_ = (compound)_*t0*_ × e^–*kt*^) and averaged for each inhibitor concentration; *k*_obs_ was determined by dividing these values by [I] for each concentration.

### In Situ, In Vitro, and In Vivo Sample Preparation for Parallel Reaction Monitoring and DIA Experiments

For in situ experiments, OCI-AML2 cells (5 million cells per well) grown with RPMI supplemented with 10% fetal bovine serum were treated with DMSO or compound for 2 h. Following compound wash out, cells were lysed by sonication in PBS, and incubated with 200 μM IA-DTB for 1 h at room temperature. For in vitro experiments, cells were harvested and frozen until use. Cell pellets were lysed by probe sonication in PBS, and protein concentration was normalized to 2.5 mg/mL. DMSO or compound was added to 500 μg of protein lysate and incubated at room temperature for 1 h followed by incubation with 200 μM IA-DTB for 1 h at room temperature. For in vivo experiments, tumor tissues were homogenized in Pierce IP lysis buffer via bead beating and sonication. Protein concentration was normalized, and 300 μg of protein lysate was incubated with 200 μM IA-DTB for 1 h at room temperature. Following probe treatment for either in situ, in vitro, or in vivo experiments, the protein was acetone-precipitated and then resuspended in 9 M urea with 50 mM ammonium bicarbonate. Proteins were reduced and alkylated by the addition of DTT and iodoacetamide (10 and 30 mM, respectively) and then digested with trypsin. IA-DTB labeled peptides were isolated with streptavidin agarose resin. Enriched cysteine-containing peptides were eluted by the addition of 50% acetonitrile (ACN) with 0.1% FA and dried in a SpeedVac vacuum concentrator until analysis.

### PRM Target Engagement Assay

Dried peptides were resuspended in 3% ACN with 0.1% FA, and 10% of the sample was analyzed by LC-PRM MS/MS. Probe-labeled peptides were concentrated onto an Acclaim PepMap100C18 loading column (Thermo, 100 μm × 2 cm, 5 μm particle size) and separated on a C18 analytical column (Thermo, 75 μm × 15 cm, 2 μm particle size) using a Dionex Ultimate 3000 nano-LC. Peptides were separated using a 12.7 min gradient going from 6 to 32.5% B solvent (96.4% ACN, 3.5% DMSO, 0.1% FA) mixed with A solvent (96.4% water, 3.5% DMSO, 0.1% FA). Peptides were analyzed by PRM on a Thermo Exploris 120. Precursor ions were fragmented and measured using a normalized collision energy of 25%, 0.7 *m*/*z* Q1 resolution, 30,000 Orbitrap resolution, 70% RF lens, in positive polarity mode.

Target engagement (TE) of WRN_C727 was measured by monitoring peptide NPQITCTGFDRPNLYLEVR (854.1162 *m*/*z*). Additional peptides were measured to quantify total WRN protein levels, in addition to other peptides not impacted by WRN inhibition. Retention time standard peptides were also included in the method and were used for global normalization for each sample.

MS data were analyzed using Skyline v.21.2.0.369 (MacCoss Lab, University of Washington). Peptide quantification was performed by summing the peak areas corresponding to three to six fragment ions. Fragment ions were preselected from an in-house-generated reference spectral library. Percent TE for each peptide was determined by comparing the average peptide area under the curve (AUC) obtained from each dosing group to the average peptide AUC of the DMSO-treated group. TE was measured as a loss of cysteine 727-containing peptide signal. Based on the average percent inhibition for each compound concentration, a TE curve was generated, and the TE_50_ was estimated based on the concentration found in the middle of the curves.

### DIA Selectivity Assay

Dried peptides were resuspended in 5% ACN with 0.1% FA, and 16% of the sample was analyzed by reversed phase LC–MS/MS using data independent (DIA)-PASEF acquisition mode on a timsTOF HT (Bruker Daltonics). Peptides were loaded onto a PepMap Neo C18 trap cartridge (Thermo, 300 μm × 5 mm, 5 μm particle size) and separated on a double nanoviper PepMap Neo C18 column (Thermo, 75 μm × 150 mm, 2 μm particle size) using a Thermo Vanquish Neo UHPLC system at 1.2 μL/min with the following gradient: 3 to 8.5% B solvent (ACN with 0.1% FA) in 1.5 min, 8.5 to 11% B in 1.5 min, 11 to 30% B in 55 min, and 30 to 42% B in 3 min. The capillary voltage was set to 1700 V, and the dry gas was at 3.0 L/min. MS1 scans were acquired from 100 to 1700 *m*/*z,* and 25 variable *m*/*z* window PASEF scans were subsequently acquired for MS2 with a mass range of 351.7–1399.7 *m*/*z* and an ion mobility range of 0.70–1.40 1/K0.

Data were searched against a human DDA spectral library using Spectronaut 19 (v. 19.0.240606). MS2 XIC extraction was performed using Spectronaut’s default dynamic mass tolerance setting. Peptides were quantified across runs when they were identified in more than 5% of runs. Peptides were filtered using a max control CV of 50% and group standard deviation of 40%, minimum peak area of 300, and *q*-value cutoff of 0.05. All data points that did not exhibit a dose response compared to a 5× higher concentration treatment were removed from the data set.

## Supplementary Material





## Notes

Additional analytical data for compound **2a**, **5d**, **6c**, **7b**, **7d**, and **VVD-214**; A_NMR_ calculations; additional SAR of pyrimidine ether; and TE time-course for rate determination (PDF)
Molecular formula strings (CSV)

## Abbreviations

ADP: adenosine diphosphate
ATP: adenosine triphosphate
DIA: data-independent acquisition
DIAD: diisopropyl azodicarboxylate
dpm: dipivaloylmethanate
dppf: 1,1′-bis­(diphenylphosphino)­ferrocene
e.e.: enantiomeric excess
GSH: glutathione
IMHB: intramolecular hydrogen bond
IV: intravenous
LipE: lipophilic efficiency
MMR: mismatch repair
MMS: microsatellite stable
MSI-H: microsatellite instability high
PO: oral
PPB: plasma protein binding
PRM: parallel reaction monitoring
SC: subcutaneous
TE: target engagement
TGI: tumor growth inhibition
WRN: Werner syndrome helicase.
