# Supplementary material for: Identification of VVD-214/RO7589831, a Clinical-Stage, Covalent Allosteric Inhibitor of WRN Helicase for the Treatment of MSI-High Cancers
Source: J Med Chem. 2025 Sep 18;68(24):25912–38. doi: 10.1021/acs.jmedchem.5c01805 (PMC12751020; doi:10.1021/acs.jmedchem.5c01805)
Supplement: Supplementary file 1 [file jm5c01805_si_001.pdf]

Supporting Information

Identification of VVD-214/RO7589831, a Clinical-Stage, Covalent Allosteric Inhibitor of WRN Helicase for the Treatment of MSI-High Cancers

Shota Kikuchi*, Jason C. Green, Don C. Rogness, Betty Lam, Zachary A. Owyang, Robert D. Malmstrom, Ali Tabatabaei, Aaron N. Snead, Melissa A. Hoffman, Steffen M. Bernard, Paige Ashby, Kelsey N. Lamb, Benjamin D. Horning, Kristen A. Baltgalvis, Kent T. Symons, Thomas A. Glaza, Chu-Chiao Wu, Xiaodan Song, Martha K. Pastuszka, John J. Sigler, Jonathan Pollock, Laurence Burgess, Gabriel M. Simon, Matthew P. Patricelli, and David S. Weinstein*

Vividion Therapeutics Inc., San Diego, California, United States

*Corresponding Authors – Shota Kikuchi, E-mail: shotak@vividion.com; David S. Weinstein, E-mail: davidw@vividion.com.

Table of Contents

1. Additional Analytical Data for Compounds Tested in *In Vivo* Experiments S2-S12
2. A_{NMR} Calculations S13-S19
3. Additional SAR of Pyrimidine Ether S20
4. TE Time-course for Rate Determination S21-S22

Additional Analytical Data for Compounds Tested in *In Vivo* Experiments

(a)

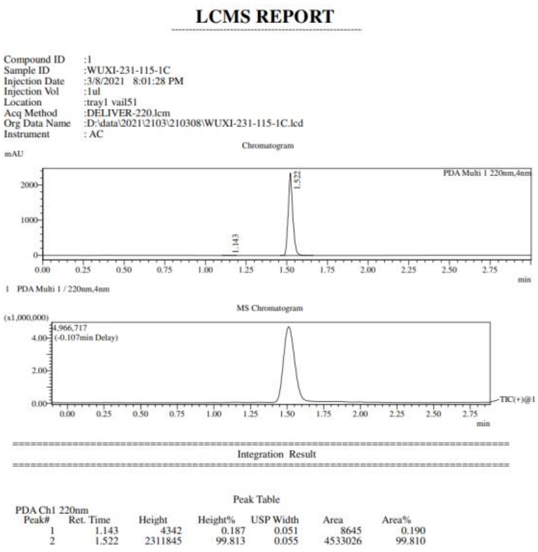

LCMS REPORT

Compound ID	:1
Sample ID	:WUXI-231-115-1C
Injection Date	:3/8/2021 8:01:28 PM
Injection Vol	:1ul
Location	:tray1 vail51
Acq Method	:DELIVER-220.lcm
Org Data Name	:D:\data\2021\2103\210308\WUXI-231-115-1C.lcd
Instrument	: AC

Integration Result

Peak Table

PDA Ch1 220nm

Peak#	Ret. Time	Height	Height%	USP Width	Area	Area%
1	1.143	4342	0.187	0.051	8645	0.190
2	1.522	2311845	99.813	0.055	4533026	99.810

(b)

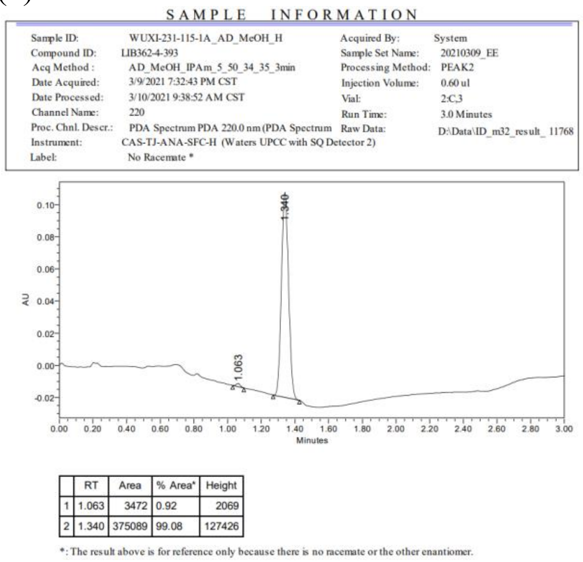

SAMPLE INFORMATION

Sample ID:	WUXI-231-115-1A_AD_MeOH_H	Acquired By:	System
Compound ID:	LIB362-4-393	Sample Set Name:	20210309_EE
Acq Method :	AD_MeOH_IPAm_5_50_34_35_3min	Processing Method:	PEAK2
Date Acquired:	3/9/2021 7:32:43 PM CST	Injection Volume:	0.60 ul
Date Processed:	3/10/2021 9:38:52 AM CST	Vial:	2:C,3
Channel Name:	220	Run Time:	3.0 Minutes
Proc. Chnl. Descr.:	PDA Spectrum PDA 220.0 nm (PDA Spectrum	Raw Data:	D:\Data\ID_m32_result_ 11768
Instrument:	CAS-TJ-ANA-SFC-H (Waters UPCC with SQ Detector 2)		
Label:	No Racemate *		

	RT	Area	% Area*	Height
1	1.063	3472	0.92	2069
2	1.340	375089	99.08	127426

*: The result above is for reference only because there is no racemate or the other enantiomer.

(c)

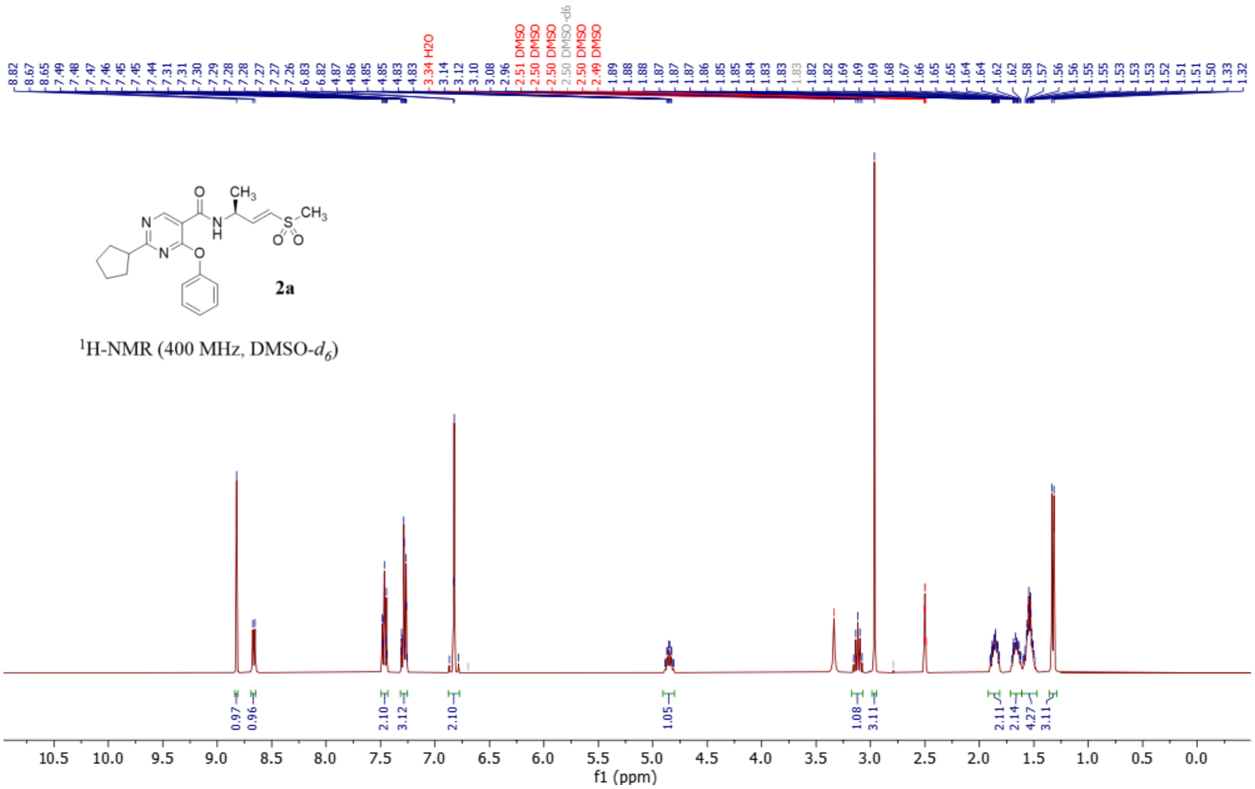

(d)

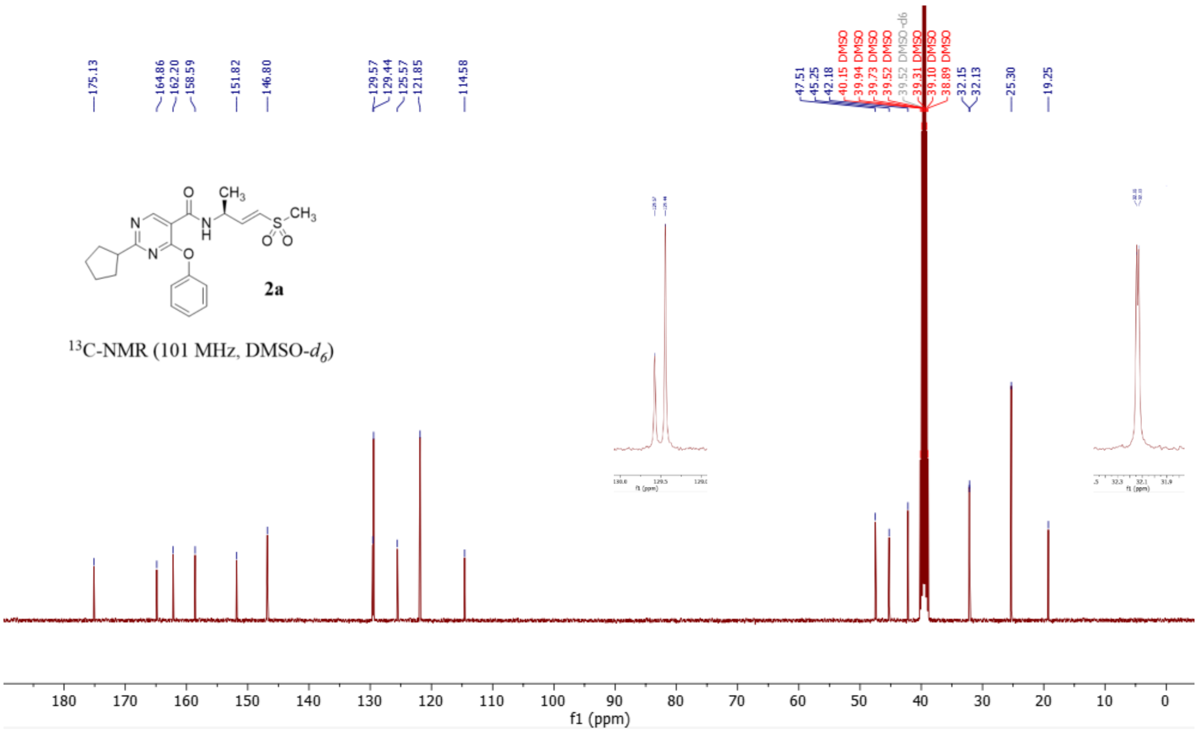

Figure S1. Additional analytical data for compound **2a**. (a) LC-MS report, (b) SFC report, (c) ^{1}H-NMR, (d) ^{13}C-NMR.

(a)

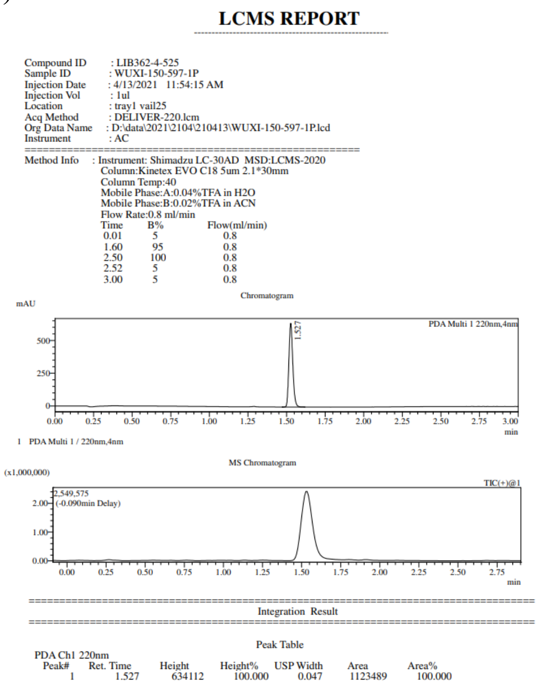

LCMS REPORT

Compound ID : LIB362-4-525
Sample ID : WUXI-150-597-1P
Injection Date : 4/13/2021 11:54:15 AM
Injection Vol : 1ul
Location : tray1 vail25
Acq Method : DELIVER-220.lcm
Org Data Name : D:\data\2021\2104\210413\WUXI-150-597-1P.lcd
Instrument : AC

Method Info : Instrument: Shimadzu LC-30AD MSD:LCMS-2020
Column:Kinetex EVO C18 5um 2.1*30mm
Column Temp:40
Mobile Phase:A:0.04%TFA in H2O
Mobile Phase:B:0.02%TFA in ACN
Flow Rate:0.8 ml/min

Time	B%	Flow(ml/min)
0.01	5	0.8
1.60	95	0.8
2.50	100	0.8
2.52	5	0.8
3.00	5	0.8

Integration Result

Peak Table

PDA Ch1 220nm

Peak#	Ret. Time	Height	Height%	USP Width	Area	Area%
1	1.527	634112	100.000	0.047	1123489	100.000

(b)

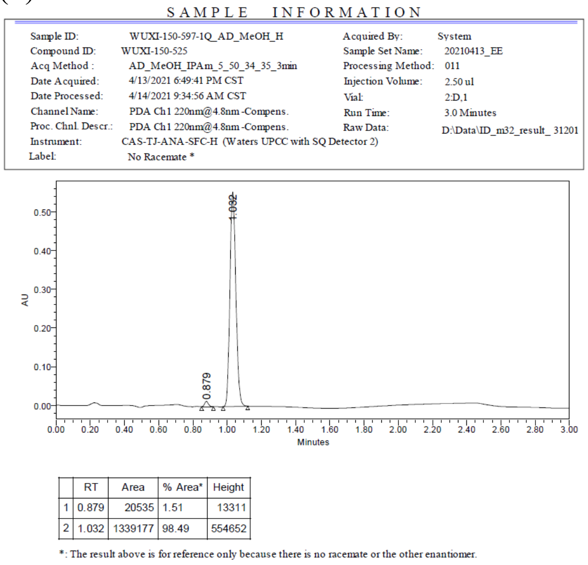

S A M P L E   I N F O R M A T I O N

Sample ID:	WUXI-150-597-1Q_AD_MeOH_H	Acquired By:	System
Compound ID:	WUXI-150-525	Sample Set Name:	20210413_EE
Acq Method :	AD_MeOH_IPAm_5_50_34_35_3min	Processing Method:	011
Date Acquired:	4/13/2021 6:49:41 PM CST	Injection Volume:	2.50 ul
Date Processed:	4/14/2021 9:34:56 AM CST	Vial:	2:D,1
Channel Name:	PDA Ch1 220nm@4.8nm -Compens.	Run Time:	3.0 Minutes
Proc. Chnl. Descr.:	PDA Ch1 220nm@4.8nm -Compens.	Raw Data:	D:\Data\ID_m32_result_ 31201
Instrument:	CAS-TJ-ANA-SFC-H (Waters UPCC with SQ Detector 2)		
Label:	No Racemate *		

	RT	Area	% Area*	Height
1	0.879	20535	1.51	13311
2	1.032	1339177	98.49	554652

*: The result above is for reference only because there is no racemate or the other enantiomer.

(c)

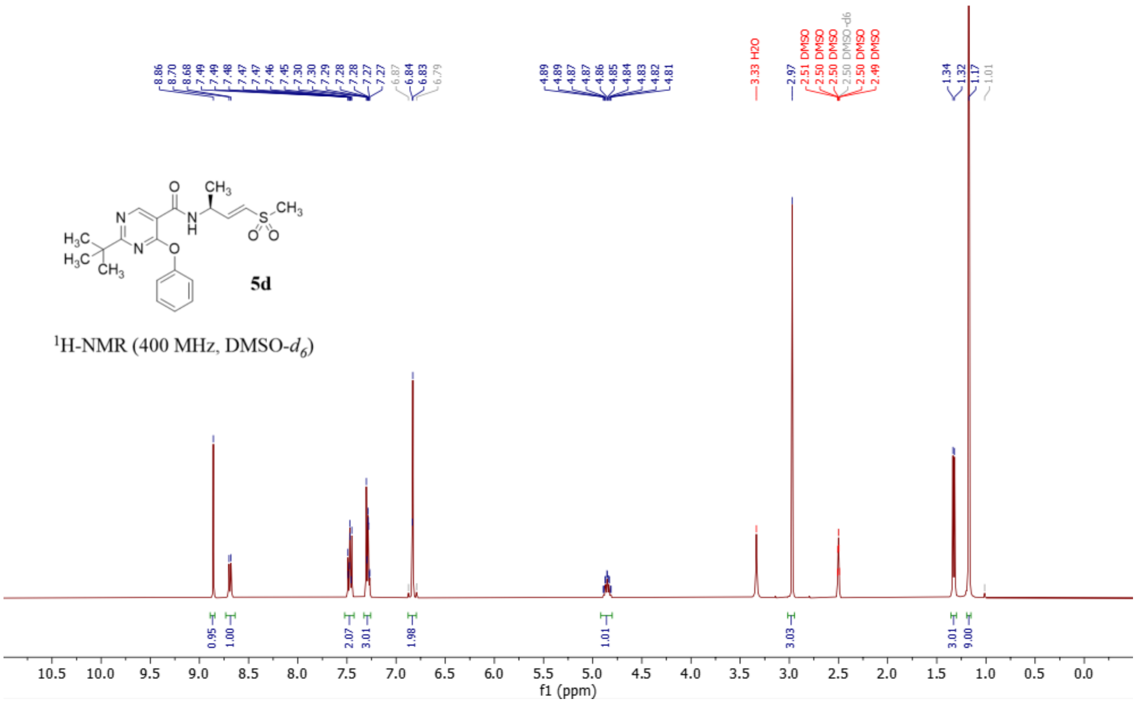

(d)

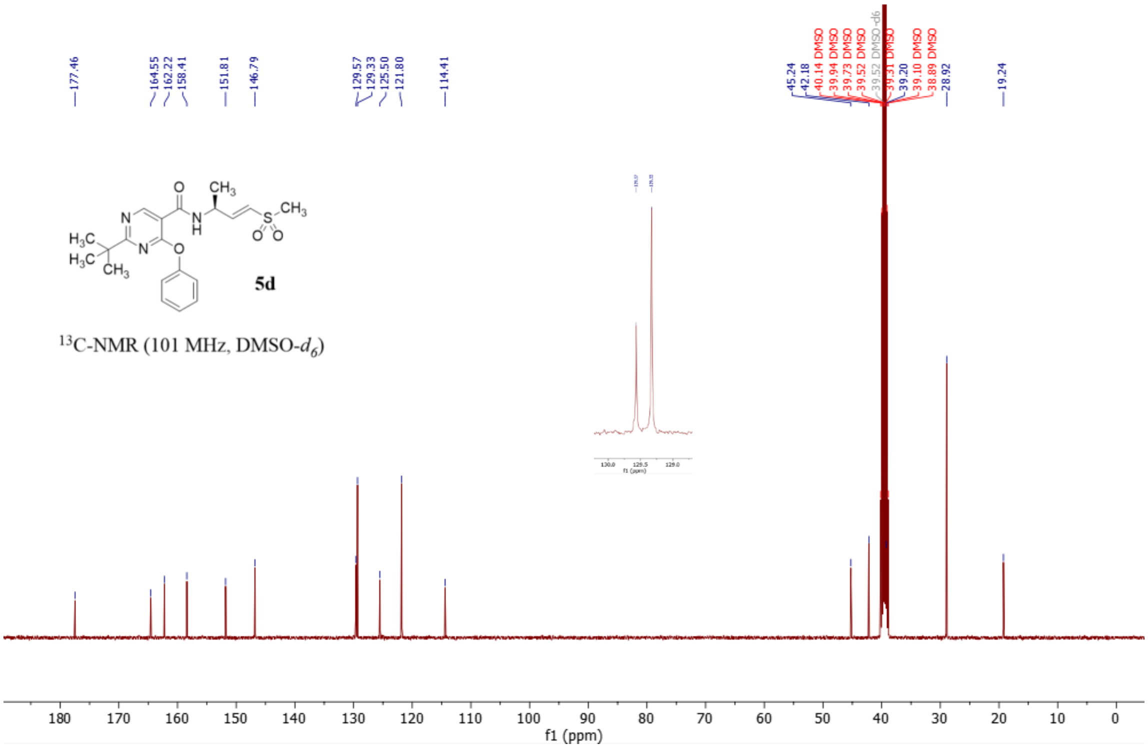

Figure S2. Additional analytical data for compound **5d**. (a) LC-MS report, (b) SFC report, (c) ^{1}H-NMR, (d) ^{13}C-NMR.

(a)

LCMS REPORT

Compound ID : 1
Sample ID : WUXI-228-288-1C
Injection Date : 6/16/2021 10:40:55 AM
Injection Vol : 1ul
Location : tray1 vail11
Acq Method : DELIVER-220.lcm
Org Data Name : D:\data\2021\2106\210616\WUXI-228-288-1C.lcd
Instrument : AC

Method Info : Instrument: Shimadzu LC-30AD MSD:LCMS-2020
Column:Kinetex EVO C18 5um 2.1*30mm
Column Temp:40
Mobile Phase:A:0.04%TFA in H2O
Mobile Phase:B:0.02%TFA in ACN
Flow Rate:0.8 ml/min

Time	B%	Flow(ml/min)
0.01	5	0.8
1.60	95	0.8
2.50	100	0.8
2.52	5	0.8
3.00	5	0.8

Chromatogram

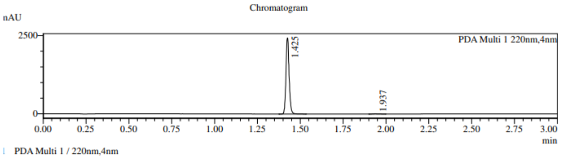

MS Chromatogram

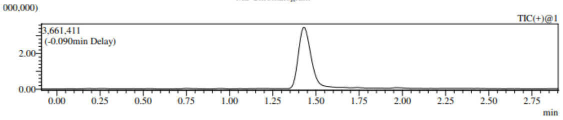

Integration Result

Peak Table

PDA Ch1 220nm

Peak#	Ret. Time	Height	Height%	USP Width	Area	Area%
1	1.425	2394693	99.723	0.035	3122580	99.508
2	1.937	6660	0.277	0.061	15450	0.492

(b)

SAMPLE INFORMATION

Sample ID:	WUXI-228-288-1D_AD_MeOH_H	Acquired By:	System
Compound ID:	LIB362-4-581	Sample Set Name:	20210617_EE
Acq Method :	AD_MeOH_IPAm_5_50_34_35_3min	Processing Method:	PEAK2
Date Acquired:	6/17/2021 6:12:51 PM CST	Injection Volume:	0.60 ul
Date Processed:	6/18/2021 9:27:00 AM CST	Vial:	1:B,6
Channel Name:	220	Run Time:	3.0 Minutes
Proc. Chnl. Descr.:	PDA Spectrum PDA 220.0 nm (PDA Spectrum	Raw Data:	D:\Data\ID_m32_result_ 40516
Instrument:	CAS-TJ-ANA-SFC-H (Waters UPCC with SQ Detector 2)		
Label:	No Racemate *		

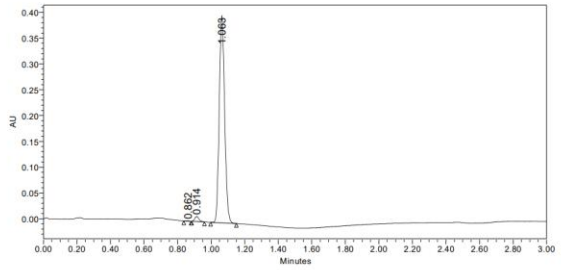

	RT	Area	% Area*	Height
1	0.862	226	0.02	179
2	0.914	16921	1.84	9996
3	1.063	903421	98.14	401420

(c)

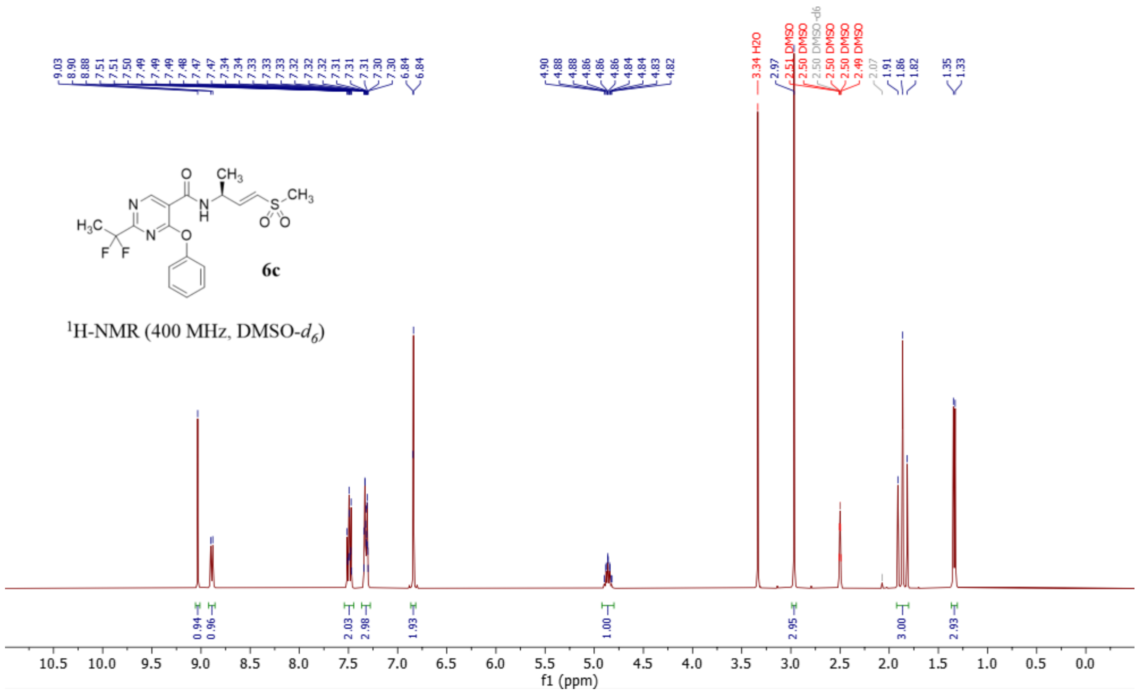

(d)

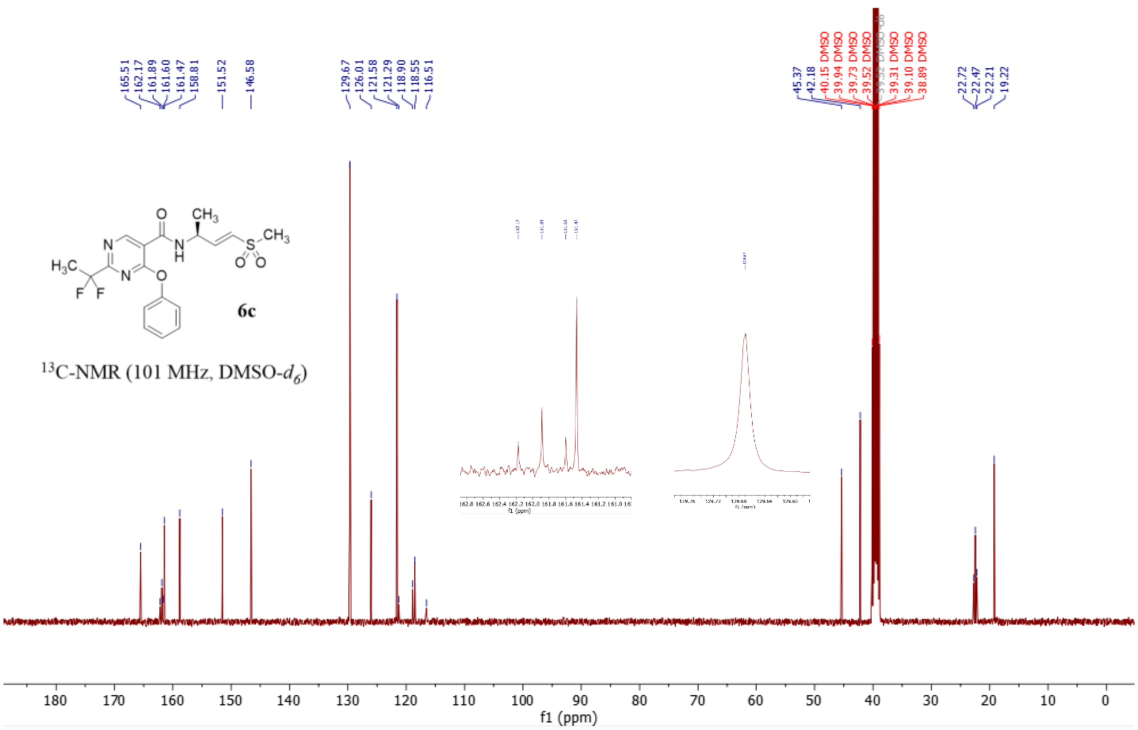

(e)

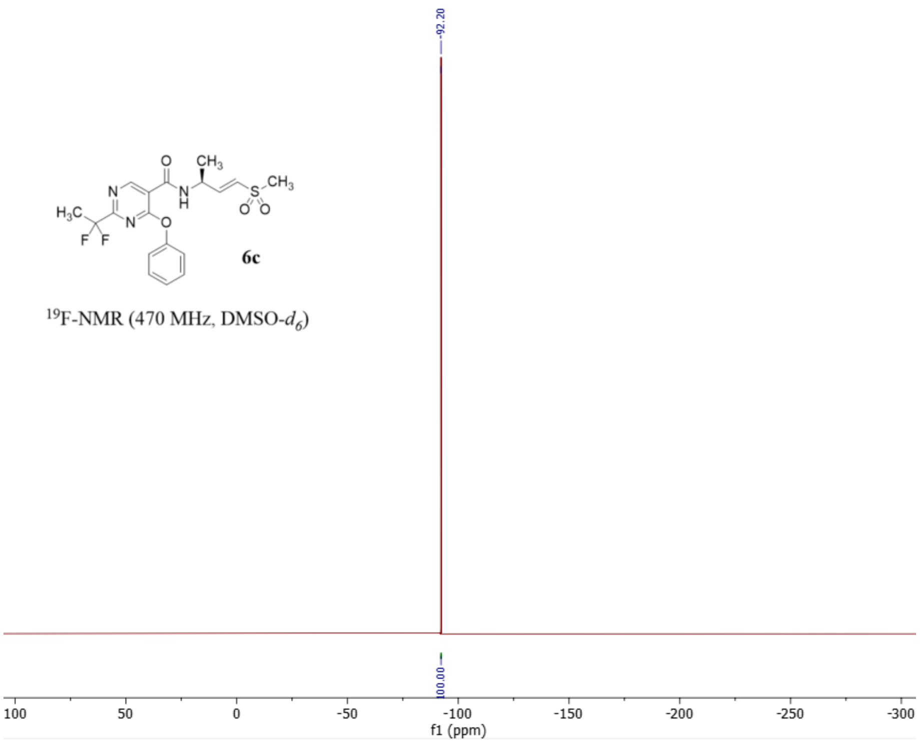

Figure S3. Additional analytical data for compound **6c**. (a) LC-MS report, (b) SFC report, (c) ^{1}H-NMR, (d) ^{13}C-NMR, (e) ^{19}F-NMR.

(a)

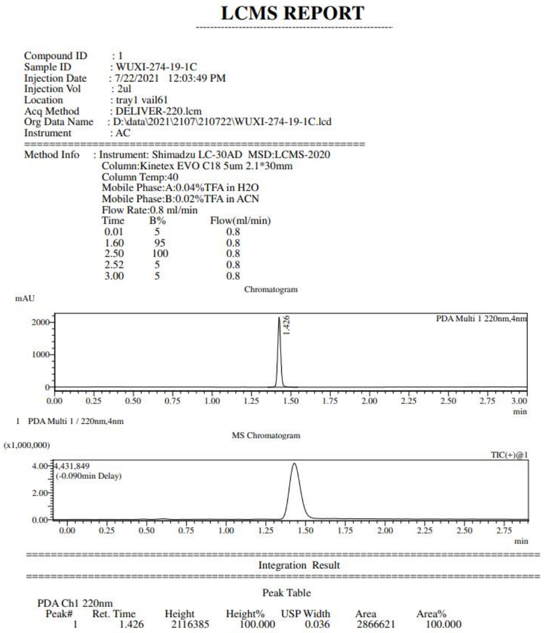

(b)

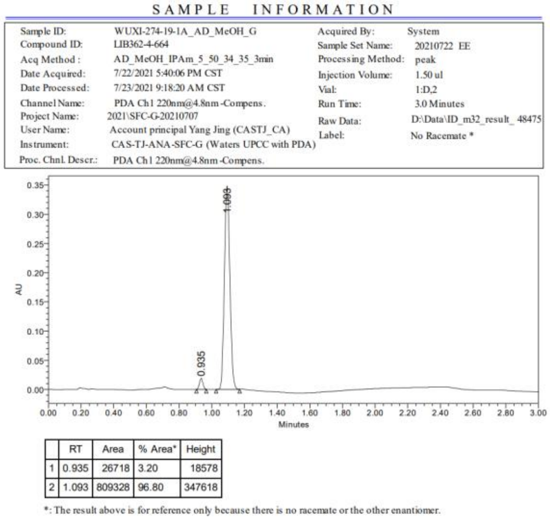

(c)

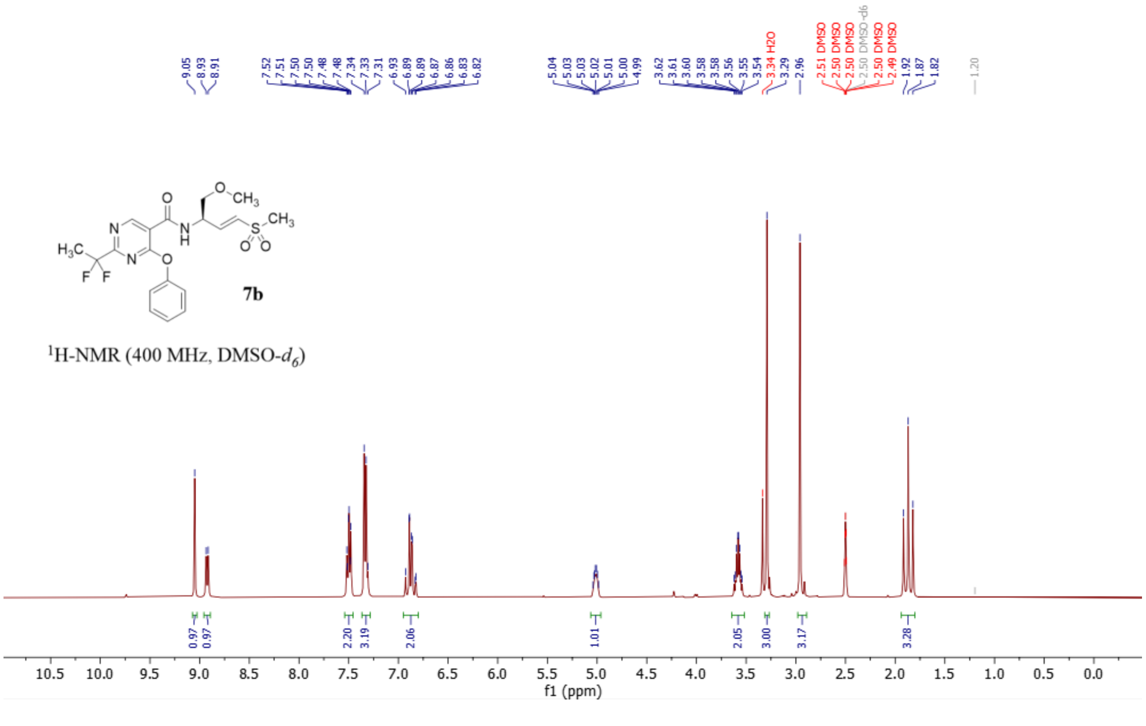

^{1}H-NMR (400 MHz, DMSO-d_6)

(d)

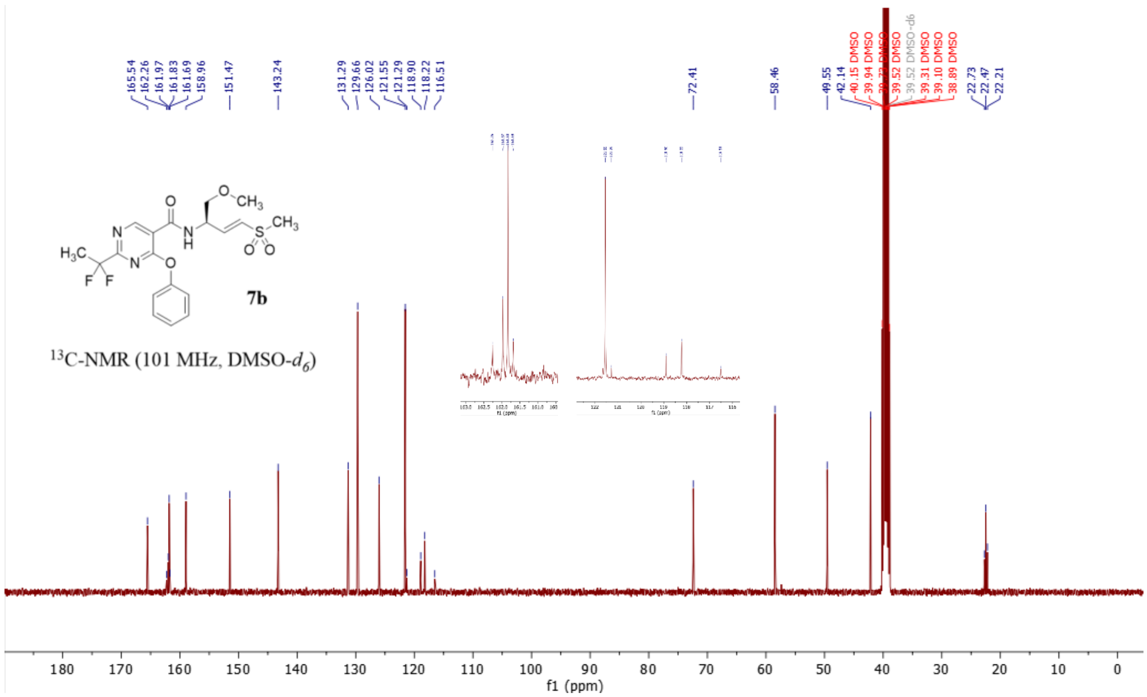

(e)

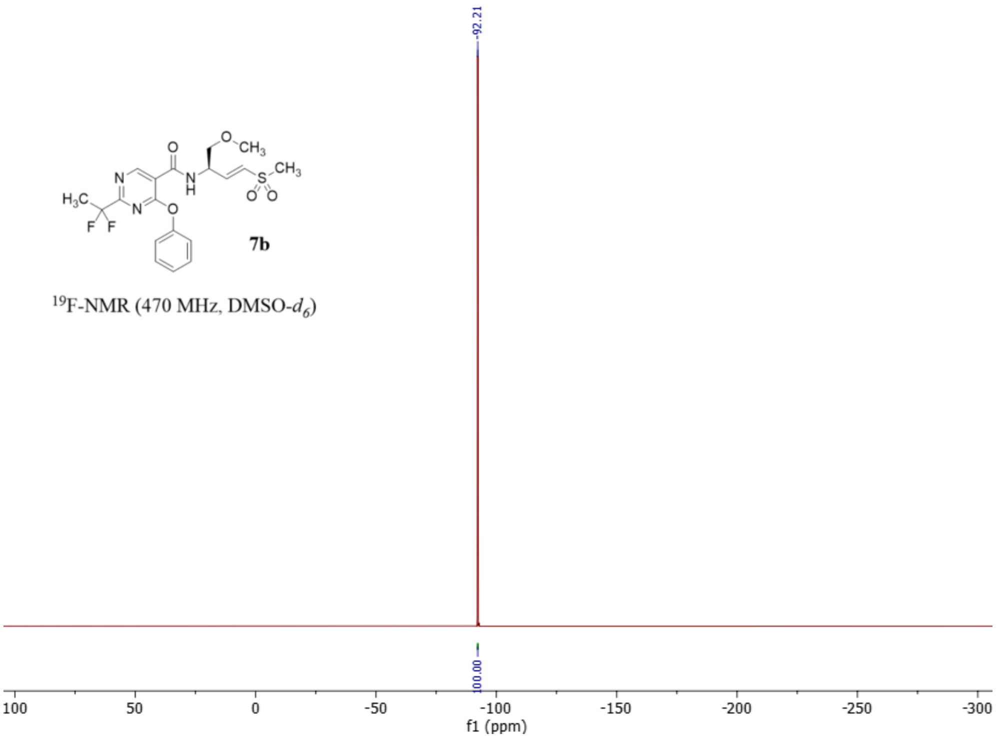

Figure S4. Additional analytical data for compound **7b**. (a) LC-MS report, (b) SFC report, (c) ^{1}H-NMR, (d) ^{13}C-NMR, (e) ^{19}F-NMR.

(a)

LCMS REPORT

Compound ID : LIB362-4-641
Sample ID : WUXI-277-35-1a3
Injection Date : 8/12/2021 11:48:12 AM
Injection Vol : 1ul
Location : tray1 vail86
Acq Method : DELIVER-220.lcm
Org Data Name : D:\data\2021\2108\210812\WUXI-277-35-1a3.lcd
Instrument : AC

Method Info : Instrument: Shimadzu LC-30AD MSD:LCMS-2020
Column:Kinetex EVO C18 5um 2.1*30mm
Column Temp:40
Mobile Phase:A:0.04%TFA in H2O
Mobile Phase:B:0.02%TFA in ACN
Flow Rate:0.8 ml/min

Time	B%	Flow(ml/min)
0.01	5	0.8
1.60	95	0.8
2.50	100	0.8
2.52	5	0.8
3.00	5	0.8

Chromatogram

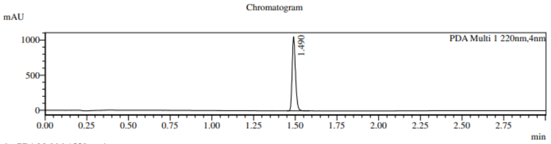

1 PDA Multi 1 / 220nm,4nm

MS Chromatogram

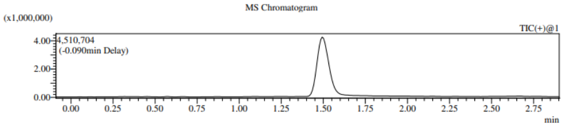

Integration Result

Peak Table

PDA Ch1 220nm

Peak#	Ret. Time	Height	Height%	USP Width	Area	Area%
1	1.490	1033237	100.000	0.037	1450170	100.000

(b)

SAMPLE INFORMATION

Sample ID:	WUXI-277-35-1s1_OD_MeOH_G	Acquired By:	System
Compound ID:	LIB362-4-641	Sample Set Name:	20210812 E
Acq Method :	OD_MeOH_IPAm_5_50_34_35_3min	Processing Method:	peak
Date Acquired:	8/12/2021 11:15:03 PM CST	Injection Volume:	1.50 ul
Date Processed:	8/13/2021 11:06:00 AM CST	Vial:	2:D,2
Channel Name:	PDA Ch1 220nm@4.8nm -Compens.	Run Time:	3.0 Minutes
Project Name:	2021\SFC-G-20210809	Raw Data:	D:\Data\ID_m32_result_ 14090
User Name:	CASTJ_CA (CASTJ_CA)	Label:	No Racemate *
Instrument:	CAS-TJ-ANA-SFC-G (Waters UPCC with PDA)		
Proc. Chnl. Descr.:	PDA Ch1 220nm@4.8nm -Compens.		

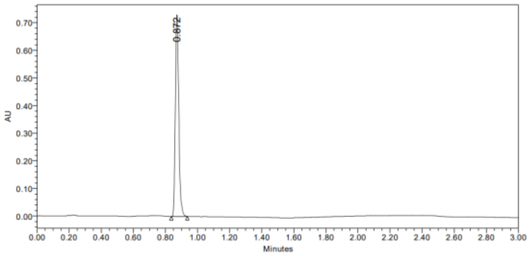

	RT	Area	% Area*	Height
1	0.872	1072336	100.00	734606

*: The result above is for reference only because there is no racemate or the other enantiomer.

(c)

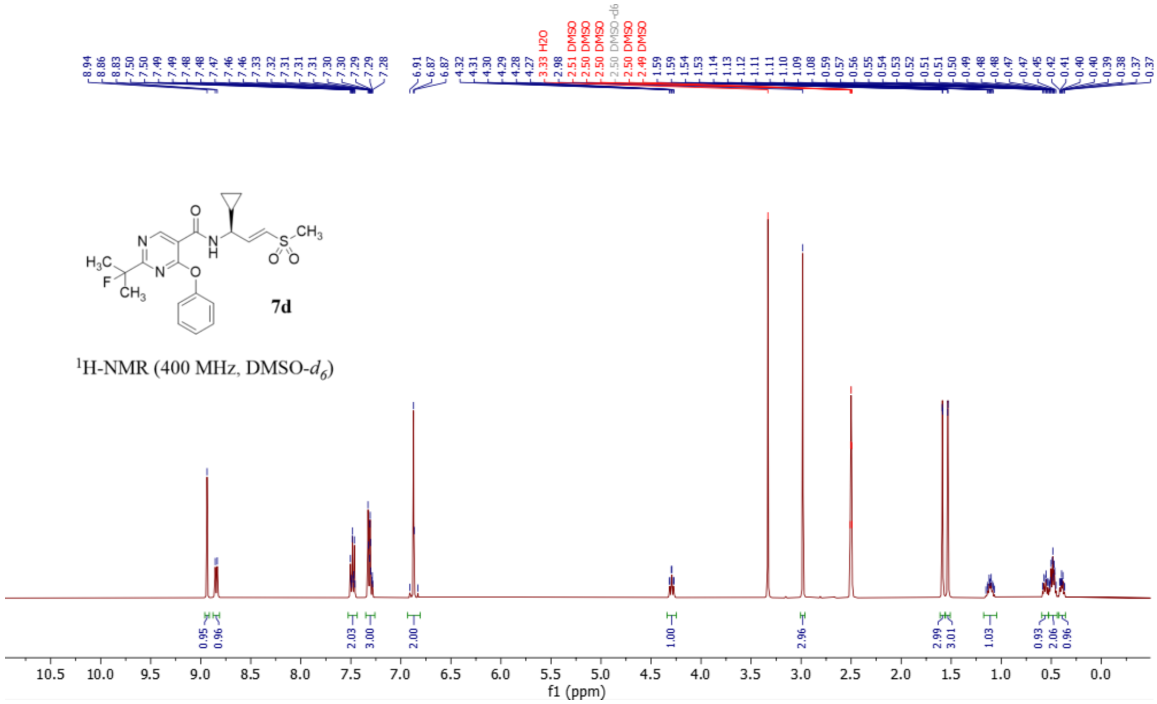

(d)

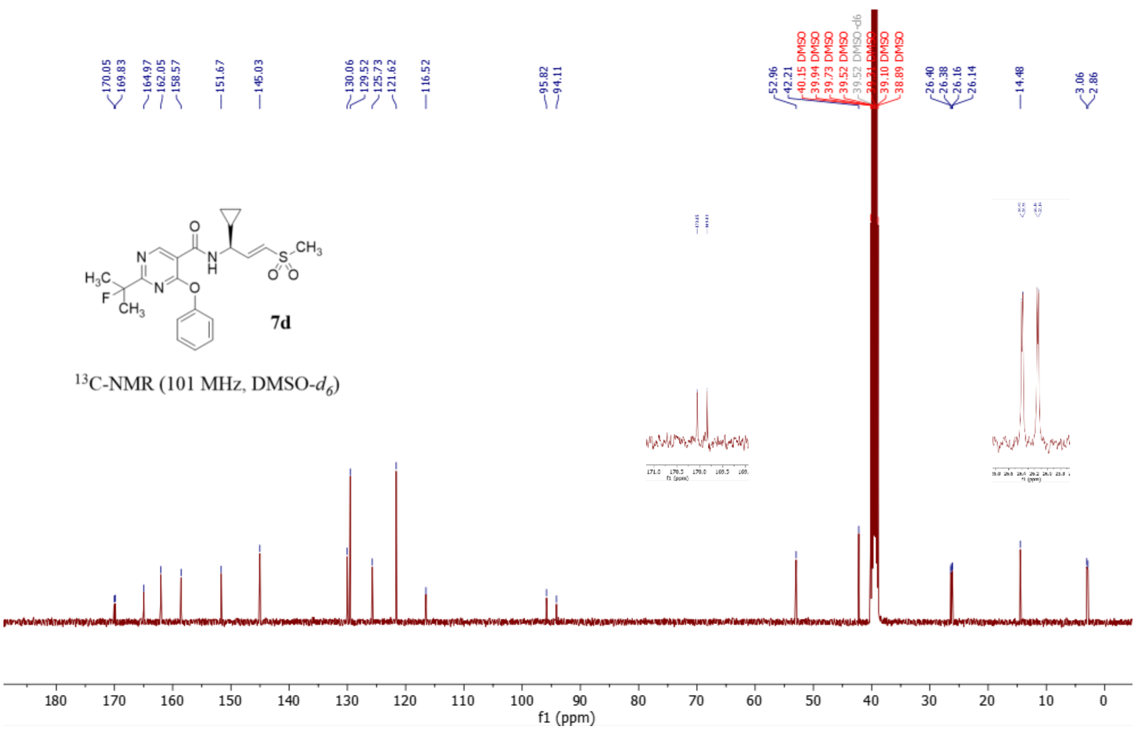

(e)

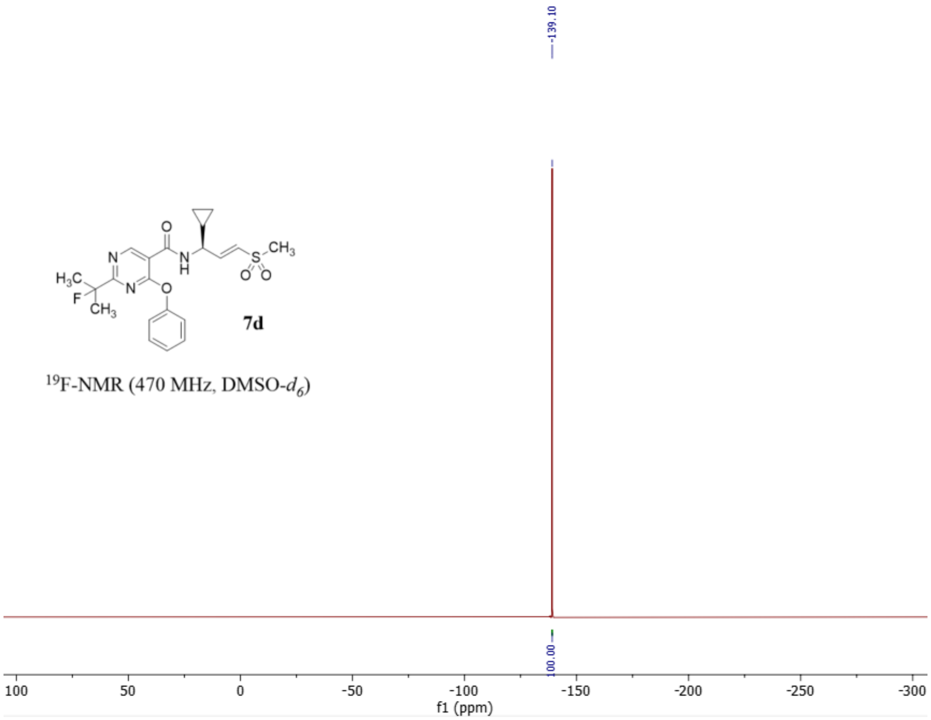

Figure S5. Additional analytical data for compound **7d**. (a) LC-MS report, (b) SFC report, (c) ^{1}H-NMR, (d) ^{13}C-NMR, (e) ^{19}F-NMR.

(a)

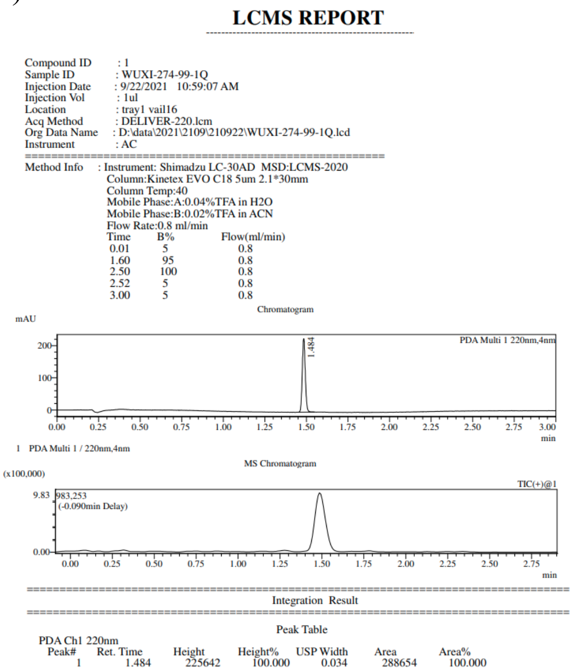

(b)

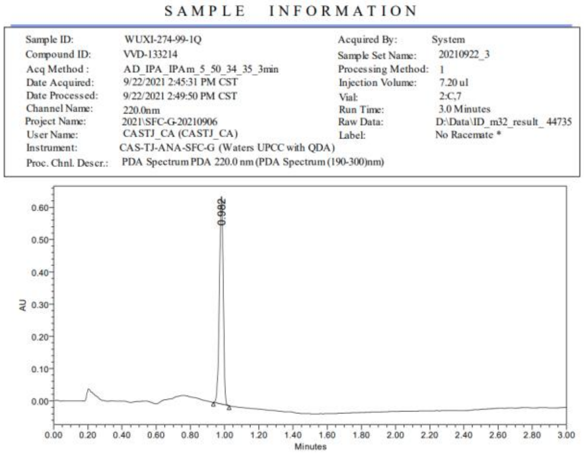

	RT	Area	% Area*	Height
1	0.982	991597	100.00	648912

(c)

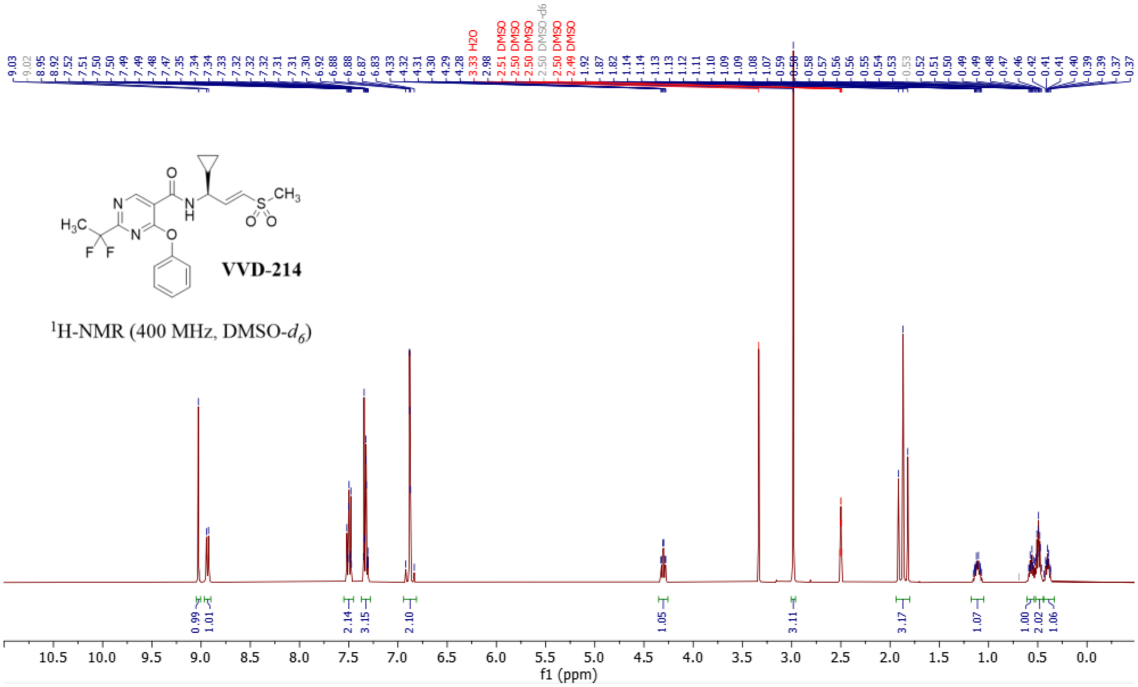

(d)

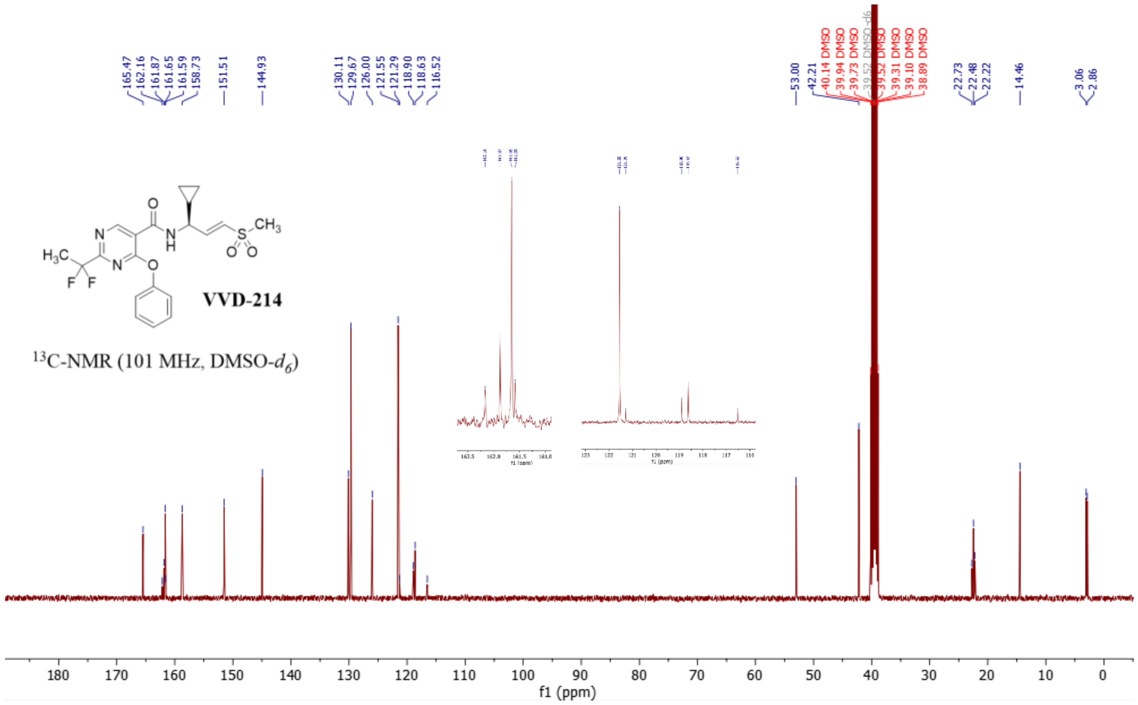

(e)

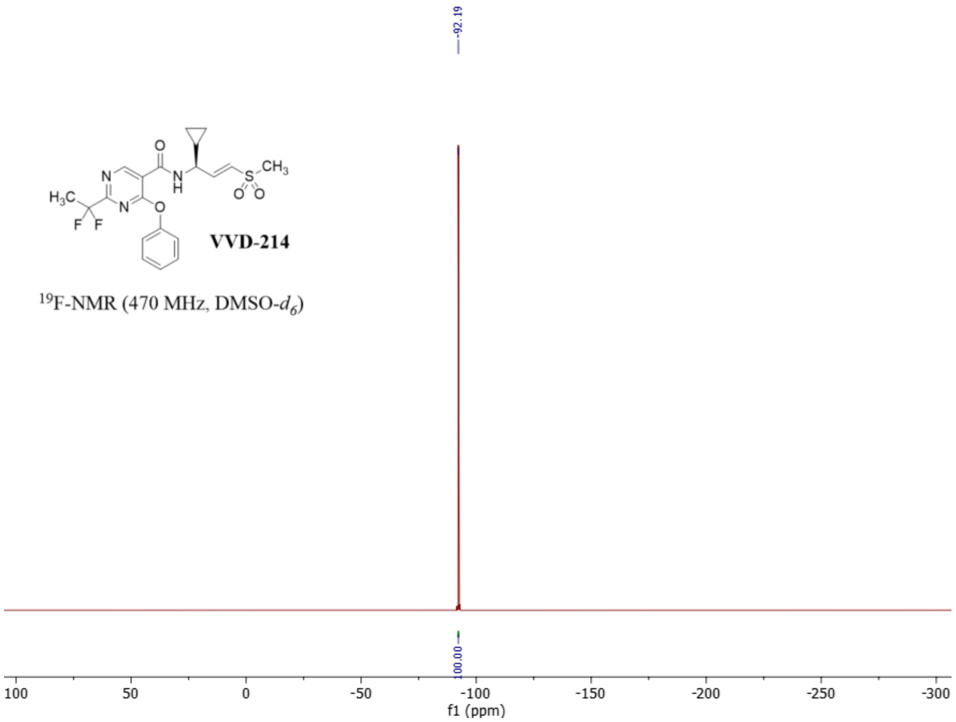

Figure S6. Additional analytical data for **VVD-214**. (a) LC-MS report, (b) SFC report, (c) ^{1}H-NMR, (d) ^{13}C-NMR, (e) ^{19}F-NMR

A$_{NMR}$ Calculations

Table S1: A$_{NMR}$ Values for compounds **2a, 3a-e**

Compound ID	δ (NH in DMSO-d_6)	δ (NH in Chloroform-*d*)	A$_{NMR}$ Value
**2a**	8.66	7.57	0.15
**3a**	8.53	7.92	0.09
**3b**	8.59	7.53	0.15
**3c**	8.74	7.88	0.12
**3d**	8.93	7.79	0.16
**3e**	9.13	8.10	0.14

$(A_{NMR} \text{ value}) = 0.0065 + 0.133 \times [\delta(\text{DMSO}) - \delta(\text{CDCl}_3)]$

(a)

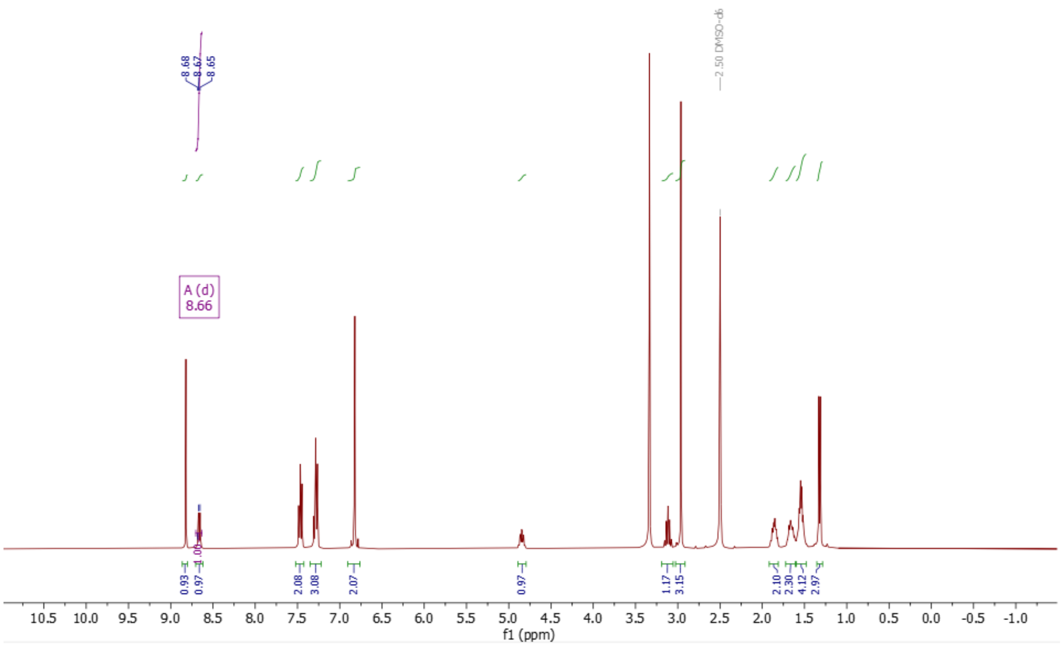

(b)

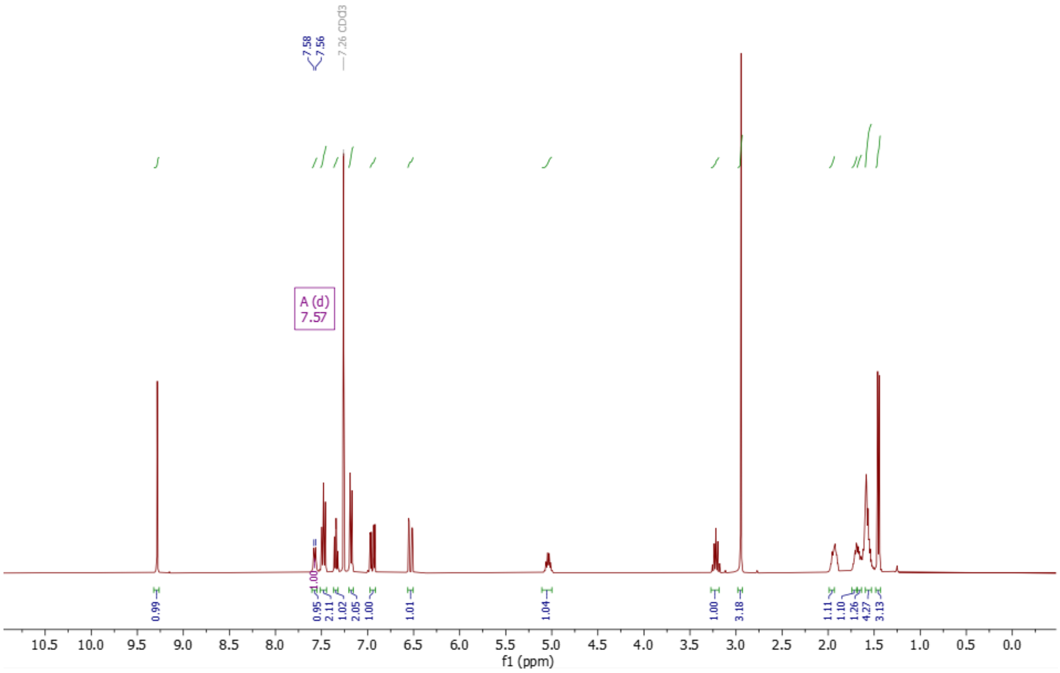

Figure S7. ^{1}H-NMR of compound **2a** in (a) DMSO-d_6 and (b) Chloroform-*d*.

(A_{NMR} value for **2a**) = 0.0065+0.133×(8.66-7.57) = **0.15**

(a)

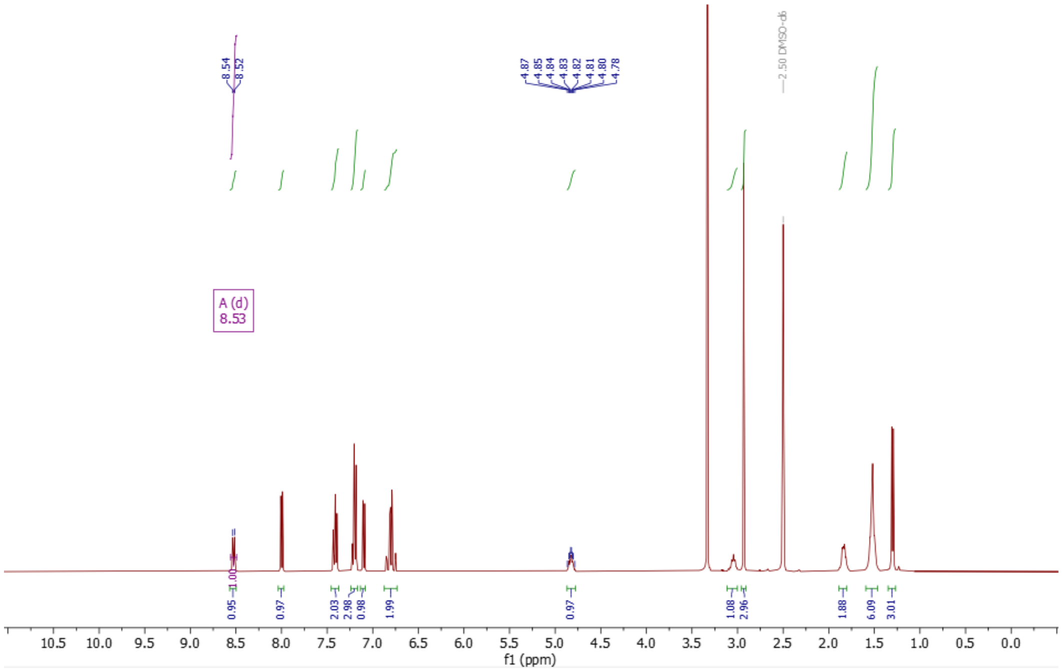

(b)

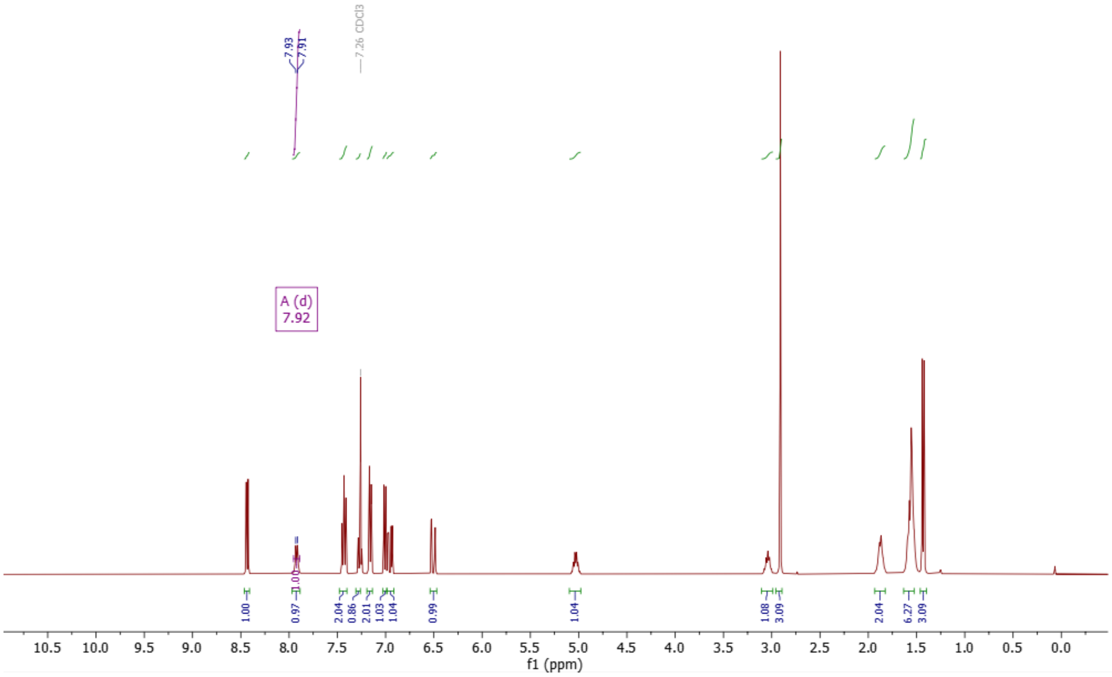

Figure S8. ^{1}H-NMR of compound **3a** in (a) DMSO-d_6 and (b) Chloroform-d.

(A_{NMR} value for **3a**) = 0.0065+0.133×(8.53-7.92) = **0.09**

(a)

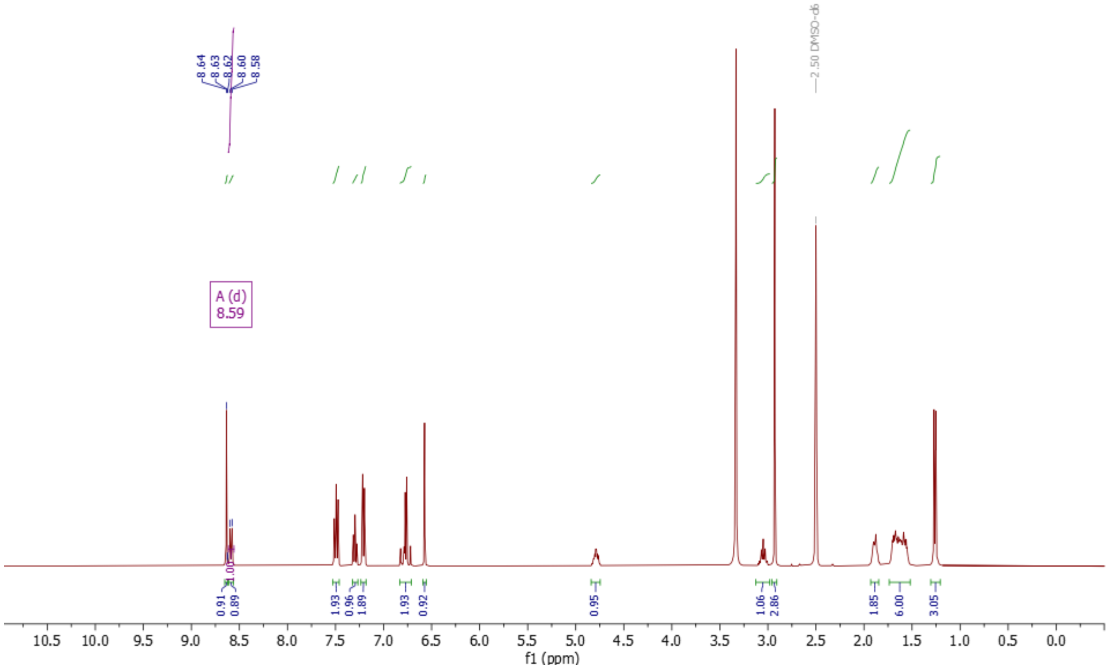

(b)

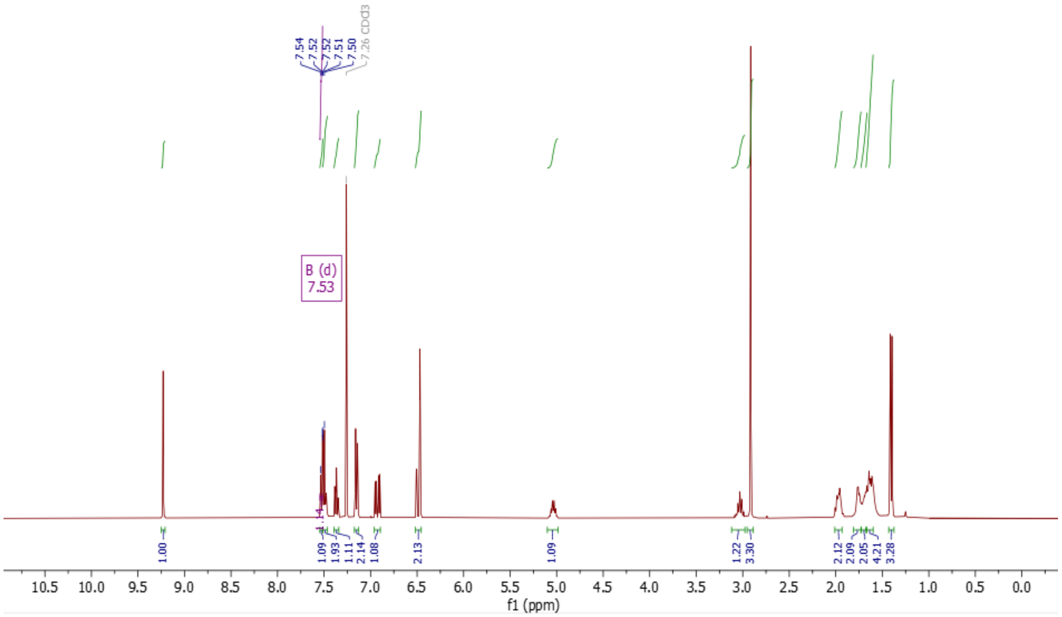

Figure S9. ^{1}H-NMR of compound **3b** in (a) DMSO-d_6 and (b) Chloroform-*d*.

(A_{NMR} value for **3b**) = 0.0065+0.133×(8.59-7.53) = **0.15**

(a)

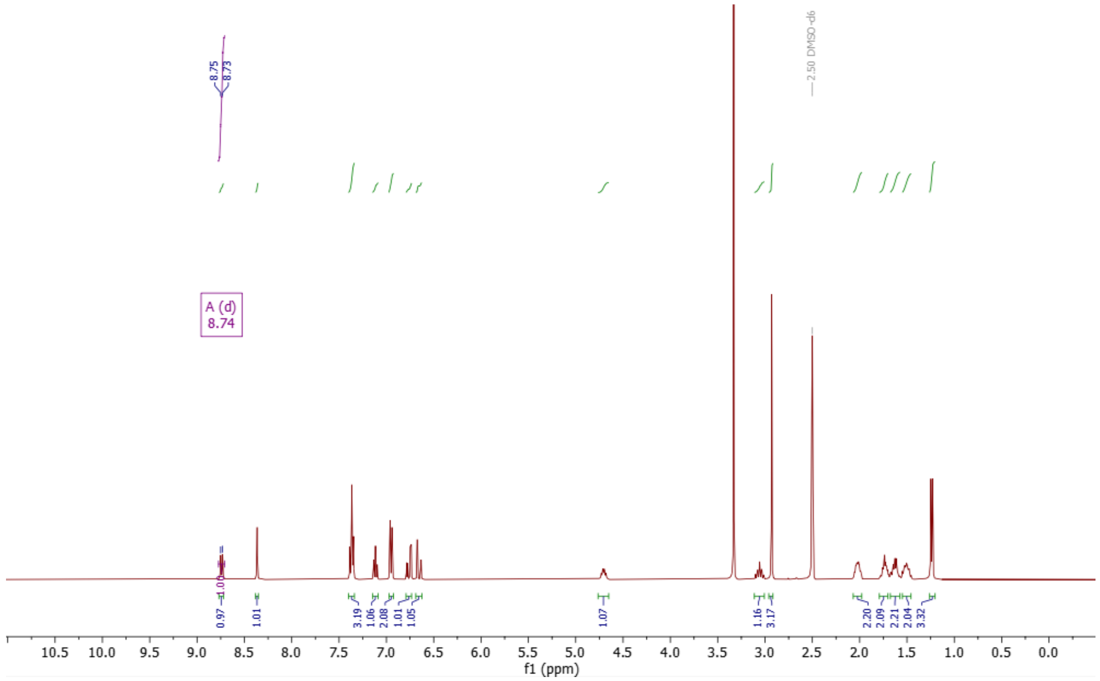

(b)

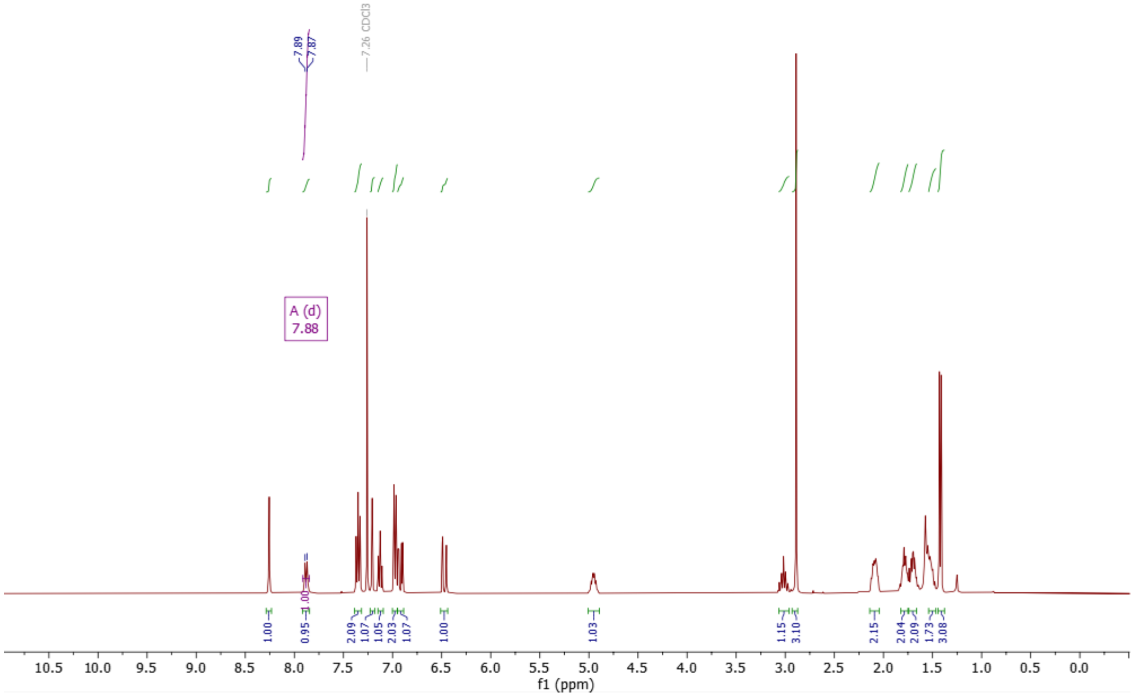

Figure S10. ^{1}H-NMR of compound **3c** in (a) DMSO-d_6 and (b) Chloroform-d.

(A_{NMR} value for **3c**) = 0.0065+0.133×(8.74-7.88) = **0.12**

(a)

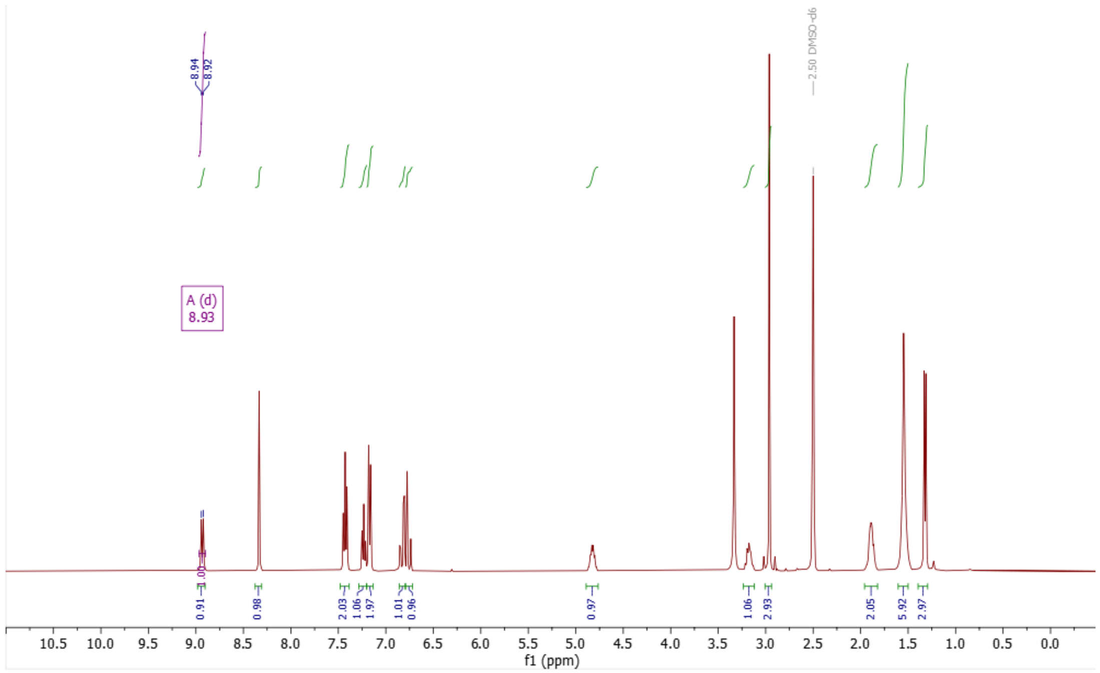

(b)

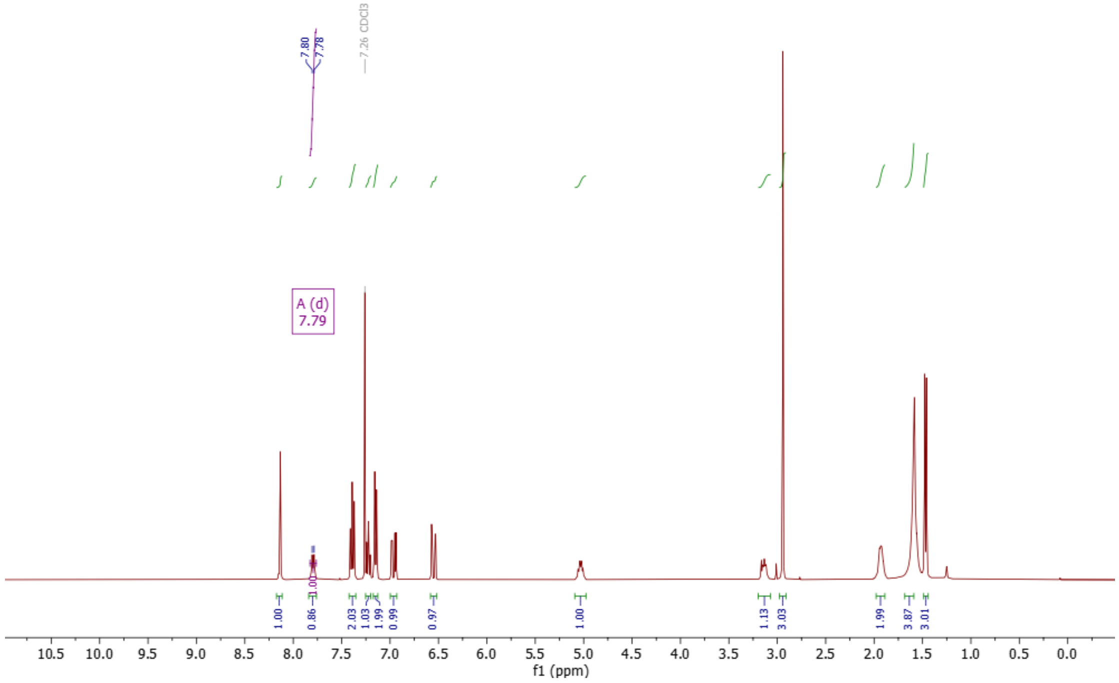

Figure S11. ^{1}H-NMR of compound **3d** in (a) DMSO-d_6 and (b) Chloroform-d.

(A_{NMR} value for **3d**) = 0.0065+0.133×(8.93-7.79) = **0.16**

(a)

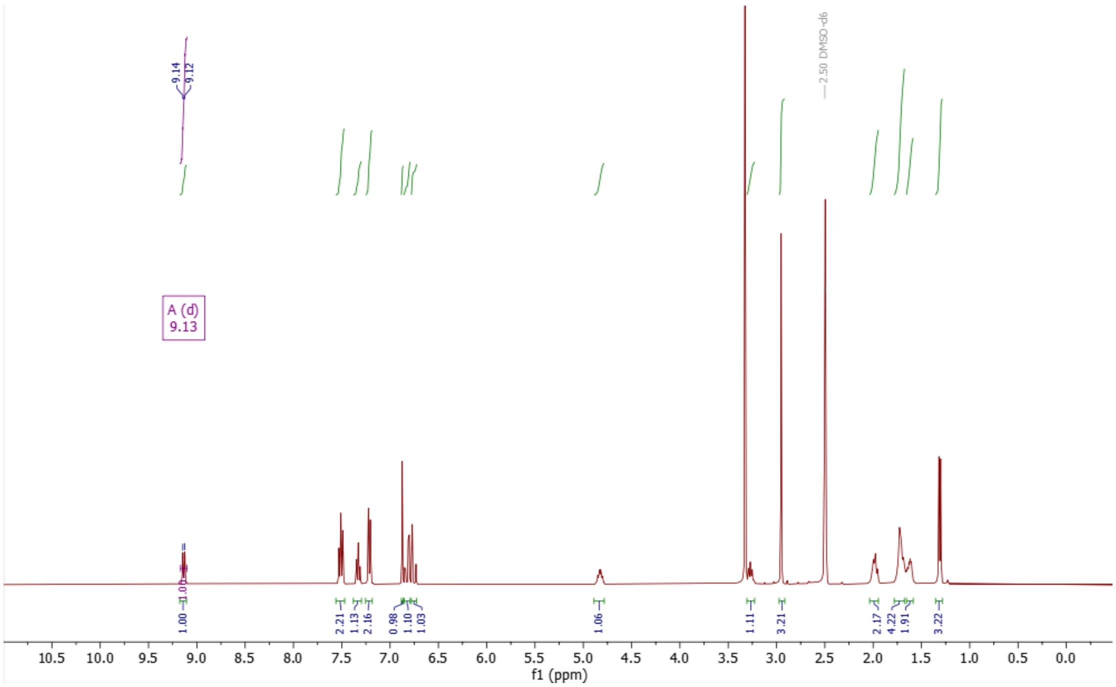

(b)

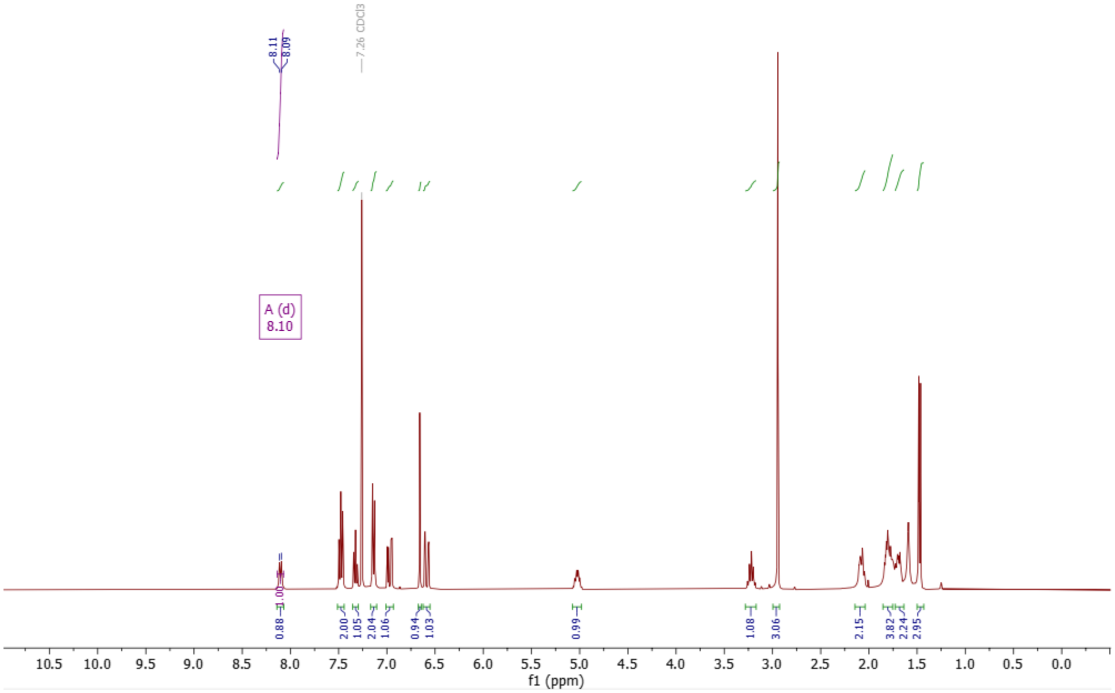

Figure S12. ^{1}H-NMR of compound **3e** in (a) DMSO-d_6 and (b) Chloroform-d.

(A_{NMR} value for **3e**) = 0.0065+0.133×(9.13-8.10) = **0.14**

Table S2. Additional SAR of Pyrimidine Ether

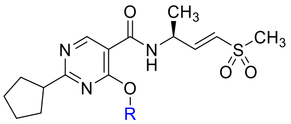

Cmpd. ID	Structure R	Helicase IC$_{50}$ [μM][a] +0.2 mM ATP	Cellular Growth Inhibition, GI$_{50}$ [μM][b] HCT116	SW480
**10a**	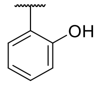	3.4	0.98	>10
**10b**	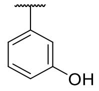	11	5.1	>10
**10c**	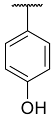	70	--	--
**10d**	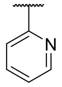	44	--	--
**10e**	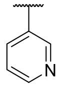	12	3.1	>10
**10f**	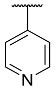	>100	--	--
**10g**	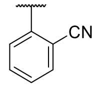	>100	--	--
**10h**	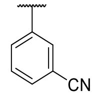	>100	--	--
**10i**	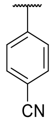	14	>10	>10

[a]Inhibition of DNA unwinding by hWRN519-1227. IC$_{50}$ data are determined in a 30-minute assay after a 30-minute pre-incubation of the compound and WRN protein. Values shown are the means of at least n=2 independent measurements. Each has a SEM ±0.2 log unit. [b]Growth inhibition of HCT116 (MSI-H) and SW480 (MSS) after 5 days of compound treatment. All GI$_{50}$ data are the means of at least n=2 independent measurements. Each has a SEM ±0.2 log unit.

TE Time-course for Rate Determination

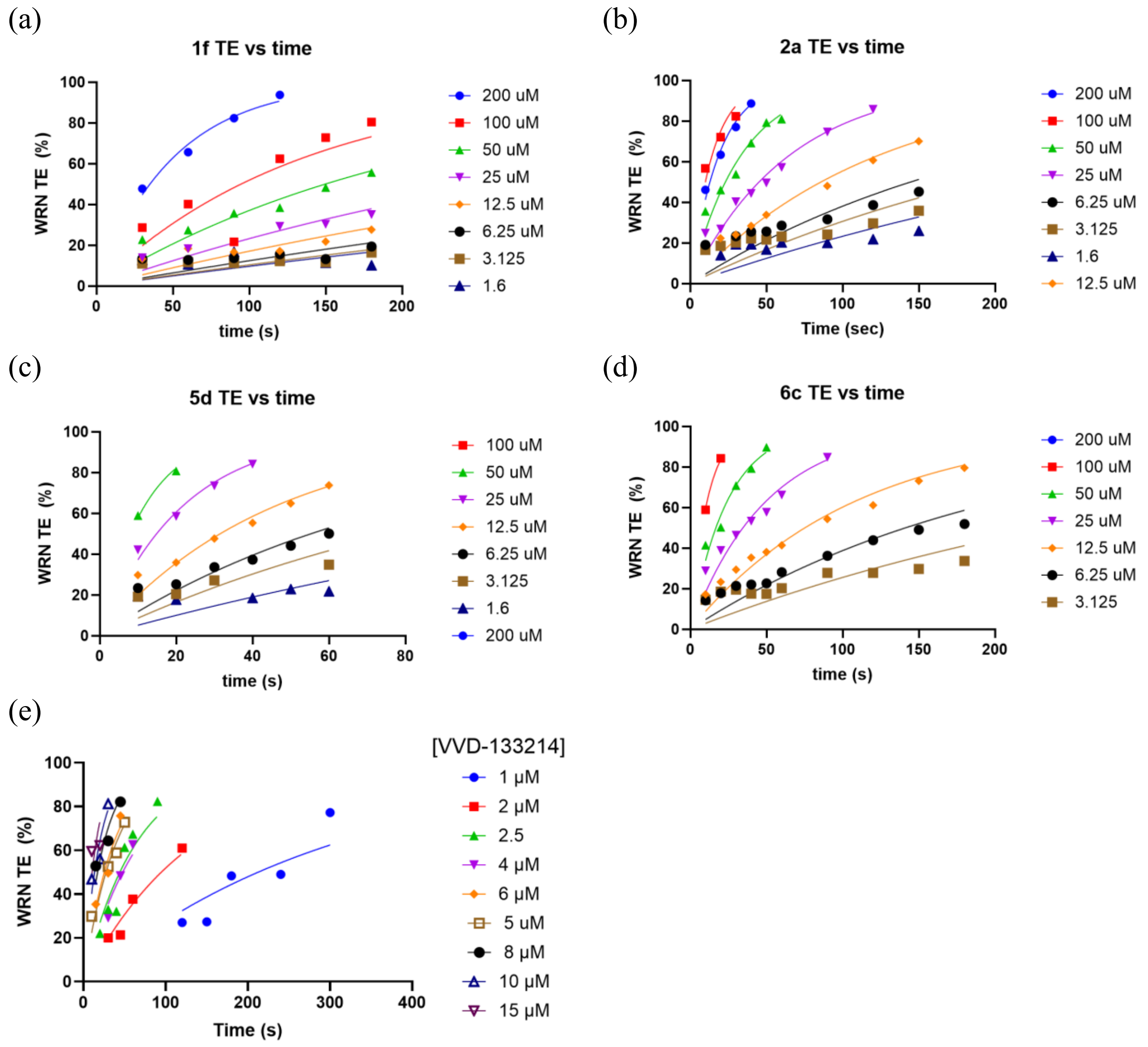

Figure S13. TE vs time plot for compounds (a) **1f**, (b) **2a**, (c) **5d**, (d) **6c**, (e) **VVD-214**.

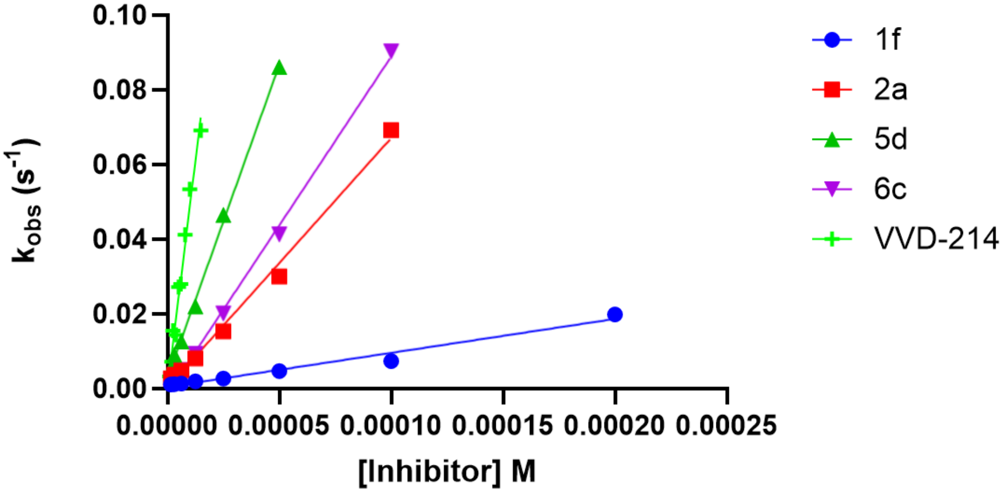

Figure S14. k_{obs} vs concentration plot for compounds **1f**, **2a**, **5d**, **6c**, and **VVD-214**.
